# PRRT2 Is a Key Component of the Ca^2+^-Dependent Neurotransmitter Release Machinery

**DOI:** 10.1016/j.celrep.2016.03.005

**Published:** 2016-03-24

**Authors:** Pierluigi Valente, Enrico Castroflorio, Pia Rossi, Manuela Fadda, Bruno Sterlini, Romina Ines Cervigni, Cosimo Prestigio, Silvia Giovedì, Franco Onofri, Elisa Mura, Fabrizia C. Guarnieri, Antonella Marte, Marta Orlando, Federico Zara, Anna Fassio, Flavia Valtorta, Pietro Baldelli, Anna Corradi, Fabio Benfenati

**Affiliations:** 1 Department of Experimental Medicine, University of Genova, Viale Benedetto XV, 3, 16132 Genova, Italy; 2 Center for Synaptic Neuroscience and Technology, Istituto Italiano di Tecnologia, Largo Rosanna Benzi 10, 16132 Genova, Italy; 3 San Raffaele Scientific Institute and Vita Salute University, Via Olgettina 58, 20132 Milano, Italy; 4 Department of Neuroscience, Istituto Giannina Gaslini, Via Gerolamo Gaslini, 5, 16148 Genova, Italy

**Keywords:** PRRT2, knockdown, synaptic transmission, synchronous and asynchronous release, release probability, synaptotagmin

## Abstract

Heterozygous mutations in proline-rich transmembrane protein 2 (PRRT2) underlie a group of paroxysmal disorders, including epilepsy, kinesigenic dyskinesia, and migraine. Most of the mutations lead to impaired PRRT2 expression, suggesting that loss of PRRT2 function may contribute to pathogenesis. We show that PRRT2 is enriched in presynaptic terminals and that its silencing decreases the number of synapses and increases the number of docked synaptic vesicles at rest. PRRT2-silenced neurons exhibit a severe impairment of synchronous release, attributable to a sharp decrease in release probability and Ca^2+^ sensitivity and associated with a marked increase of the asynchronous/synchronous release ratio. PRRT2 interacts with the synaptic proteins SNAP-25 and synaptotagmin 1/2. The results indicate that PRRT2 is intimately connected with the Ca^2+^-sensing machinery and that it plays an important role in the final steps of neurotransmitter release.

## Highlights

• PRRT2 is a presynaptic protein
• PRRT2 is required for synchronous neurotransmitter release
• PRRT2 silencing decreases synaptic density and release probability
• PRRT2 interacts with the fast Ca^2+^ sensors synaptotagmin 1/2

## Graphical Abstract

**Figure:**
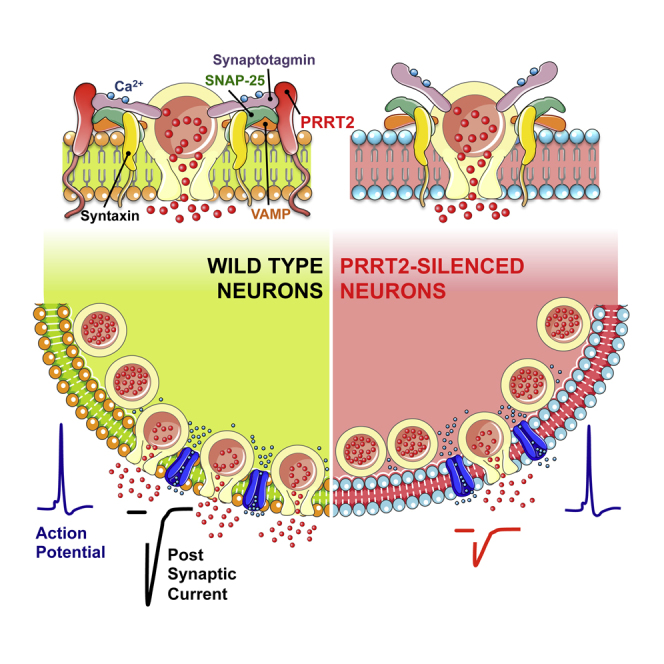

## Teaser

Valente et al. show that PRRT2, a single causative gene for a group of paroxysmal neurological diseases, is a key component of regulated exocytosis. Silencing PRRT2 dramatically impairs neurotransmitter release by markedly reducing release probability. PRRT2 interacts with the fast Ca^2+^ sensors synaptotagmin 1/2 and endows the SNARE complex with Ca^2+^ sensitivity.

## Introduction

Over the last 4 years, several studies have identified an array of heterozygous nonsense, frameshift, and missense mutations in the gene encoding proline-rich transmembrane protein 2 (PRRT2) in a large number of cases affected by different paroxysmal disorders such as benign familial infantile seizures, infantile convulsion choreoathetosis, migraine, hemiplegic migraine, paroxysmal kinesigenic dyskinesia/choreoathetosis, benign familial infantile seizures/epilepsy, and episodic ataxia (Chen et al., 2011, Lee et al., 2012; for review, see Ebrahimi-Fakhari et al., 2015). The astonishing pleiotropy of the phenotypic expression of PRRT2 mutations points to an overlap in the pathogenic pathways and to a very important role of this gene in regulating synaptic transmission and network activity.

The PRRT2 gene is located in human chromosome 16 and consists of four exons encoding a poorly characterized protein of 340 amino acids. Most mutations identified in PRRT2 are phenotypically highly penetrant and cause truncation of the protein because of nonsense or frameshifts mutations that result in mRNA degradation by nonsense-mediated mRNA decay or degradation of the protein (Wu et al., 2014), suggesting a loss-of-function mechanism of action.

PRRT2 mRNA has been shown to be almost exclusively expressed in neurons in the cortex, hippocampus, basal ganglia, and cerebellum (Chen et al., 2011, Lee et al., 2012, Heron et al., 2012, Trabzuni et al., 2011), which are all regions putatively involved in the pathogenesis of the PRRT2-linked diseases. The functional role of this protein is totally unknown. A yeast two-hybrid screen highlighted a potential interaction of PRRT2 with synaptosomal-associated protein 25 kDa (SNAP-25), one of the presynaptic soluble N-ethylmaleimide sensitive factor (NSF) attachment protein receptor (SNARE) proteins triggering fusion of synaptic vesicles (SVs) (Stelzl et al., 2005, Lee et al., 2012). Recently, PRRT2 has also been found among proteins associated with α-amino-3-hydroxy-5-methyl-4-isoxazolepropionic acid (AMPA)-type glutamate receptors by a high-resolution proteomics study (Schwenk et al., 2014), and its mutation was associated with increased levels of extracellular glutamate (Li et al., 2015).

To investigate the role of PRRT2 in presynaptic physiology, we silenced PRRT2 expression in primary neurons using RNA interference. PRRT2 knockdown caused a marked impairment in synaptic transmission because of a decrease in the density of synaptic connections and a sharp reduction in release probability. Both effects were fully reversible upon re-expression of shRNA-resistant PPRT2. In addition to SNAP-25, the search for PRRT2 partners identified the fast Ca^2+^ sensors synaptotagmins 1/2, responsible for triggering synchronous neurotransmitter release. The results indicate that PRRT2 is intimately connected with the Ca^2+^-sensing machinery of neurotransmitter release.

## Results

### PRRT2 Is a Presynaptic Protein Developmentally Expressed during Synaptogenesis

We evaluated the regional expression of PRRT2 protein in various areas of the adult mouse brain and the developmental expression profile of PRRT2 in both mouse brain and primary neuronal cultures (Figure S1). PRRT2 expression was prominent throughout the adult brain, with the highest levels in the cerebellum, basal ganglia, and neocortex (Figure S1A). In the cerebral cortex and hippocampus, the PRRT2 gene was already expressed at early postnatal stages, and its expression increased to reach a plateau at 1 month of life, a period of intense synapse formation and rearrangement (Figures S1B and S1C). The same pattern was reproduced in primary hippocampal and cortical neurons, where PRRT2 expression, already discernible at early stages, was greatly enhanced between 10 and 21 days in vitro (DIVs), a temporal window of intense synaptogenesis (Figures S1B and S1D). In contrast, PRRT2 expression was almost undetectable in primary astroglial cultures (data not shown), consistent with the neuron-specific expression of PRRT2.

**Figure 1:**
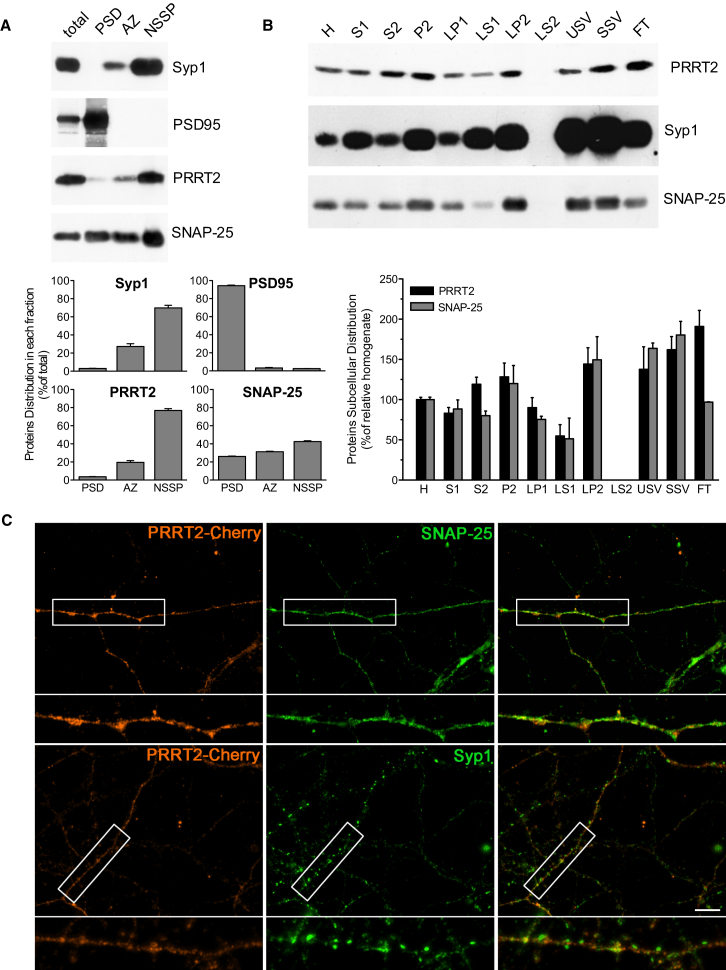
PRRT2 Is Localized at the Presynaptic Level (A) Ultrafractionation of brain synaptosomes. Purified synaptosomes from adult mouse brain were subjected to ultrasynaptic fractionation to separate the AZ, PSD, and NSSP made by extrinsic and integral membrane proteins of the nerve terminal. Aliquots of total synaptosomes and of each ultrasynaptic fraction (10–30 μg) were probed with antibodies against PRRT2, SNAP-25, and protein markers to validate ultrasynaptic compartments such as synaptophysin-1 (Syp1) and PSD95 (top). Immunoblots were quantified by densitometric analysis of the fluorograms, and the values are expressed in mean (± SEM) percentages of the total amount (bottom). The partition of PSD95, Syp1, and SNAP-25 in the corresponding fractions is shown. Note that PRRT2 preferentially partitioned in the NSSP fraction, similarly to Syp1 and SNAP-25. (B) Subcellular distribution of endogenous PRRT2 in neurons. Forebrain fractions obtained at various stages of SV purification were analyzed by western blotting using antibodies to PRRT2, SNAP-25, and Syp1 (top). H, homogenate; S1, post-nuclear supernatant; S2, supernatant of P2; P2, crude synaptosomes; LP1, crude synaptic plasma membranes; LS1, supernatant of LP1; LP2, crude synaptic vesicles; LS2, synaptosol; USV, highly purified synaptic vesicles; SSV, salt-treated highly purified synaptic vesicles; FT, flowthrough fraction containing small presynaptic membranes. Immunoblots were quantified as in (A), and the value of each subcellular fraction is expressed in percentage of homogenate as means ± SEM (bottom). (C) Localization of PRRT2 in mature neurons. Primary hippocampal neurons transduced at 10 DIVs with PRRT2-mCherry (red) were subjected to immunostaining at 15 DIVs with antibodies to PRRT2, SNAP-25, and Syp1. PRRT2 immunoreactivity (red) largely overlapped with the staining of the two presynaptic proteins in axonal and nerve terminal areas. Scale bar, 10 μm. See also Figure S1.

To verify the subcellular distribution of PRRT2 at the synapse, we fractionated purified synaptosomes to isolate synaptic junctions, subsequently separated into active zone (AZ), postsynaptic density (PSD), and a fraction including extrinsic and integral membrane proteins associated with the presynaptic area (non-synaptic synaptosomal protein [NSSP]) (Phillips et al., 2001). PRRT2 was enriched in the NSSP fraction, like SNAP-25 and synaptophysin. Although the protein appeared to be substantially absent from the PSD, low levels were associated with the AZ fraction (Figure 1A). We further analyzed PRRT2 enrichment along a subcellular fractionation procedure, yielding highly purified SVs (Huttner et al., 1983). Interestingly, we found that SNAP-25 and PRRT2 had a similar subcellular distribution and associated with both SVs and presynaptic membrane fractions (LP2 and FT) (Figure 1B). These data confirm that PRRT2 can be considered a bona fide presynaptic protein and suggest that it could undergo trafficking between the presynaptic membrane and the SV compartments, as described previously for SNAP-25 (Walch-Solimena et al., 1995). The lack of reliable anti-PRRT2 antibodies for immunocytochemistry did not allow us to unambiguously visualize the endogenously expressed protein in tissue and primary neurons. Therefore, to test for the localization of the protein in neuronal cells, 10-DIVs hippocampal neurons were transduced with lentiviruses encoding for PRRT2-mCherry and immunostained at 15 DIVs. In agreement with the biochemical experiments, PRRT2 colocalized with SNAP-25 and synaptophysin, confirming its targeting to the synapse (Figure 1C).

To silence PRRT2 expression, five short hairpin RNAs (shRNAs) were designed on the basis of the open reading frame of the mouse PRRT2 transcript and inserted into lentiviral vectors expressing either TurboGFP (tGFP) or the mCherry cytosolic reporter for cell identification. Four shRNAs were effective in knocking down PRRT2 expression to very low levels in HeLa cells, whereas control scrambled shRNAs (Scr) were virtually ineffective (data not shown). The two most active PRRT2-shRNAs (Sh1 and Sh4) and two control (Scr) shRNAs were chosen for the subsequent studies. Because the tested shRNAs were directed against the PRRT2 coding sequence, we generated shRNA-resistant PRRT2 clones fused to mCherry for rescuing the PRRT2 knockdown. Infection of low-density mouse cortical neurons with the appropriate lentiviral vectors resulted in an effective silencing of PRRT2 and in the expression of the shRNA-resistant version of PRRT2 overriding the RNA interference (Figures S1E–S1I).

### Knockdown of PRRT2 in Low-Density Primary Neurons Decreases the Density of Synaptic Contacts and Alters the Synaptic Ultrastructure

**Figure 2:**
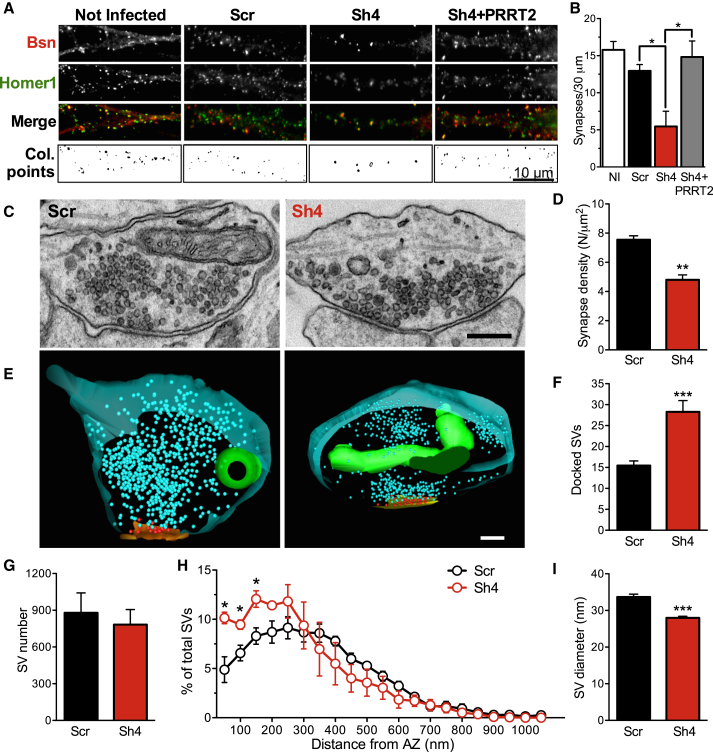
PRRT2 Knockdown Decreases Synapse Density and Increases Docked SVs in Low-Density Hippocampal Neurons (A) Representative images of dendrites of hippocampal neurons infected at 7 DIVs with Scr, Sh4, and Sh4 + Sh4-resistant PRRT2 (Sh4+PRRT2) or left uninfected and analyzed at 14 DIVs. Synaptic boutons were identified by double immunostaining for Bassoon (Bsn, red) and Homer1 (green). The colocalization panels (Col. points) highlight the double-positive puncta (black) corresponding to synapses. Scale bar, 10 μm. (B) Quantitative analysis of synaptic puncta counted on 30-μm dendrite tracts starting from the cell body in neurons treated as in (A). Data are means ± SEM from three independent experiments, each carried out in duplicate. Five dendrites for each neuron, from at least ten neurons for each sample, were counted. ^∗^p < 0.05, one-way ANOVA/Bonferroni’s multiple comparison test. NI, not infected. (C). Conventional TEM analysis of nerve terminals from PRRT2 KD hippocampal neurons revealed an increase in docked SVs and a preservation of the total SVs with respect to control neurons. Shown are representative TEM images of nerve terminals from neurons transduced with either Scr or Sh4 at 7 DIVs and fixed/processed at 14 DIVs. Scale bar, 200 μm. (D) Quantitative TEM analysis of the synaptic density from serial ultrathin sections. The volume density of symmetric and asymmetric synapses was calculated from the 2D count of synaptic profiles in sections from Sh4- (red bars) and Scr-treated (black bars) neurons and is expressed as mean (± SEM) number of synapses per square micrometer. (E). 3D reconstructions of synaptic terminals from serial ultrathin sections confirm the increase in the number of docked SVs in low-density neuronal cultures. Shown are representative 3D reconstructions from 60-nm-thick serial sections obtained from Scr-treated (left) and PRRT2 KD (right) synapses. Total SVs and SVs physically docked at the AZ are depicted as blue and red spheres, respectively. The AZ and mitochondria are shown in yellow and green, respectively. Scale bar, 200 nm. (F and G) Morphometric analysis of three-dimensionally reconstructed synapses. PRRT2 KD synapses (red bars) displayed an increased number of AZ-docked SVs (F) and a preserved total number of SVs (G) with respect to Scr-treated synapses (black bars). (H) Spatial distribution of SVs in nerve terminals of Scr-treated (black symbols) and Sh4-treated (red symbols) neurons. The mean (± SEM) number of SVs located within successive 50-nm shells from the AZ and normalized by the total SV content of each terminal is given as mean ± SEM as a function of the distance from the AZ. (I) Morphometric analysis of SV diameter. PRRT2 KD synapses (red bars) displayed a smaller SV size with respect to Scr-treated synapses (black bars). Nerve terminal areas (0.689 ± 0.044 μm^2^ and 0.741 ± 0.035 μm^2^ for Scr- and PRRT2 sh-RNA-infected neurons, respectively) and AZ lengths (0.302 ± 0.023 μm and 0.347 ± 0.013 μm for Scr- and PRRT2 sh-RNA-infected neurons, respectively) were similar in the two experimental groups (140 and 150 synapses for Scr and PRRT2 shRNA-infected neurons, respectively, from three independent preparations). ^∗^p < 0.05, ^∗∗^p < 0.01, ^∗∗∗^p *<* 0.001, unpaired Student’s t test (E, G, and H) and Kolmogorov-Smirnov test (F). See also Figure S2.

Because PRRT2 expression paralleled synapse formation and rearrangement, we studied whether its knockdown (KD) was associated with changes in synaptic density or assembly. The effects of PRRT2 KD on synaptic connectivity in primary hippocampal neurons were analyzed by counting synapse density in mature (14 DIVs) neurons infected with either Sh4 or Sh1 at 7 DIVs (Figures 2A and 2B; Figures S2A and S2B). Synaptic boutons were unambiguously identified by counting puncta with a size of <1 μm double-positive for pairs of pre/postsynaptic proteins such as Bassoon/Homer1 (Figures 2A and 2B; Figure S2A) or synaptophysin/PSD95 (Figure S2B). Strikingly, the density of excitatory synapses along dendrites was decreased by ≈50% in neurons silenced for PRRT2 with either shRNA, an effect that was fully rescued by the expression of shRNA-resistant PRRT2 (Figure 2B; Figure S2B).

Next we ascertained whether the ultrastructure of the rarified synapses was affected by PRRT2 downregulation (Figures 2C–2I). Conventional transmission electron microscopy on excitatory synapses from primary neurons infected as above confirmed the decrease in synapse density in the absence of major changes in the terminal ultrastructure (Figures 2C and 2D). To analyze in greater detail the morphology of the PRRT2 KD terminals, we performed serial sectioning followed by 3D reconstruction (Figure 2E). This analysis detected a 2-fold increase in the number of docked SVs in PRRT2-silenced synapses in the context of an unaltered total number of SVs (Figures 2F and 2G). Consistent with this finding, the frequency distribution of the distance of SVs from the AZ showed an accumulation of SVs at shorter distances in PRRT2 KD terminals (Figure 2H). Interestingly, the average diameter of SVs in silenced synapses was significantly smaller than the average diameter of SVs in control terminals (Figure 2I), with an approximate one-third reduction in SV volume.

We next assessed whether these ultrastructural changes were altered by neuronal activity. To this aim, we imaged Scr- and Sh4-infected neurons immediately after a field train stimulation (300 action potentials [APs] at 10 Hz) and 20 min later (Figure S2C). Immediately after the stimulation, the density of SVs was found to be decreased to a similar extent in both Scr- and Sh4-treated synapses, but, in PRRT2 KD synapses, recovery was delayed. Interestingly, the increase in the density of docked SVs observed at rest in PRRT2 KD neurons was not evident immediately after the stimulation but reappeared after 20 min of recovery, suggesting a possible role of PRRT2 as a negative regulator of SV docking at rest.

### Knockdown of PRRT2 in Low-Density Primary Neurons Affects SV Recycling without Modifying the Size of the Readily Releasable Pool

**Figure 3:**
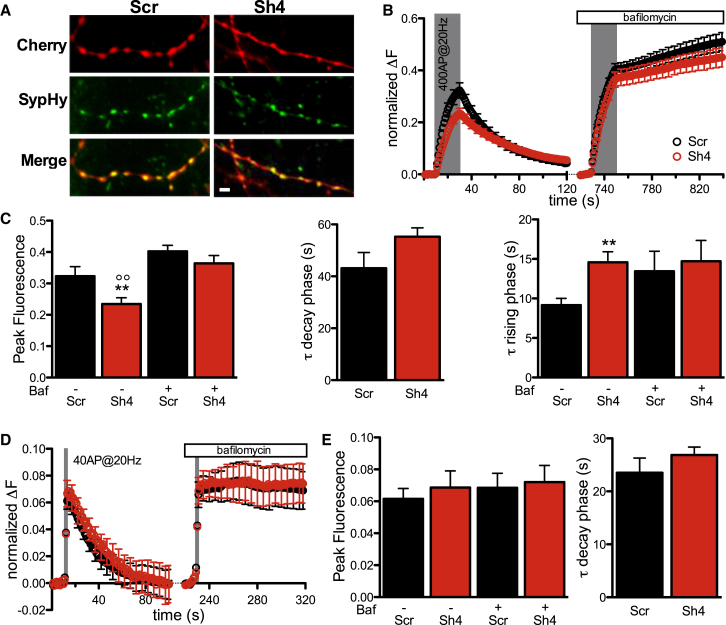
PRRT2 Knockdown Slows Down SV Cycling in Response to Sustained High-Frequency Stimulation without Altering Exocytosis (A). Representative experimental field showing low-density hippocampal neurons co-expressing the reporter of SV exo-endocytosis synaptophysin-pHluorin (Syphy) and either Sh4 or Scr (mCherry). The merged images show the colocalization of the two markers at synaptic puncta. Scale bar, 10 μm. (B). Ensemble averaged traces of SypHy fluorescence from PRRT2 KD synapses (Sh4, red trace) and control synapses (Scr, black trace) recorded in response to electrical field stimulations at 20 Hz for 20 s (shaded area) in the absence or presence of bafilomycin (1 μM). Data are normalized to the maximum fluorescence intensity reached under NH_4_Cl perfusion (normalized ΔF). (C) Left: quantitative evaluation of the SV pool released during the stimulation and plotted as peak fluorescence reached at the end of the stimulation in the absence (left) or presence (right) of bafilomycin (Baf). Center: quantitative evaluation of the kinetics of SV endocytosis (τ decay phase) by single exponential fitting of the post-stimulus curves for Sh4-treated (red) and Scr-treated (black) synapses. Right: kinetics of SV release under stimulation at 20 Hz (left) and relative time constant (τ) of the rising phase in the absence or presence of bafilomycin determined by exponential fitting of individual experiments. The rate of SV release was greatly reduced in PRRT2 KD synapses (Sh4, red trace) compared with control synapses (Scr, black trace), but the rate of pure exocytosis determined by blocking reacidification with bafilomycin was not significantly altered by PRRT2 KD. Data are expressed as mean ± SEM (shown every five time points in B) from 11 (Sh4, 340 synapses) and 13 (Scr, 380 synapses) experiments from three different preparations. ^∗∗^p < 0.01 versus Scr; °°p < 0.01 versus Sh4+Baf; one-way ANOVA/Bonferroni’s or Kruskal-Wallis/Dunn’s tests. (D and E) Evaluation of the readily releasable pool in PRRT2-silenced neurons. (D) Ensemble-averaged SypHy traces from PRRT2 KD synapses (Sh4, red trace) and control synapses (Scr, black trace) recorded in response to electrical field stimulations with 40 APs at 20 Hz (shaded area) in the absence or presence of bafilomycin. Fluorescence values were normalized to the maximum fluorescence intensity reached under NH_4_Cl perfusion (normalized ΔF). (E) Left: peak fluorescence at the end of the stimulus recorded in the absence or presence of bafilomycin. Right: time constant of endocytosis (τ decay phase) evaluated by fitting the fluorescence decay after stimulation by a single exponential function for Sh4-treated (red) and Scr-treated (black) synapses. Data are expressed as mean ± SEM (shown every five time points in D) from nine (Sh4, 260 synapses) and ten (Scr, 240 synapses) experiments from three different preparations. See also Figure S3.

To couple morphological with functional analysis of SV trafficking and monitor the SV exo-endocytotic cycle at single synapses, we employed synaptophysin-pHluorin (SypHy), a pH-sensitive fluorescent sensor exquisitely designed to monitor the SV exo-endocytosis cycle at single synapses (Figure S3A; Miesenböck et al., 1998, Kavalali and Jorgensen, 2014). SypHy was co-expressed in neurons transduced with either the Scr or Sh4 variant expressing mCherry as a spectrally non-overlapping reporter. To evaluate the effect of PRRT2 KD on SV recycling evoked by electrical activity, field stimulation protocols triggering APs were applied (400 APs at 20 Hz) that allowed us to estimate the extent and kinetics of exocytosis and endocytosis (Verstegen et al., 2014). Experiments were performed at 17 DIVs (5 days after infection), and mCherry/SypHy double-positive puncta on axonal processes were selected for analysis (Figure 3A). We found that the SypHy fluorescence increase evoked by tetanic stimulation was significantly lower in PRRT2-silenced boutons in the absence of noticeable effects on the kinetics of “post-stimulus” endocytosis (Figures 3B and 3C). When the kinetics of release during stimulation were evaluated and fitted according to a mono-exponential model, a 2-fold increase in the time constant was observed in PRRT2-silenced neurons (Figure 3C). To exclude interference from endocytosis and vesicle re-acidification, we repeated the analysis in the presence of the proton pump inhibitor bafilomycin, which allows extracting the contribution of exocytosis to the rising phase of the fluorescence response to stimulation (Figure S3A). Interestingly, the significant decrease in the fluorescence peak and slowdown of the release kinetics were virtually abolished, indicating that the extent and dynamics of exocytosis, as measured by the SypHy assay, were not markedly altered by PRRT2 silencing (Figure 3C). This also suggests that PRRT2 silencing may affect during-stimulus endocytosis and/or SV re-acidification.

To clarify whether PRRT2-silenced synapses are impaired in the priming/fusion steps of release, we stimulated neurons with 40 APs at 20 Hz to measure the readily releasable pool (RRP) of SVs and assess whether the increased docked SVs observed by electron microscopy at rest were functional. We found that both the fluorescence peak at the end of the 2-s stimulation (representing the RRP size) and the kinetics of fluorescence return to basal levels at the end of the stimulation (describing the post-stimulus endocytosis) were virtually unaffected by PRRT2 silencing, indicating that the priming process produces a normal pool of releasable SVs (Figures 3D and 3E).

To confirm the abovementioned data, we used an alternative, spectrally distinct reporter of exo-endocytosis, Synaptobrevin2-Orange (Syb2O, a pH-sensitive pHluorin based on the SV protein VAMP2/synaptobrevin; Ramirez et al., 2012) co-expressed with the green tGFP reporter version of either Sh4- or Sh1-expressing vectors. The effects of PRRT2 silencing observed with the SypHy assay were fully reproduced using the Syb2O reporter and an alternative shRNA to knockdown PRRT2 (Figure S3B–S3E).

### PRRT2 Silencing Reversibly Knocks Down Spontaneous and Evoked Synaptic Transmission in Autaptic Neurons

To analyze how PRRT2 KD affects the function and strength of excitatory and inhibitory synapses and avoid the confounding effects of non-transduced neurons, we used autaptic hippocampal neurons. We first checked whether autaptic neurons exhibited the same morphological and ultrastructural changes observed in low-density cultures. VGLUT1-immunoreactive puncta representing autapses were significantly less numerous in tGFP-positive PRRT2-silenced autaptic neurons, confirming the results described in low-density cultures (Figures S4A and S4B). Except for a decrease in total SVs (that was not apparent in low-density neurons), silenced autaptic neurons exhibited a phenotype identical to low-density neurons, with a significant increase in the density of SVs docked at the AZ (Figures S4C and S4D), a shift in the frequency distribution of the distance of SVs from the AZ with accumulation of SVs at shorter distances (Figure S4E), and a decrease in SV diameter (Figure S4F).

**Figure 4:**
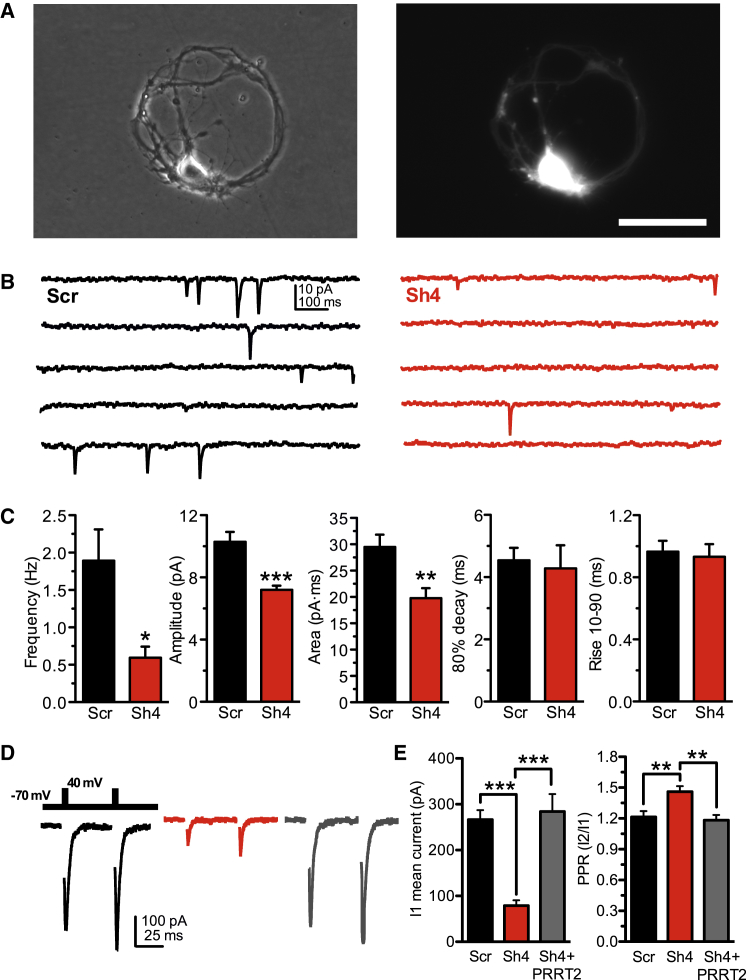
PRRT2 Knockdown Dramatically Decreases Spontaneous and Evoked Synaptic Transmission in Hippocampal Autaptic Neurons (A) Phase-contrast micrographs of a typical hippocampal autaptic neuron (left). The positivity of the same cell to infection with the Sh4 construct was probed by fluorescence imaging of the tGFP reporter (right). Scale bar, 100 μm. (B) Representative recording traces of mEPSCs from PRRT2-KD synapses (Sh4, red traces) and control synapses (Scr, black trace). (C) Analysis of mEPSCs. From left to right, shown are mean ± SEM frequency, amplitude, charge, 80% decay time, and 10%–90% rise time of mEPSCs calculated for PRRT2 KD (n = 11, red bars) and control (n = 15, black bars) neurons. All values were obtained from 100–1000 events recorded from each cell in 5-min recordings. (D) Representative eEPSCs recorded in excitatory autaptic neurons infected with Scr (black trace/bar, n = 39), PRRT2-Sh4 (red trace/bar, n = 32), or PRRT2-Sh4 + Sh-resistant PRRT2 (gray trace/bar, n = 21). eEPSCs were elicited by clamping the cell under study at –70 mV and stimulating it with two voltage steps to +40 mV lasting 0.5 ms at an inter-stimulus interval of 50 ms. The paired-pulse stimulation was applied every 10 s (inset). (E) Decrease of eEPSC amplitude and increase of PPR by PRRT2 KD and rescue of the PRRT2 KD phenotype by expression of Sh-resistant PRRT2. Shown is the quantitative analysis of the eEPSC amplitude evoked by the first pulse (I1, left) and PPR (I2/I1, right) recorded in excitatory autaptic neurons treated as described in (D). A complete rescue of the EPSC amplitude and PPR was observed in silenced neurons expressing Sh-resistant PRRT2. In all graphed currents, stimulation artifacts were blanked for clarity. Data are expressed as means ± SEM from the indicated numbers of cells recorded from at least three independent cell culture preparations. ^∗^p < 0.05, ^∗∗^p < 0.01, ^∗∗∗^p < 0.001, unpaired Student’s t test or Mann-Whitney’s U test (C); Kruskal-Wallis/Dunn’s multiple comparison test (E). See also Figures S4 and S5.

Having confirmed the morphological effects of PRRT2 silencing, we proceeded to analyze spontaneous and evoked synaptic transmission in transduced autaptic neurons identified based on tGFP/mCherry fluorescence (Figure 4A). Miniature excitatory postsynaptic currents (mEPSCs) were continuously recorded at the soma of voltage-clamped neurons held at –70 mV (V_h_) in the presence of tetrodotoxin (TTX) to block spontaneous APs (Figure 4B; Chiappalone et al., 2009). Strikingly, the mEPSC frequency fell 3- to 4-fold in PRRT2-silenced cultures (Figure 4C), an extent much larger than expected from the decrease in synapse density observed in PRRT2-silenced autaptic excitatory neurons. Moreover, the mEPSC amplitude, reflecting quantal size, was also decreased by approximately 30% in the absence of significant changes in the current waveform, as indicated by the analysis of decay and rise times (Figure 4C). A reduction of approximately the same extent was also observed in the mEPSC area (unitary charge). Such a change may be attributable to the corresponding reduction in the average SV volume, although concomitant postsynaptic effects cannot be excluded.

For the analysis of evoked transmission, transduced autaptic neurons, identified as excitatory or inhibitory based on the kinetics of postsynaptic currents (PSCs) and sensitivity to specific blockers of AMPA or γ-aminobutyric acid receptor type A (GABA_A_) receptors, were stimulated with paired stimuli at an interpulse interval of 50 ms to evaluate the paired-pulse ratio (PPR), an indirect measure of release probability (Fioravante and Regehr, 2011). Strikingly, the vast majority of excitatory neurons knocked down for PRRT2 by either Sh4 or Sh1 and analyzed over time in culture displayed a dramatic decrease of EPSC amplitude in response to single stimuli with a strong increase in paired-pulse facilitation (Figures 4D and 4E; Figures S5A–S5D), suggesting a presynaptic defect affecting release probability. The effect of PRRT2 silencing was reversible; co-infection of the neurons with Sh-resistant PRRT2 was able to achieve a virtually complete return of current amplitude and PPR to the levels of Scr-infected neurons (Figures 4D and 4E). The magnitude of the synaptic impairment increased with development, suggesting an absent or delayed maturation of the release machinery in PRRT2-silenced neurons (Figure S5B).

A closely similar phenotype was observed in PRRT2-silenced inhibitory neurons, which represent 7%–10% of the total autaptic neuronal population under our culture conditions (Figure S5E). The impairment of the evoked inhibitory current appeared to be even more dramatic than that of excitatory neurons recorded in the same plates (inset in Figure S5F). In analogy to what was observed for excitatory transmission, the strong impairment in inhibitory transmission was associated with an increase in PPR, which made paired-pulse depression milder (Figure S5G). The results suggest that PRRT2 downregulation affects the Ca^2+^ coupling between action potential and exocytosis by decreasing the probability of release (Pr), thereby rendering facilitation more intense in excitatory synapses or depression milder in inhibitory synapses.

### PRRT2 Knockdown Decreases the Probability of Synchronous Release and Greatly Increases the Asynchronous/Synchronous Release Ratio

**Figure 5:**
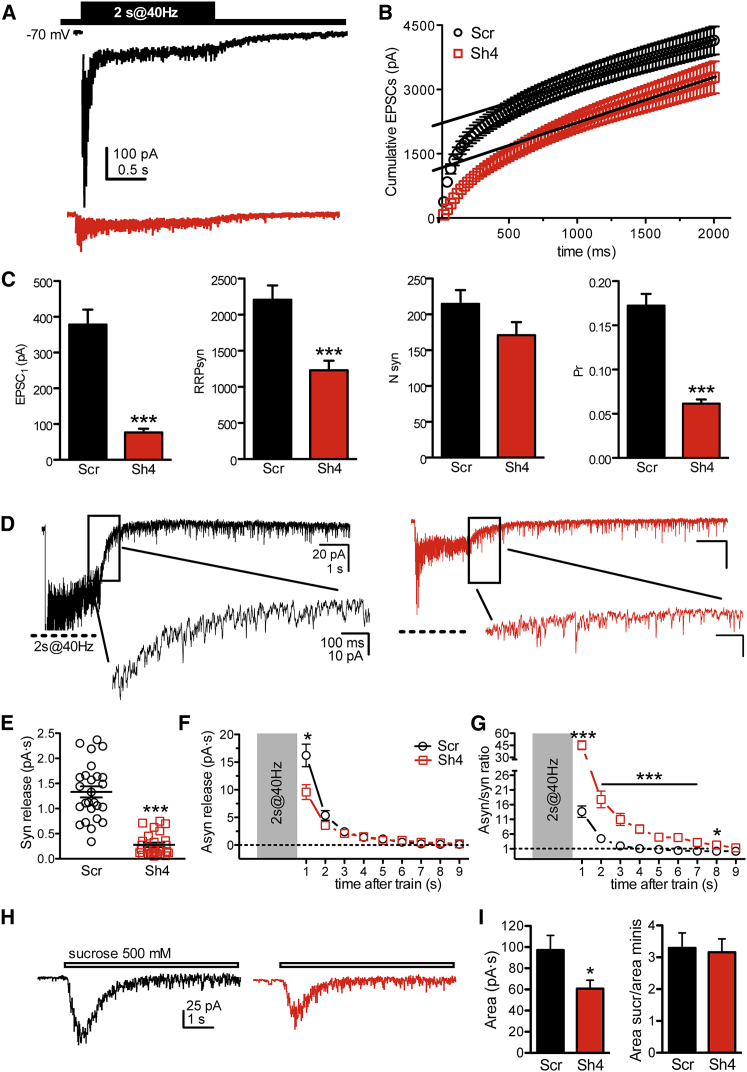
PRRT2 Knockdown Strongly Decreases Synchronous but Not Asynchronous Release in Excitatory Autapses (A) Representative recordings of eEPSCs evoked by high-frequency stimulation (train of 80 stimuli at 40 Hz, inset) in autaptic hippocampal neurons infected with Scr (black trace) or PRRT2-Sh4 (red trace). Stimulation artifacts were blanked for clarity. (B) Profiles of the mean cumulative amplitude of eEPSCs for neurons infected with Scr (black trace, n = 38) or PRRT2-Sh4 (red trace, n = 34) constructs. Data points in the 1- to 2-s range were fitted by a linear regression and back-extrapolated to time 0 (solid lines) to estimate the RRP_syn_. (C) Results of the quantal analysis. From left to right, shown are the mean ± SEM amplitude of the first eEPSC, RRP_syn_ size, number of RRP_syn_ quanta, and initial probability of release (Pr) estimated in neurons infected with Scr (n = 38, black bars) or PRRT2-Sh4 (n = 34, red bars). (D) Representative traces showing asynchronous release evoked by a tetanic stimulation of 2 s at 40 Hz in autaptic neurons transduced with either Scr (black traces) or PRRT2-Sh4 (red traces). Traces in the insets are shown in an expanded timescale. (E–G) Comparative analysis of synchronous and asynchronous release. (E) Individual values and mean (± SEM) of the synchronous charge released from Scr-infected (black, n = 26) and Sh4-infected (red, n = 26) neurons stimulated by one AP 10 s before the train. Synchronous charge was estimated by measuring the area of the eEPSC in a time window of 5 ms following its activation. (F) Time course of asynchronous release calculated by measuring the area of the spontaneous EPSCs evoked by tetanic stimulation. The area was calculated in nine time windows, each lasting 1 s. (G) Time course of the synchronous/asynchronous ratio calculated for the nine time windows from the data shown in (C) and (B), respectively. (H and I) Estimation of RRP_total_ by hypertonic stimulation of autaptic neurons. (H) Representative EPSC responses activated by 6 s of hypertonic stimulation (horizontal bar) recorded in autaptic neurons transduced with either scrambled shRNA (black) or PRRT2-Sh4 (red). (I) Mean values (± SEM) of the RRP_total_ charge transfer obtained by hypertonic stimulation and number of RRP_total_ quanta obtained in either Scr (black, n = 18) or PRRT2-Sh4 (red, n = 15) neurons by dividing the RRP_total_ charge by the unitary mEPSC charge. Data are shown as means ± SEM. ^∗^p < 0.05, ^∗∗^p < 0.01, ^∗∗∗^p < 0.001, unpaired Student’s t test or Mann-Whitney *U* test.

To identify the quantal parameters of release affected by PRRT2 silencing, we performed cumulative evoked EPSC (eEPSC) amplitude analysis in control and silenced autaptic excitatory neurons. When neurons were challenged with a train of 2 s at 40 Hz, a significant depression of eEPSCs became apparent during the train in both control and silenced neurons, irrespective of the amplitude of the first current in the train (Figure 5A). Accordingly, the cumulative profile showed a rapid rise followed by a slower linear increase, reflecting the equilibrium between the depletion and constant replenishment of the RRP (Figure 5B).

Interestingly, the 4- to 5-fold reduction of the current amplitude in silenced neurons was contributed by a sharp (about −65%) decrease in the initial Pr together with a significant decrease of the RRP_syn_ total current. However, when the readily releasable pool current of synchronous release (RRP_syn_) was normalized by the unitary current (Q_av_, corresponding to the average amplitude of autaptic mEPSCs), the number of readily releasable SVs (N_syn_) was not significantly different between control and PRRT2-silenced neurons (Figure 5C), suggesting, in agreement with the results of the SypHy assay, a normal priming process generating the RRP in PRRT2 KD neurons. Therefore, the strong impairment in evoked synchronous release is likely to involve changes in the Ca^2+^ dependence of release that can occur at the level of either Ca^2+^ entry or Ca^2+^ sensing.

Because asynchronous release has a distinct Ca^2+^ sensitivity, we measured both the synchronous and the delayed asynchronous release as the transferred charge in response to trains of 2 s at 40 Hz (Medrihan et al., 2013; Figure 5D). Although the synchronous charge, measured as the response to a single AP 10 s before the train, was dramatically decreased in PRRT2-silenced neurons (Figure 5E), both control and silenced autapses showed a robust asynchronous release after the train that slowly returned to baseline within 10 s (Figures 5F and 5G). In PRRT2-silenced autapses, asynchronous release was significantly decreased only immediately after the end of the train, but thereafter it was indistinguishable from that of control autapses (Figure 5F). When the asynchronous/synchronous charge ratio was calculated (Figure 5G), silenced neurons had a percent asynchronous versus synchronous release that was much larger than that of control neurons. This is compatible with the normal released SV pool observed with the SypHy assay (as shown previously shown for Syt1 knockout neurons; Nishiki and Augustine, 2004) and further suggests that the main impairment in the absence of PRRT2 resides in Ca^2+^ sensing commanding fast, synchronous release and not in the subsequent asynchronous SV fusion process.

Given the sharp decrease in synchronous release and the substantial preservation of asynchronous release, we assessed the RRP size of the total release (RRP_total_) with a fully independent procedure; i.e., by exposing synapses to hypertonic sucrose stimulation (Figure 5H). Consistent with the data reported above, during the transient part of the high sucrose-induced eEPSC, a significant decrease of the charge transfer, a reliable measure of RRP_total_, was observed in PRRT2-silenced neurons (Figure 5I). However, when RRP_total_ was normalized by the unitary SV charge, the resulting quantal content of RRP_total_ was comparable between control and PRRT2-silenced neurons (Figure 5I). The substantial preservation of the total pool of readily releasable SVs indicates that the same total SV pool is shared by synchronous and asynchronous release and further suggests that SV priming is not affected by PRRT2 silencing.

### PRRT2 Knockdown Decreases the Ca^2+^ Sensitivity of Synchronous Release and Alters Short-Term Plasticity

**Figure 6:**
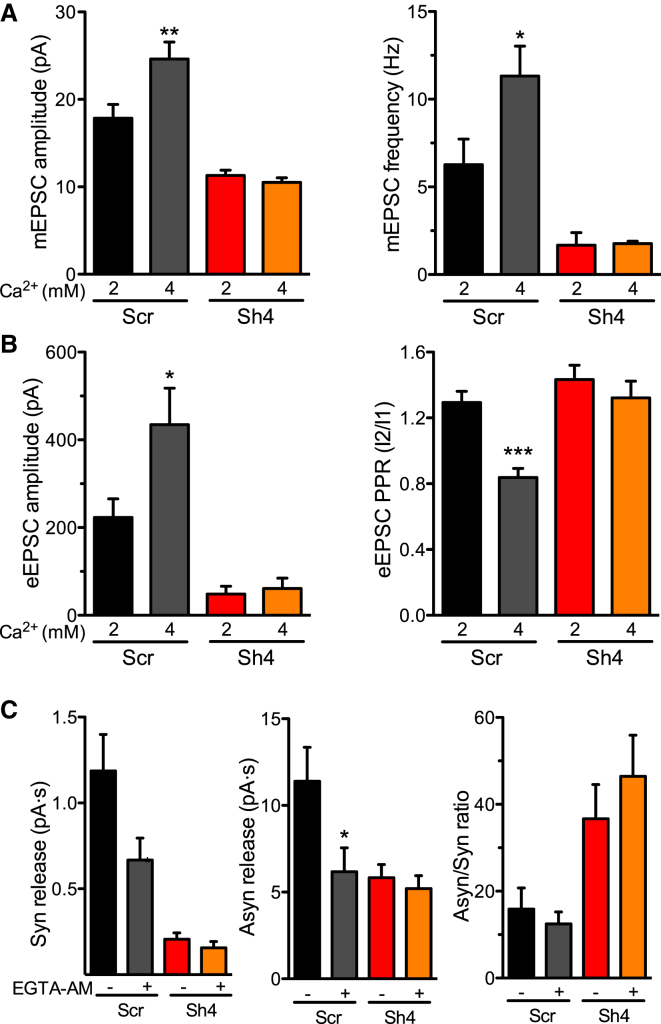
The Ca^2+^ Sensitivity of Spontaneous and Synchronous Release Is Decreased by PRRT2 Knockdown (A) Amplitude (left) and frequency (right) of mEPSCs recorded in neurons transduced with Scr (black/gray bars, n = 9/7) and Sh4 (red/orange bars, n = 10/8) as a function of the extracellular Ca^2+^ concentration (2 mM Ca^2+^, black/red bars; 4 mM Ca^2+^, gray/orange bars). (B) eEPSC amplitude evoked by the first pulse (I1, left) and PPR (I2/I1, right) recorded in neurons transduced with Scr (black/gray bars, n = 7) or PRRT2-Sh4 (red/orange bars, n = 6). The graph bars represent the mean of the EPSC amplitude and PPR recorded in individual cells before (black/red bars) and after (gray/orange bars) the increase of the extracellular Ca^2+^ concentration to 4 mM. (C) Effects of EGTA-AM on synchronous and asynchronous release. Left: synchronous charge released from Scr-treated (black/gray bars, n = 12) and Sh4-treated (red/orange bars, n = 11) neurons stimulated by one AP 10 s before the train. Synchronous charge was estimated by measuring the area of the eEPSC in a time window of 5 ms following its activation. Center: asynchronous charge induced by a tetanic stimulation of 2 s at 40 Hz and calculated by measuring the area of the spontaneous EPSCs in the time window of 1 s after the end of the train. Right: synchronous/asynchronous ratio calculated for the nine time windows from the data shown in (C) and (B), respectively. Data are means ± SEM. ^∗^p < 0.05, ^∗∗^p < 0.01, ^∗∗∗^p < 0.001, one-way ANOVA/Bonferroni’s multiple comparison test. See also Figure S6.

Given the sharp impairment in spontaneous and evoked release, we investigated the Ca^2+^ sensitivity of release by increasing the extracellular Ca^2+^ concentration or by challenging synchronous and asynchronous release with the cell-permeable Ca^2+^ chelator EGTA-acetoxymethyl (EGTA-AM). In Scr-treated neurons, the increase in external Ca^2+^ from 2 to 4 mM enhanced the frequency and amplitude of mEPSCs (Figure 6A) as well as the amplitude of eEPSCs while decreasing PPR (Figure 6B), consistent with a heightened release probability. Strikingly, PRRT2-silenced neurons were totally insensitive to the increase in extracellular Ca^2+^ as far as both spontaneous and evoked release were concerned.

Known as a slow chelator, EGTA should be more active in inhibiting delayed asynchronous release rather than fast, synchronous release triggered by Ca^2+^ increases in the nanodomains adjacent to Ca^2+^ channels. However, in control hippocampal autapses, EGTA-AM application significantly reduced both synchronous and asynchronous release to approximately the same extent, indicating that fast release occurs within microdomains generated by the spatial overlap of local Ca^2+^ influxes through multiple adjacent Ca^2+^ channels (Wang and Augustine, 2014). Interestingly, the effect of EGTA was fully occluded by PRRT2 KD, consistent with an effect on the Ca^2+^ dependency of release (Figure 6C).

An alternative way to assess Ca^2+^ sensitivity of release is to investigate facilitation and potentiation, which are short-term plasticity phenomena depending on an activity-dependent Ca^2+^ build-up in the nerve terminal (Figure S6; Fioravante and Regehr, 2011). Excitatory autaptic neurons were stimulated with 2-s trains at frequencies ranging from 10–40 Hz, and the development of facilitation/depression was analyzed over time. PRRT2-silenced neurons were characterized by a massive and intense facilitation whose peak was significantly delayed with respect to that of control neurons (Figures S6A and S6C). Interestingly, the amplitude of the last EPSC of the train became indistinguishable from the last EPSC of control neurons that was largely depressed at 40 Hz of stimulation (Figure S6B). When the EPSC amplitude during the train was normalized with the amplitude of the first EPSC, PRRT2-silenced neurons exhibited an intense and prolonged facilitation without experiencing any depression during 10- or 20-Hz trains (Figure S6C). Although the pronounced facilitation and the increased PPR are the likely consequences of the low initial Pr, the delay in the facilitation peak further suggests an impaired Ca^2+^-sensing ability. Post-tetanic potentiation (PTP) is a form of short-term synaptic plasticity contributed by increases in both RRP and Pr induced by the train stimulation (Fioravante and Regehr, 2011, Valente et al., 2012). A short high-frequency stimulation (HFS) (2 s at 40 Hz; Figure S6D) was used to evoke PTP, and a single stimulus applied every 10 s was used to monitor the plastic changes in eEPSC amplitude. PRRT2-silenced neurons expressed an approximately 30% smaller potentiation than Scr-treated control neurons (Figures S6E and S6F), consistent with an inability of PRRT2-silenced neurons to increase the Pr because of an impairment in the Ca^2+^ sensing process.

### PRRT2 Interacts with SNARE Proteins and Synaptotagmins 1 and 2

**Figure 7:**
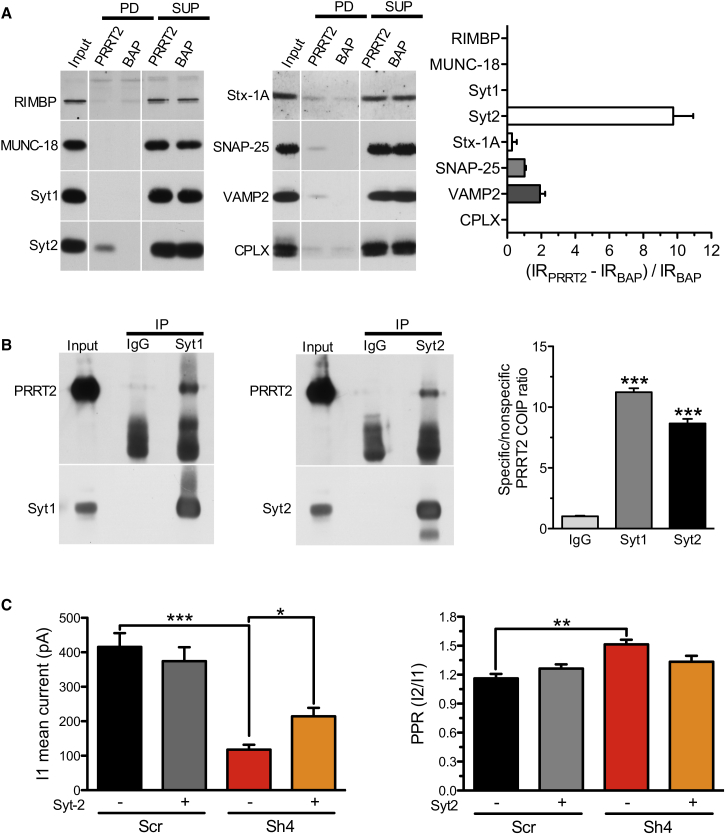
PRRT2 Interacts with the SNARE Complex Proteins and the Fast Ca^2+^ Sensors Synaptotagmin 1 and 2 (A) Pull-down assays with FLAG-tagged PRRT2. Left: FLAG-tagged PRRT2 (PRRT2) and bacterial alkaline phosphatase (BAP) vectors were expressed in HEK293T cells, purified by anti-FLAG M2 affinity gel, and incubated with synaptosome lysates. After pull-down (PD), pellets were solubilized and subjected to a western blotting assay together with aliquots of the starting material (input) and of the supernatants (SUP) using antibodies for a variety of potential presynaptic interactors of PRRT2, as shown. Vertical white lines in the blot indicate that the lanes were on the same gel but have been repositioned in the figure. Synaptosomal lysates incubated with FLAG-PRRT2 showed specific immunoreactivity for the SNARE complex proteins SNAP-25 and VAMP2 and for Syt2, which was not detected in FLAG-BAP precipitates. RIMBP, Rim binding protein; Syt, synaptotagmin; Stx-1A, syntaxin-1A, CPLX, complexins 1/2. Right: Immunoblots were quantified by densitometric analysis of the fluorograms obtained in the linear range of the emulsion response. The percent increase in the specific pull-down of the immunoreactivity (IR) by FLAG-PRRT2 with respect to the non-specific IR pulled down by the FLAG-BAP control ((IR_PRRT2_ – IR_BAP_) / IR_BAP_) was calculated and is shown for each potential interactor as mean ± SEM (n = 3–4 independent PRRT2 preparations and subcellular fractionations). (B) Co-immunoprecipitation of PRRT2 with Syt1 and Syt2. Detergent extracts of mouse brain were immunoprecipitated (IP) with monoclonal antibodies (mAbs) specific for Syt1 and Syt2 or with the respective control mouse immunoglobulin Gs (IgGs) as indicated. After electrophoretic separation of the immunocomplexes and western blotting, membranes were probed with anti-Syt1/anti-Syt2 antibodies to test the efficiency of the immunoprecipitation as well as with polyclonal anti-PRRT2 antibodies. Left: a representative immunoblot is shown. Right: quantification of the PRRT2 immunoreactive signal in the immunoprecipitated samples, normalized to the binding to the mouse IgG control (means ± SEM, n = 3 independent experiments). Input, 10 μg total extract. (C) Overexpression of Syt2 partially rescues the impairment in synchronous release of PRRT2 KD neurons. Autaptic hippocampal neurons were infected at 7 DIVs with Scr (n = 31), Sh4/mCherry (Sh4, n = 30), Syt2/GFP (Syt2, n = 28), or Sh4+Syt2 (n = 27) and recorded at 14 DIVs. The histograms show the means ± SEM of the eEPSC amplitude evoked by the first pulse (I1, left) and of the PPR (I2/I1, right). ^∗^p < 0.05, ^∗∗^p < 0.01; ^∗∗∗^p < 0.001; Kruskal-Wallis/Dunn’s multiple comparison test (I1); one-way ANOVA/Bonferroni’s multiple comparison test (PPR). See also Figure S7.

The physiological phenotype of PRRT2 silencing prompted the search for possible presynaptic PRRT2 interactors implicated in the post-docking events of release. We focused on presynaptic proteins that, when inactivated in knockout (KO) mice, cause a strong impairment in evoked release, including the SNARE proteins VAMP2, SNAP-25, and syntaxin 1, the fast Ca^2+^ sensors synaptotagmins (Syt) 1/2, Rab3 interacting molecule (RIM) binding protein, complexins 1/2, and Munc-18 (Südhof, 2013). FLAG-tagged PRRT2 or a FLAG-tagged control purified in eukaryotic cells was used to pull down PRRT2 interactors from synaptosomal extracts of the mouse brain. A significant pull-down of Syt2 as well as a somewhat weaker interaction with SNAP-25 and its SNARE partner VAMP2 were observed in the absence of significant interactions with the other proteins tested (Figure 7A). Although the interaction of PRRT2 with SNAP-25 has been reported previously (Stelzl et al., 2005, Lee et al., 2012, Li et al., 2015), the interaction with Syt2, a fast Ca^2+^ sensor highly homologous to Syt1 (Südhof, 2013), is of great interest in light of the physiological phenotype associated with PRRT2 silencing. Under our conditions, Syt2 was expressed by both low-density and autaptic hippocampal neurons, although to a much lesser extent and with a more restricted pattern than the major isoform Syt1 (Figure S7A), consistent with previous reports (Pang et al., 2006a, Fox and Sanes, 2007, Kerr et al., 2008).

In view of the high homology of Syt2 with Syt1, and to explain the wide-range synaptic phenotype of PRRT2 knockdown, we further considered the possibility of an interaction of PRRT2 with Syt1 that might have escaped the conventional pull-down assay. Co-immunoprecipitation experiments from brain extracts with anti-Syt1 and anti-Syt2 antibodies showed that PRRT2 indeed associates with both Syt1 and Syt2 (Figure 7B). Because the lack of an interactor may affect the lifetime of its partner, we investigated whether the levels of Syt1 and/or Syt2 in low-density hippocampal cultures were affected by PRRT2 silencing. Both Syt isoforms were decreased in PRRT2 KD neurons. However, although the levels of Syt2 were decreased to the same extent as those of other synaptic proteins, likely expressing the decrease in synapse density, Syt1 expression levels were significantly smaller (Figure S7B).

In support of the hypothesis that the wide-range synaptic phenotype of PRRT2 KD is attributable to the interaction with either or both Syt1/Syt2, we tried to rescue the impairment in the evoked synchronous release by overexpressing Syt2 in PRRT2 KD autaptic neurons (Figure S7C). Expression of Syt2 induced a significant rescue of PRRT2 KD-induced impairment in eEPSC amplitude, although its magnitude was smaller than the complete rescue obtained with Sh-resistant PRRT2 (Figure 7C).

## Discussion

The dominant effect of PRRT2 mutations associated with diverse paroxysmal disorders points to an important role of the protein in neuronal activity. Because most of the mutations display a loss-of-function pathogenic mechanism of action, we used an acute silencing approach through RNA interference to dissect out the physiological role of the protein at the synaptic level.

### Presynaptic Actions of PRRT2

Putative interactions with the SNARE protein SNAP-25 and the GluA1 subunit of the AMPA-type glutamate receptor complex have been suggested on the basis of proteomic studies (Stelzl et al., 2005, Schwenk et al., 2014). These interactions were subsequently demonstrated using recombinant PRRT2 (Lee et al., 2012, Li et al., 2015), opening the question of whether PRRT2 acts at the presynaptic level, postsynaptic level, or both. In this work, we provide evidence for a strong involvement of PRRT2 in presynaptic function. PRRT2 is increasingly expressed during synaptogenesis and, in mature synapses, codistributes with presynaptic markers such as synaptophysin or SNAP-25. Consistently, PRRT2 silencing in primary neurons decreases the density of synaptic connections and alters the nerve terminal ultrastructure. Stronger evidence for a presynaptic action comes from the effects of PRRT2 silencing on synaptic transmission. Indeed, in the absence of PRRT2, a downregulation of neurotransmitter release occurs with a dramatic impairment in spontaneous and synchronous release. The major traits of the PRRT2 KD phenotype (namely, the decreased frequency of mEPSCs, the impairment of evoked currents in both excitatory and inhibitory synapses, the increase in PPR, the selective drop in the initial Pr, the changes in short-term plasticity responses such as facilitation, depression, or PTP that are expressed predominantly at presynaptic level, as well as the ultrastructural changes testifying to an accumulation of SVs in the proximity of the release sites at rest) all strongly indicate that PRRT2 is an important presynaptic component of the release machinery. SypHy imaging experiments also uncovered an additional effect of PRRT2 silencing on SV recycling and/or acidification that confirms the presynaptic site of action of PRRT2.

### PRRT2 and Synapse Morphology

One major effect of acute PRRT2 silencing is a decrease in the density of synaptic connections. The decreased number of synaptic contacts is more likely attributable to a developmental effect of PRRT2 whose expression parallels the period of synaptic formation and remodeling, although plastic pruning of downregulated synapses cannot be ruled out. The decreased density of synaptic connections was not accompanied by dramatic changes in the synaptic ultrastructure, except for an increase in the density of docked SVs at rest that is relieved by activity, suggesting that PRRT2 negatively controls SV docking under resting conditions, and a smaller SV volume that parallels the decrease in quantum size observed in the analysis of miniature synaptic currents. Because a pool of PRRT2 is associated with SVs, it is tempting to speculate that it may participate in the control of the integrity, size, and curvature of these organelles. Further studies are needed to dissect the molecular mechanisms of this effect.

### PRRT2 and the Quantal Properties of Release

Quantal analysis has shown that the quantum size is decreased, the RRP of SVs is preserved, and the probability of spontaneous and evoked release is severely decreased in the absence of PRRT2. Although the decrease in quantum size is of the same magnitude as the reduction in SV volume, several considerations point to a fundamental role of PRRT2 in the machinery for synchronous release; namely, the decrease in the frequency of spontaneous release was much larger than the decrease in synapse density, the probability of evoked release was dramatically decreased, and modifications of nerve terminal Ca^2+^ levels revealed a defective Ca^2+^ secretion coupling.

These results suggest that a defect in the progression of SVs through the post-docking steps of release exists in PRRT2-silenced synapses. Three independent evaluations prove that the RRP size is preserved and that priming is not markedly altered. The effects on Pr and on the efficiency of Ca^2+^-dependent fusion would suggest that the defect of PRRT2 silencing resides in the Ca^2+^-sensing apparatus and/or in the fusion machinery. However, the persistence of asynchronous release indicates that the fusion mechanism per se is not disabled in the absence of PRRT2 and that an acute defect in coupling the fast Ca^2+^ influx to exocytosis is likely to be involved.

### PRRT2: Component of the Fusion Machinery or Ca^2+^-Sensing Device?

To test for known interactors of PRRT2, we screened an array of major presynaptic proteins whose constitutive knockout strongly impairs evoked transmission (see Südhof, 2013, for a review). Besides confirming the interaction with SNAP-25, we found that PRRT2 also associates with VAMP2 and Syt1/2.

The interactions with the SNARE proteins SNAP-25 and VAMP2 would suggest that PRRT2 acts as a catalyst in the hemifusion/fusion processes, although the relative preservation of asynchronous release argues against a major impairment in SNARE function and fusion. On the other hand, the dramatic drop in evoked synchronous release, which was insensitive to manipulations of the intraterminal Ca^2+^ levels, and the delayed peak of facilitation point to a defective excitation-secretion coupling in which either Ca^2+^ entry or Ca^2+^ sensing could be affected.

The interactions with Syt1/Syt2 open the possibility that PRRT2 participates in the regulation of the Ca^2+^-sensing apparatus for fast synchronous release. Such interactions may involve the large N-terminal region of PRRT2 that we have shown recently to have an intracellular topology (Rossi et al., 2016). Together with Syt9, Syt1 and Syt2 are the synaptotagmins that mediate fast Ca^2+^-triggered SV exocytosis (Xu et al., 2007, Pang and Südhof, 2010, Kochubey et al., 2011). Syt1 and Syt2 are highly homologous, form hetero-oligomers on the SV membrane (Osborne et al., 1999), and are thought to act in much the same way. Knockout of either Syt abolishes Ca^2+^-triggered synchronous release, and the two Syts appear to be interchangeable to some extent in rescuing their knockout phenotype (Xu et al., 2007, Nagy et al., 2006).

Deletion of Syt1 in neurons revealed that the protein is essential for mediating fast and synchronous fusion events in response to isolated APs but is not required for spontaneous fusion (that is enhanced) and slow asynchronous fusion (Geppert et al., 1994). At the mouse neuromuscular junction, where Syt1 and Syt2 are co-expressed, genetic deletion of Syt2 generates a strikingly similar phenotype to PRRT2 silencing with a dramatic reduction and slowdown of evoked release, decreased release probability, marked increase in facilitation, and asynchronous release during and after stimulus trains (Pang et al., 2006a). In the calyx of Held, where Syt2 is the only fast Ca^2+^ sensor to be expressed, Syt2 KO deletes fast release but leaves asynchronous release unaffected (Sun et al., 2007). A similar dramatic reduction and slowdown of evoked release was present in a mouse strain bearing a destabilizing Syt2 mutation (Pang et al., 2006b) and exhibiting ataxia, one of the manifestations of PRRT2 mutations in patients.

Because it is known that spontaneous, synchronous, and asynchronous release modalities rely on distinct ranges of Ca^2+^ concentrations and, potentially, on distinct Ca^2+^ sensors, it is tempting to speculate that the interaction of PRRT2 with Syt1/2 is the key to mechanistically explain the strong and selective impairment of synchronous release with PRRT2 KD. The main phenotypic difference between Syt KOs and PRRT2 KD is observed in spontaneous release, which is increased in the former and impaired in the latter because of the concomitant decrease in synapse density. Our data suggest a model whereby PRRT2, by binding to SNAP-25 and Syt1/2, would increase the affinity of the SNARE complex for the fast Ca^2+^ sensor so that, in the absence of PRRT2, less SNARE complexes bind to the sensor, resulting in a dramatic decrease of the release probability. According to this model, the interactions between PRRT2 and Syt1/2 would be necessary to boost the Ca^2+^ sensitivity of evoked fast release, whereas they would be dispensable for the Syt-mediated clamping action on spontaneous release. Finally, the similar PRRT2-silencing phenotype found in excitatory and inhibitory autapses is fully consistent with an interaction of PRRT2 with both Syt1 and Syt2, which appear to be partly segregated in excitatory and inhibitory neurons, respectively (Fox and Sanes, 2007, Kerr et al., 2008, Pang et al., 2006a).

In conclusion, we demonstrate here that PRRT2 is an important regulator of the Ca^2+^-sensing and SV fusion machinery. The results are consistent with a model in which PRRT2, by interacting with the fast Ca^2+^ sensors Syt1/2 and SNAREs, endows the SNARE complex with Ca^2+^ sensitivity. Further investigations will be needed to clarify the physiological regulation of these interactions and how their perturbation leads to brain paroxysmal activity.

## Experimental Procedures

Sprague-Dawley rats and C57BL/6J mice of either sex from Charles River Laboratories were used throughout. All experiments were carried out in accordance with the guidelines established by the European Communities Council (Directive 2010/63/EU of March 4, 2014) and were approved by the Italian Ministry of Health. The standard procedures for subcellular fractionation, western blotting, immunocytochemistry, pull-down and co-immunoprecipitation assays, real-time PCR, and cultures of low-density and autaptic hippocampal neurons are reported in detail in the Supplemental Experimental Procedures.

### Transduction of Primary Neurons with PRRT2 shRNA and/or Sh-Resistant PRRT2

shRNAs (Scr, Sh1, and Sh4; Supplemental Experimental Procedures) were inserted into the pLKO.1-CMV-TurboGFP or pLKO.1-CMV-mCherry plasmid expressing either free tGFP or mCherry for the identification of the transfected cells. The PRRT2 Sh-resistant isoforms were subcloned into lentiviral vectors 743.pCCLsin.PPT.hPGK.GFP.Wpre (a gift from Dr. L. Naldini). Lentiviral vectors encoding Sh1, Sh4, or the Scr control RNAs were used to knock down the endogenous PPRT2 protein in hippocampal neurons. Neurons were infected by the addition of lentiviral vectors into cell medium at 6 DIVs. After 24 hr, the medium was removed and replaced with fresh and conditioned medium (1:1). A multiplicity of infection (MOI) of 5 of the lentivirus was used. For rescue experiments, neurons were co-transduced with lentiviruses expressing Sh1 or Sh4 shRNA and lentiviruses expressing the Sh1- or Sh4-resistant PRRT2-Cherry (5 MOI + 5 MOI) or with Scr sh-RNA (10 MOI). Syt2 overexpression was performed by co-infection of autaptic neurons with the Sh4 shRNA vector expressing soluble mCherry and with a lentiviral vector expressing Syt2 and soluble GFP (Biomatik). All physiological experiments were conducted between 10 and 15 DIVs.

#### Live Imaging of Exo-endocytosis

To couple the synaptic ultrastructure with functional analysis of SV trafficking, we employed Syphy and Syb2O, fluorescent probes exquisitely designed to monitor the SV exo-endocytosis cycle at single synapses (Figure S3A; Miesenböck et al., 1998, Ramirez et al., 2012). Optical recordings were performed at 17 DIV (5 days after transduction), as described in detail in the Supplemental Experimental Procedures.

#### Transmission Electron Microscopy

Autaptic hippocampal neurons and low-density cultures of hippocampal neurons were infected at 7–11 DIVs with either scramble shRNA or PRRT2 shRNAs and processed for transmission electron microscopy (TEM). The detailed procedures are reported in the Supplemental Experimental Procedures.

#### Patch-Clamp Recordings

Whole-cell patch-clamp recordings were made from autaptic neurons grown on microislands, as described previously (Valente et al., 2012). The detailed procedures are reported in the Supplemental Experimental Procedures.

#### Statistical Analysis

Data were expressed as means ± SEM for number of cells (n). Normal distribution was assessed using D’Agostino-Pearson’s normality test. To compare two sample groups, Student’s t test or Mann-Whitney *U* test was used. To compare more than two sample groups, ANOVA followed by Bonferroni’s/Dunnett’s post hoc tests or Kruskal-Wallis test followed by Dunn’s post hoc test were used. Statistical analysis was carried out using OriginPro-8 (OriginLab) and Prism (GraphPad) softwares.

## Author Contributions

P.V. performed the electrophysiological experiments and analyzed the data. E.C. and M.O. performed and analyzed the ultrastructural experiments. P.R., B.S., and R.I.C. performed the cell biology studies. M.F. performed the live imaging experiments. F.O. and S.G. performed the biochemical experiments. B.S., A.M., and P.R. performed the affinity purification experiments. F.C.G. and E.M. ran the developmental studies and prepared the experimental tools. C.P. contributed to the electrophysiological experiments. A.F., P.B., and F.Z. supervised the electrophysiological and live imaging experiments, analyzed data, and contributed to writing the paper. F.B., F.V., and A.C. designed and supervised the research. F.B. and F.V. wrote the paper and supported the study.

## Supplemental Information

Document S1. Supplemental Discussion, Supplemental Experimental Procedures, and Figures S1–S7

Document S2. Article plus Supplemental Information

## References

[bib1] Chen W.J., Lin Y., Xiong Z.Q., Wei W., Ni W., Tan G.H., Guo S.L., He J., Chen Y.F., Zhang Q.J. (2011). Exome sequencing identifies truncating mutations in PRRT2 that cause paroxysmal kinesigenic dyskinesia. Nat. Genet..

[bib2] Chiappalone M., Casagrande S., Tedesco M., Valtorta F., Baldelli P., Martinoia S., Benfenati F. (2009). Opposite changes in glutamatergic and GABAergic transmission underlie the diffuse hyperexcitability of synapsin I-deficient cortical networks. Cereb. Cortex.

[bib3] Ebrahimi-Fakhari D., Saffari A., Westenberger A., Klein C. (2015). The evolving spectrum of PRRT2-associated paroxysmal diseases. Brain.

[bib4] Fioravante D., Regehr W.G. (2011). Short-term forms of presynaptic plasticity. Curr. Opin. Neurobiol..

[bib5] Fox M.A., Sanes J.R. (2007). Synaptotagmin I and II are present in distinct subsets of central synapses. J. Comp. Neurol..

[bib6] Geppert M., Goda Y., Hammer R.E., Li C., Rosahl T.W., Stevens C.F., Südhof T.C. (1994). Synaptotagmin I: a major Ca^2+^ sensor for transmitter release at a central synapse. Cell.

[bib7] Heron S.E., Grinton B.E., Kivity S., Afawi Z., Zuberi S.M., Hughes J.N., Pridmore C., Hodgson B.L., Iona X., Sadleir L.G. (2012). PRRT2 mutations cause benign familial infantile epilepsy and infantile convulsions with choreoathetosis syndrome. Am. J. Hum. Genet..

[bib8] Huttner W.B., Schiebler W., Greengard P., De Camilli P. (1983). Synapsin I (protein I), a nerve terminal-specific phosphoprotein. III. Its association with synaptic vesicles studied in a highly purified synaptic vesicle preparation. J. Cell Biol..

[bib9] Kavalali E.T., Jorgensen E.M. (2014). Visualizing presynaptic function. Nat. Neurosci..

[bib10] Kerr A.M., Reisinger E., Jonas P. (2008). Differential dependence of phasic transmitter release on synaptotagmin 1 at GABAergic and glutamatergic hippocampal synapses. Proc. Natl. Acad. Sci. USA.

[bib11] Kochubey O., Lou X., Schneggenburger R. (2011). Regulation of transmitter release by Ca(2+) and synaptotagmin: insights from a large CNS synapse. Trends Neurosci..

[bib12] Lee H.Y., Huang Y., Bruneau N., Roll P., Roberson E.D., Hermann M., Quinn E., Maas J., Edwards R., Ashizawa T. (2012). Mutations in the gene PRRT2 cause paroxysmal kinesigenic dyskinesia with infantile convulsions. Cell Rep..

[bib13] Li M., Niu F., Zhu X., Wu X., Shen N., Peng X., Liu Y. (2015). PRRT2 Mutant Leads to Dysfunction of Glutamate Signaling. Int. J. Mol. Sci..

[bib14] Medrihan L., Cesca F., Raimondi A., Lignani G., Baldelli P., Benfenati F. (2013). Synapsin II desynchronizes neurotransmitter release at inhibitory synapses by interacting with presynaptic calcium channels. Nat. Commun..

[bib15] Miesenböck G., De Angelis D.A., Rothman J.E. (1998). Visualizing secretion and synaptic transmission with pH-sensitive green fluorescent proteins. Nature.

[bib16] Nagy G., Kim J.H., Pang Z.P., Matti U., Rettig J., Südhof T.C., Sørensen J.B. (2006). Different effects on fast exocytosis induced by synaptotagmin 1 and 2 isoforms and abundance but not by phosphorylation. J. Neurosci..

[bib17] Nishiki T., Augustine G.J. (2004). Dual roles of the C2B domain of synaptotagmin I in synchronizing Ca2+-dependent neurotransmitter release. J. Neurosci..

[bib18] Osborne S.L., Herreros J., Bastiaens P.I., Schiavo G. (1999). Calcium-dependent oligomerization of synaptotagmins I and II. Synaptotagmins I and II are localized on the same synaptic vesicle and heterodimerize in the presence of calcium. J. Biol. Chem..

[bib19] Pang Z.P., Südhof T.C. (2010). Cell biology of Ca^2+^-triggered exocytosis. Curr. Opin. Cell Biol..

[bib20] Pang Z.P., Melicoff E., Padgett D., Liu Y., Teich A.F., Dickey B.F., Lin W., Adachi R., Südhof T.C. (2006). Synaptotagmin-2 is essential for survival and contributes to Ca^2+^ triggering of neurotransmitter release in central and neuromuscular synapses. J. Neurosci..

[bib21] Pang Z.P., Sun J., Rizo J., Maximov A., Südhof T.C. (2006). Genetic analysis of synaptotagmin 2 in spontaneous and Ca^2+^-triggered neurotransmitter release. EMBO J..

[bib22] Phillips G.R., Huang J.K., Wang Y., Tanaka H., Shapiro L., Zhang W., Shan W.S., Arndt K., Frank M., Gordon R.E. (2001). The presynaptic particle web: ultrastructure, composition, dissolution, and reconstitution. Neuron.

[bib23] Ramirez D.M., Khvotchev M., Trauterman B., Kavalali E.T. (2012). Vti1a identifies a vesicle pool that preferentially recycles at rest and maintains spontaneous neurotransmission. Neuron.

[bib24] Rossi P., Sterlini B., Castroflorio E., Marte A., Onofri F., Valtorta F., Maragliano L., Corradi A., Benfenati F. (2016). Novel Topology of Proline-Rich Transmembrane Protein 2 (PRRT2): Hints for an Intracellular Function at the Synapse. J. Biol. Chem..

[bib25] Schwenk J., Baehrens D., Haupt A., Bildl W., Boudkkazi S., Roeper J., Fakler B., Schulte U. (2014). Regional diversity and developmental dynamics of the AMPA-receptor proteome in the mammalian brain. Neuron.

[bib26] Stelzl U., Worm U., Lalowski M., Haenig C., Brembeck F.H., Goehler H., Stroedicke M., Zenkner M., Schoenherr A., Koeppen S. (2005). A human protein-protein interaction network: a resource for annotating the proteome. Cell.

[bib27] Südhof T.C. (2013). Neurotransmitter release: the last millisecond in the life of a synaptic vesicle. Neuron.

[bib28] Sun J., Pang Z.P., Qin D., Fahim A.T., Adachi R., Südhof T.C. (2007). A dual-Ca^2+^-sensor model for neurotransmitter release in a central synapse. Nature.

[bib29] Trabzuni D., Ryten M., Walker R., Smith C., Imran S., Ramasamy A., Weale M.E., Hardy J. (2011). Quality control parameters on a large dataset of regionally dissected human control brains for whole genome expression studies. J. Neurochem..

[bib30] Valente P., Casagrande S., Nieus T., Verstegen A.M., Valtorta F., Benfenati F., Baldelli P. (2012). Site-specific synapsin I phosphorylation participates in the expression of post-tetanic potentiation and its enhancement by BDNF. J. Neurosci..

[bib31] Verstegen A.M., Tagliatti E., Lignani G., Marte A., Stolero T., Atias M., Corradi A., Valtorta F., Gitler D., Onofri F. (2014). Phosphorylation of synapsin I by cyclin-dependent kinase-5 sets the ratio between the resting and recycling pools of synaptic vesicles at hippocampal synapses. J. Neurosci..

[bib32] Walch-Solimena C., Blasi J., Edelmann L., Chapman E.R., von Mollard G.F., Jahn R. (1995). The t-SNAREs syntaxin 1 and SNAP-25 are present on organelles that participate in synaptic vesicle recycling. J. Cell Biol..

[bib33] Wang L.Y., Augustine G.J. (2014). Presynaptic nanodomains: a tale of two synapses. Front. Cell. Neurosci..

[bib34] Wu L., Tang H.D., Huang X.J., Zheng L., Liu X.L., Wang T., Wang J.Y., Cao L., Chen S.D. (2014). PRRT2 truncated mutations lead to nonsense-mediated mRNA decay in Paroxysmal Kinesigenic Dyskinesia. Parkinsonism Relat Disord..

[bib35] Xu J., Mashimo T., Südhof T.C. (2007). Synaptotagmin-1, -2, and -9: Ca(^2+^) sensors for fast release that specify distinct presynaptic properties in subsets of neurons. Neuron.

