# Supplementary material for: PRRT2 Is a Key Component of the Ca2+-Dependent Neurotransmitter Release Machinery
Source: Cell Rep. 2016 Mar 24;15(1):117–31. doi: 10.1016/j.celrep.2016.03.005 (PMC4826441; doi:10.1016/j.celrep.2016.03.005)
Supplement: Document S1. Supplemental Discussion, Supplemental Experimental Procedures, and Figures S1–S7 [file mmc1.pdf]

Cell Reports, Volume *15*

Supplemental Information

PRRT2 Is a Key Component of the Ca^{2+}-Dependent Neurotransmitter Release Machinery

Pierluigi Valente, Enrico Castroflorio, Pia Rossi, Manuela Fadda, Bruno Sterlini, Romina Ines Cervigni, Cosimo Prestigio, Silvia Giovedì, Franco Onofri, Elisa Mura, Fabrizia C. Guarnieri, Antonella Marte, Marta Orlando, Federico Zara, Anna Fassio, Flavia Valtorta, Pietro Baldelli, Anna Corradi, and Fabio Benfenati

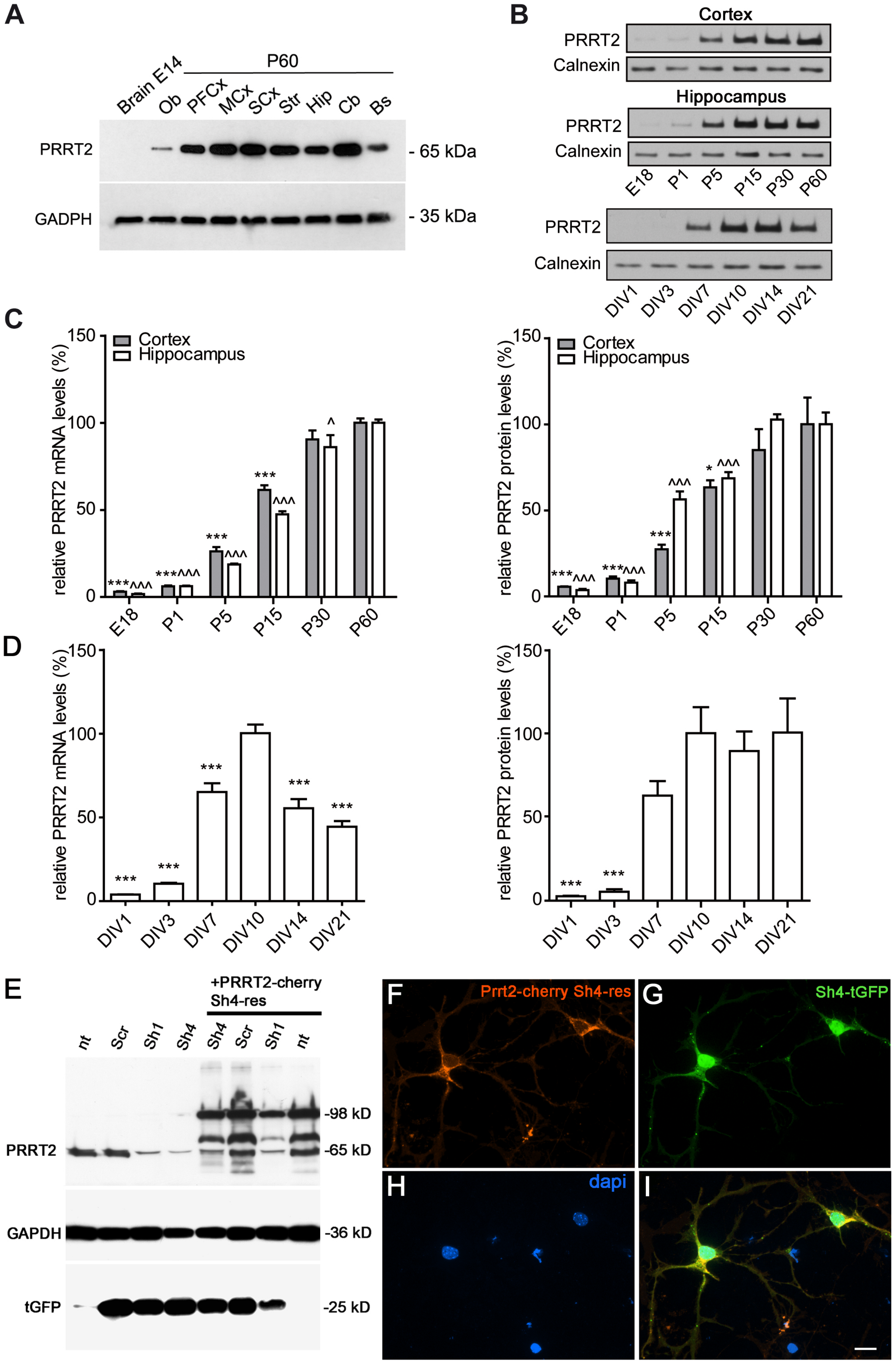
A
Brain E14
P60
Ob
PFCx
MCx
SCx
Str
Hip
Cb
Bs
PRRT2
- 65 kDa
GADPH
- 35 kDa
B
Cortex
PRRT2
Calnexin
Hippocampus
PRRT2
Calnexin
E18
P1
P5
P15
P30
P60
PRRT2
Calnexin
DIV1
DIV3
DIV7
DIV10
DIV14
DIV21
C
Cortex
Hippocampus
relative PRRT2 mRNA levels (%)
relative PRRT2 protein levels (%)
D
relative PRRT2 mRNA levels (%)
relative PRRT2 protein levels (%)
E
+PRRT2-cherry
Sh4-res
nt
Scr
Sh1
Sh4
Sh4
Scr
Sh1
nt
PRRT2
-98 kD
-65 kD
GAPDH
-36 kD
tGFP
-25 kD
F
Prrt2-cherry Sh4-res
G
Sh4-tGFP
H
dapi
I

Figure S1

Figure S1 (related to Fig. 1). Physiological expression of PRRT2 and downregulation in primary neurons by RNA interference

A. Regional expression of PRRT2 in the mouse brain. Western blot analysis of PRRT2 protein levels in mouse brain regions at embryonic (E14) and postnatal (P60) stages of development. Brain, total brain; Ob, olfactory bulb; PFCx, Prefrontal cortex; MCx, Motor cortex; SCx, Somatosensory cortex; Str, striatum, Hip, hippocampus; Cb, cerebellum, Bs, brain stem. GAPDH was used as loading control.

B-D. Temporal expression profile of PRRT2 in the developing mouse brain and in primary hippocampal neurons. **B**. Representative immunoblots of PRRT2 and calnexin expression in the cortex and hippocampus of developing mice and in primary hippocampal neurons as a function of the days in vitro (DIV). **C**. Expression profile of PRRT2 mRNA (*left*) and protein (*right*) analyzed by qRT-PCR and western blotting, respectively in the cerebral cortex (grey bars) and hippocampus (open bars) of developing mice (from embryonic day 18 to postnatal day 60). The levels of PRRT2 mRNA and protein were very low, albeit detectable, during the first stages of development (E18 and P1), increased between P5 and P30 and remained almost stable in the adulthood. Data are expressed as mean percentage intensity (± SEM) of 3 different animals with respect to P60. *$p<0.05$, ***$p<0.001$, *vs* P60 cortex; ^$p<0.05$, ^^^$p<0.001$ *vs* P60 hippocampus; one-way ANOVA followed by the Dunnett's multiple comparison test. **D**. Expression profile of PRRT2 mRNA (*left*) and protein (*right*) analyzed in primary cultures of E18 hippocampal neurons at various stages of development (from 1 to 21 DIV). PRRT2 mRNA and protein levels were barely detectable during the first stages of development (1-3 DIV), started to increase at DIV 7, peaked at DIV 10 and remained relatively high and stable thereafter. Data are expressed as mean percentage intensity ± SEM of 3 independent cultures with respect to DIV10. ***$p<0.001$ *vs* DIV10; one-way ANOVA/Dunnett's multiple comparison test.

E-I. Silencing and rescue of endogenous PRRT2 in primary neurons. **E.** Primary neurons were transduced at 6 DIV with lentiviral vectors (pLKO.1-CMV-turboGFP) coding for Sh1, Sh4 or Scr with or without co-infection with the appropriate Sh-resistant PRRT2 construct fused to mCherry, and analyzed by western blotting at 12 DIV. Endogenous PRRT2 (65 KDa apparent molecular mass) was silenced by either Sh1 or Sh4, whereas its expression was not affected by the Scr shRNA. When neurons were co-transduced with lentiviruses expressing Sh4 and the Sh4-resistant PRRT2, the endogenous PRRT2 (65 KDa) was still downregulated, while the exogenous sh-resistant PRRT2 fused to mCherry (98 KDa) was fully expressed. nt, untreated neurons. GAPDH was used as loading control, tGFP was used as ShRNA transfection reporter. **F-I.** The expression of the Sh4-resistant PRRT2, visualized by the intrinsic mCherry fluorescence (**F**), was not affected by the expression of Sh4, visualized by the green fluorescence of tGFP in the same cells (**G**). **H.** DAPI staining. **I.** Merged image of panels F, G and H. Scale bar, 20 μm.

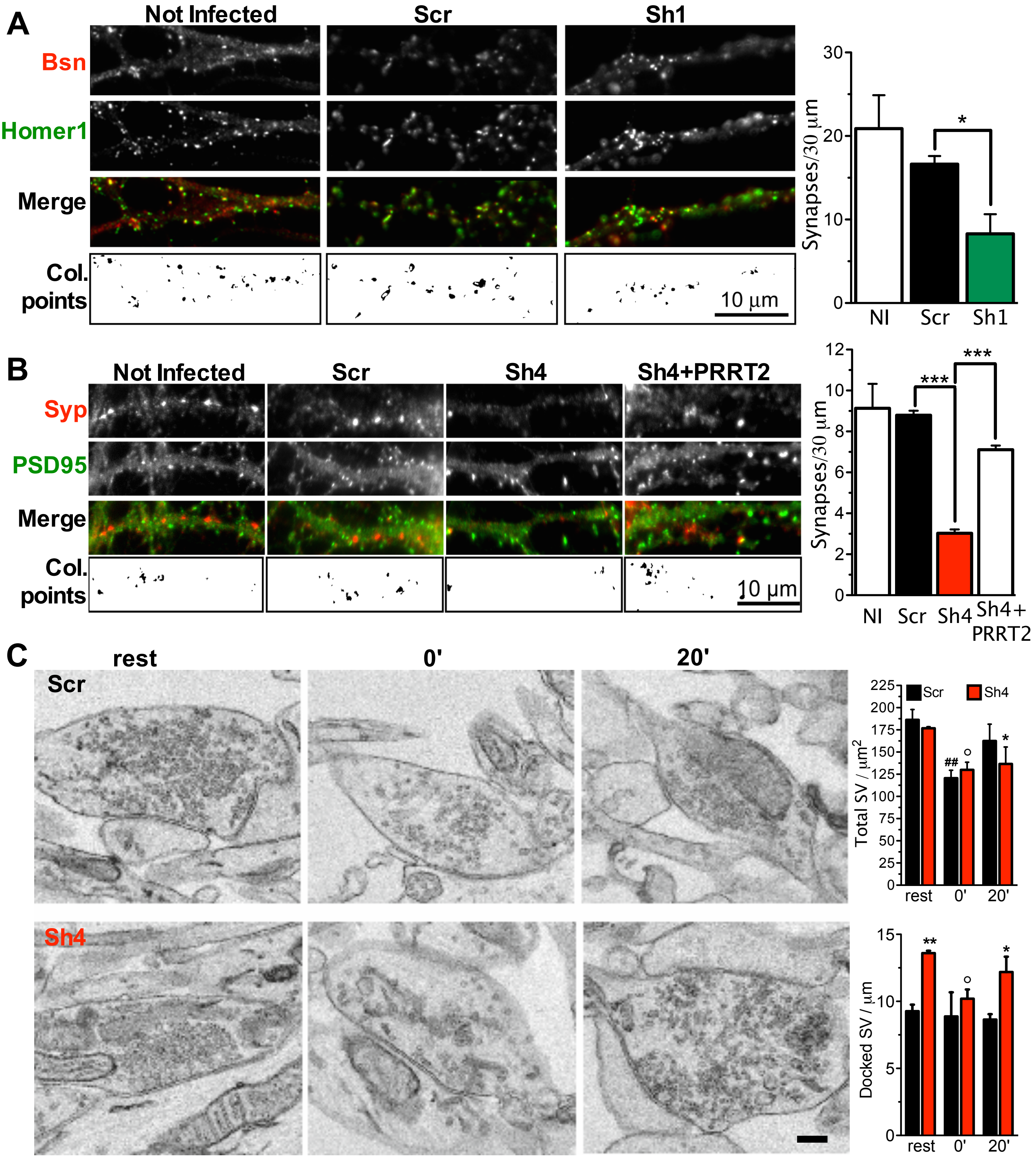
A
Not Infected
Scr
Sh1
Bsn
Homer1
Merge
Col. points
10 μm
Synapses/30 μm
NI
Scr
Sh1
B
Not Infected
Scr
Sh4
Sh4+PRRT2
Syp
PSD95
Merge
Col. points
10 μm
Synapses/30 μm
NI
Scr
Sh4
Sh4+ PRRT2
C
rest
0'
20'
Scr
Sh4
Total SV / μm2
Docked SV / μm
rest
0'
20'

Figure S2

Figure S2 (related to Fig. 2). PRRT2 knockdown decreases the total number of excitatory synapses and alters synaptic ultrastructure in an activity-dependent fashion

A. *Left:* Representative images of dendrites of hippocampal neurons infected at 7 DIV with Scr, Sh1 or left uninfected (NI) and analyzed at 14 DIV. Synaptic boutons were identified by double immunostaining for Bassoon (Bsn; red) and Homer1 (green). The colocalization panels (Col. points) highlight the double-positive puncta (black) corresponding to *bona fide* synapses. Scale bar, 10 μm. *Right:* Quantitative analysis of synaptic puncta counted on 30-μm dendrite tracts starting from the cell body. Data are means ± SEM from n=3 independent experiments, each carried out in duplicate. Five dendrites for each neuron, from at least 10 neurons for each sample were counted. *p<0.05, one-way ANOVA/Bonferroni's multiple comparison test.

B. *Left:* Representative images of dendrites of hippocampal neurons infected at 7 DIV with Scr, Sh4, Sh4 + Sh4-resistant PRRT2 or left uninfected (NI) and analyzed at 14 DIV. Synaptic boutons were identified by double immunostaining for a distinct couple of pre/post-synaptic markers, namely synaptophysin (Syp; red) and PSD95 (green). The colocalization panels (Col. points) highlight the double-positive puncta (black) corresponding to *bona fide* synapses. Scale bar, 10 μm. *Right:* Quantitative analysis of synaptic puncta counted on 30-μm dendrite tracts starting from the cell body. Data are means ± SEM from n=3 independent experiments, each carried out in duplicate. Five dendrites for each neuron, from at least 10 neurons for each sample were counted. ***p<0.001, one-way ANOVA/Bonferroni's multiple comparison test.

C. *Left:* Representative transmission electron micrographs of presynaptic terminals from low-density hippocampal neurons infected at 7 DIV with either Scr (upper row) or Sh4 (lower row). Synaptic ultrastructure was evaluated by fixing neurons under basal conditions (left column), immediately after a train of 300 APs @ 10 Hz (0'; middle column) and after 20 min of recovery (20'; right column). Scale bar, 200 nm. *Right:* Results of the ultrastructural analysis. Bar plots show the mean (± SEM) total SV density (top) and docked SV density (bottom) calculated in Scr-treated (black bars) and Sh4-treated (red bars) neurons subjected to the depression/recovery protocol. No significant changes in the nerve terminal area and in the length of the AZ were found among the experimental groups. Synaptic area (mean ± SEM, μm^2): rest, 0.59±0.03 and 0.61±0.06; 0', 0.67±0.06 and 0.56±0.02; 20', 0.64±0.07 and 0.79±0.08; for Src- and Sh4-treated neurons, respectively. AZ length (mean ± SEM, μm): rest, 0.29±0.03 and 0.30±0.03; 0', 0.34±0.04 and 0.31±0.02; 20', 0.33±0.05 and 0.33±0.02; for Src- and Sh4-treated neurons, respectively. *p<0.05, **p<0.01, across genotype; ^{o}p<0.05, ^{oo}p<0.01, within genotype; one-way ANOVA for repeated measures/Bonferroni's multiple comparison test. n = 90 images per genotype from 3 independent experiments.

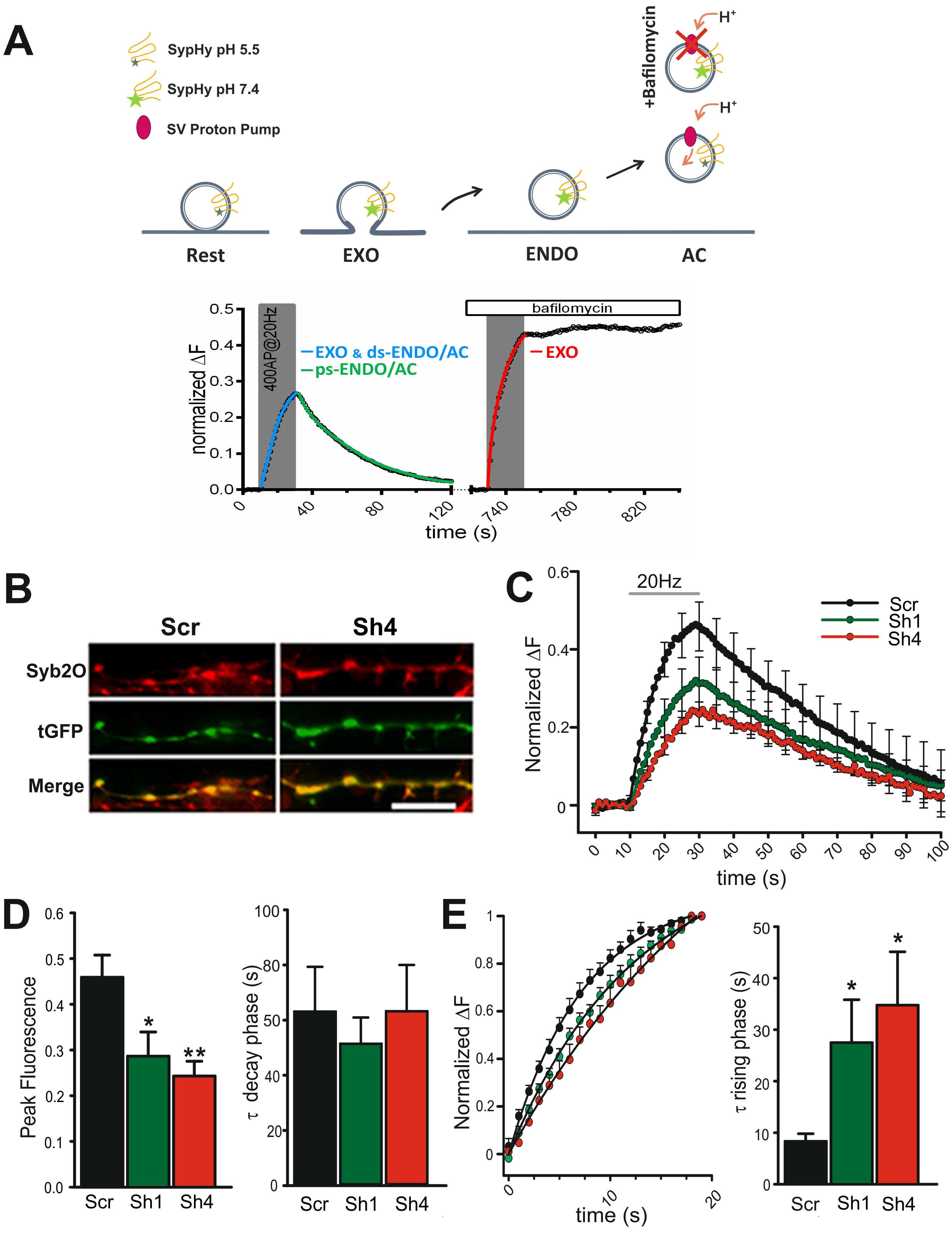
A
SypHy pH 5.5
SypHy pH 7.4
SV Proton Pump
+Bafilomycin
H+
H+
Rest
EXO
ENDO
AC
bafilomycin
400AP@20Hz
EXO & ds-ENDO/AC
ps-ENDO/AC
EXO
normalized ΔF
time (s)
B
Scr
Sh4
Syb2O
tGFP
Merge
C
20Hz
Scr
Sh1
Sh4
Normalized ΔF
time (s)
D
Peak Fluorescence
τ decay phase (s)
Scr Sh1 Sh4
E
Normalized ΔF
time (s)
τ rising phase (s)
Scr Sh1 Sh4

Figure S3

Figure S3 (related to Fig. 3). PRRT2 knockdown by either Sh1 or Sh4 slows down the kinetics of release in low-density hippocampal neurons as evaluated by the Syb2O reporter of exo-endocytosis

A. Schematic diagram illustrating the pH-sensitive features of synaptophysin-pHluorin (SypHy) to assay SV cycling. *Upper panel*: SypHy fluorescence is quenched by the lumenal acidic pH (5.5) of SVs at rest. Upon exocytosis (EXO) fluorescence increases due the exposure of lumenal EGFP to the extracellular pH (7.4). Endocytosis (ENDO) and the subsequent acidification (AC) of SVs quench the SypHy fluorescence again, a process that can be blocked by H^+-ATPase inhibitors such as bafilomycin. *Lower panel*: Representative time-course of SypHy fluorescence. EXO is induced by repetitive field stimulation (400APs@20Hz) in the absence (*left*) or presence (*right*) of bafilomycin. The blue line represents the rate of SypHy fluorescence increase during stimulation and is contributed by EXO + during-stimulus (DS) ENDO/AC. The green line indicates the rate of SypHy fluorescence decay after stimulation and reflects post-stimulus (PS) ENDO/AC. The rate of SypHy fluorescence increase during stimulation in presence of bafilomycin (red line) represents pure EXO.

B. Representative experimental field showing hippocampal neurites co-expressing Syb2O and PRRT2 Sh4 or Scr (tGFP). The merge panel shows the virtually complete colocalization of the two markers at synaptic puncta. Scale bar, 10 μm.

C. Ensemble averaged Syb2O traces from PRRT2-KD synapses (Sh1, green trace; Sh4, red trace) and control synapses (Scr, black trace) recorded in response to electrical field stimulations at 20 Hz for 20s (gray horizontal line) and normalized to the maximum fluorescence intensity reached under NH_4Cl perfusion (Normalized ΔF). Data are means ± SEM values shown every five time points.

D. Quantitative evaluation of the SV pool released during the stimulation (left panel) and plotted as peak fluorescence reached at the end of the stimulation for Sh1- (green), Sh4- (red) and Scr- (black) treated synapses and of the kinetics of post-stimulus endocytosis (τ decay phase; right panel) determined by exponential fitting of individual experiments for neurons treated with Scr (black bars), Sh1 (green bars) or Sh4 (red bars) shRNA.

E. Kinetics of the fluorescence rising phase during stimulation in the experiments shown in (D) and relative rates (τ) determined in the exponential fitting procedure.

With both shRNAs, the data precisely reproduce the changes induced by PRRT2 silencing that were evaluated by Syphy in Fig. 3. Data are expressed as mean ± SEM from 7 (Sh1, 120 synapses), 7 (Sh4, 140 synapses) and 6 (control, 130 synapses) experiments from 3 different preparations. $^*p<0.05$, $^{**}p<0.01$ *vs* Scr, ANOVA on Ranks/Dunn's multiple comparison test.

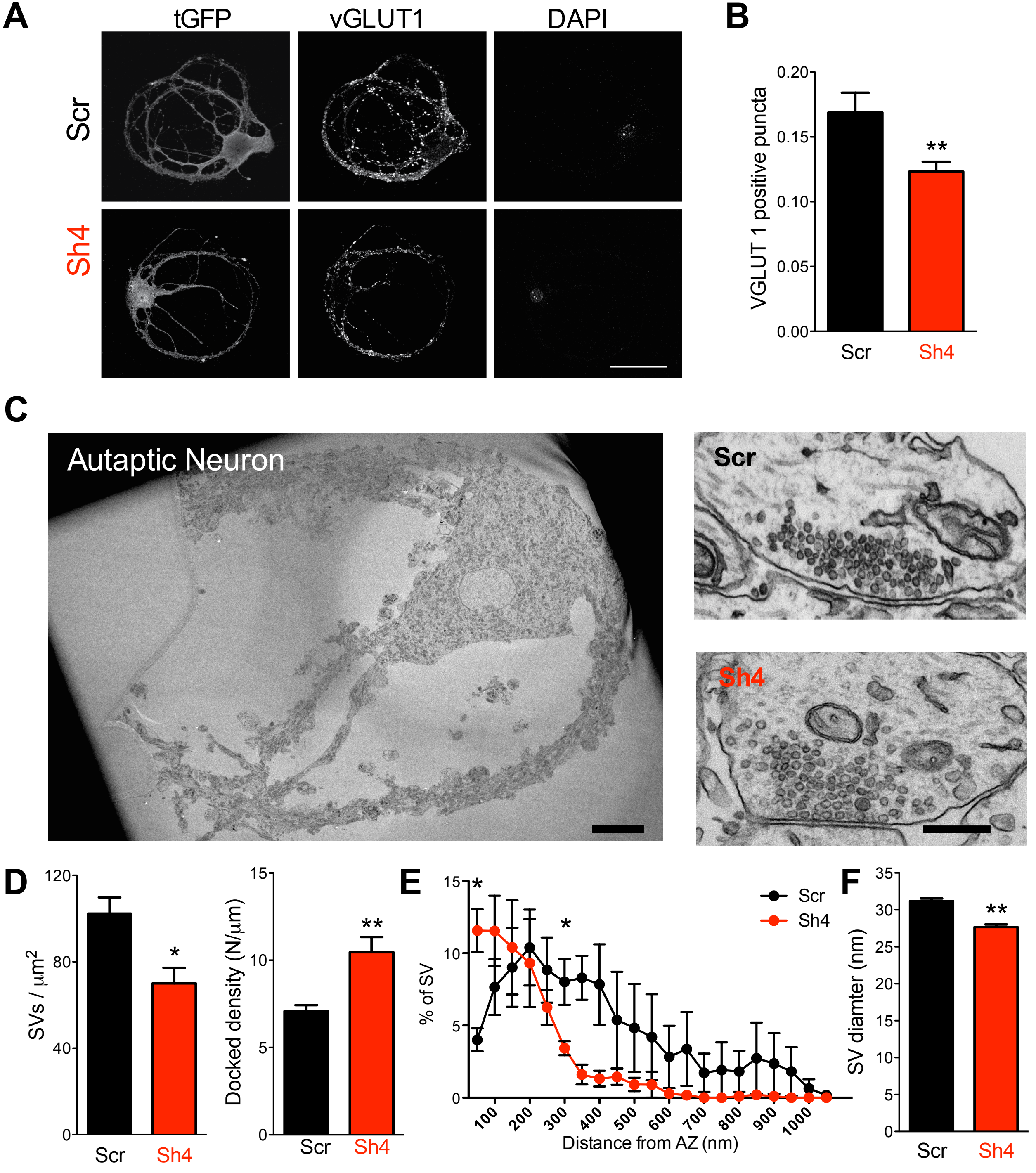
A
tGFP
vGLUT1
DAPI
Scr
Sh4
B
VGLUT 1 positive puncta
0.20
0.15
0.10
0.05
0.00
**
Scr
Sh4
C
Autaptic Neuron
Scr
Sh4
D
SVs / μm2
120
80
40
0
*
Scr
Sh4
Docked density (N/μm)
15
10
5
0
**
Scr
Sh4
E
% of SV
15
10
5
0
*
*
Scr
Sh4
100
200
300
400
500
600
700
800
900
1000
Distance from AZ (nm)
F
SV diamter (nm)
35
30
25
20
15
10
5
0
**
Scr
Sh4

Figure S4

Figure S4 (related to Fig. 4). Silencing of PRRT2 in autaptic hippocampal neurons decreases the number of synaptic contacts and alters SV pools

A. Representative images of autaptic neurons transduced with either Scr or PRRT2 shRNA (Sh4; tGFP fluorescence) and stained with an anti-vGLUT1 antibody. Scale bar, 20 μm.

B. Quantitative evaluation of the density of vGLUT1-positive puncta. Values are expressed as means ± SEM (n = 30 for both control and PRRT2-KD neurons).

C. Ultrastructural analysis of autaptic synaptic contacts. Representative EM images of control and silenced autaptic neurons: from left to right: low magnification electron micrograph of an entire autaptic neuron (scale bar, 20 μm) and close-ups of control and PRRT2-KD autaptic neurons at 14 DIV (scale bar, 200 nm). The synaptic area and active zone length were comparable in control and silenced autaptic neurons (synaptic area: 1.039 ± 0.099 μm^2 and 0.967 ± 0.062 μm^2; active zone length: 0.455 ± 0.027 μm and 0.403 ± 0.035 μm; means ± SEM from 86 and 65 autapses for Scr and PRRT2 sh-RNA-infected neurons, respectively from 5 independent neuronal preparations).

D. Morphometric analysis of control and PRRT2-KD synapses. PRRT2 KD synapses show a decreased number of SVs with a higher number of SV docked at the AZ.

E. Distribution of SVs in autaptic terminals of Scr-treated (black symbols) and Sh4-treated (red symbols) neurons. The percentage of SVs located within successive 50 nm shells from the AZ is given as mean ± SEM as a function of the distance from the AZ.

F. Morphometric analysis of SV diameter in control and PRRT2-KD synapses.

Data are shown as means ± SEM from n= 86 and 65 for control and PRRT2-KD neurons, respectively from n=5 independent preparations. *p<0.05, **p<0.01, unpaired Student's *t*-test (D,F) and Kolgomorov-Smirnov test (E).

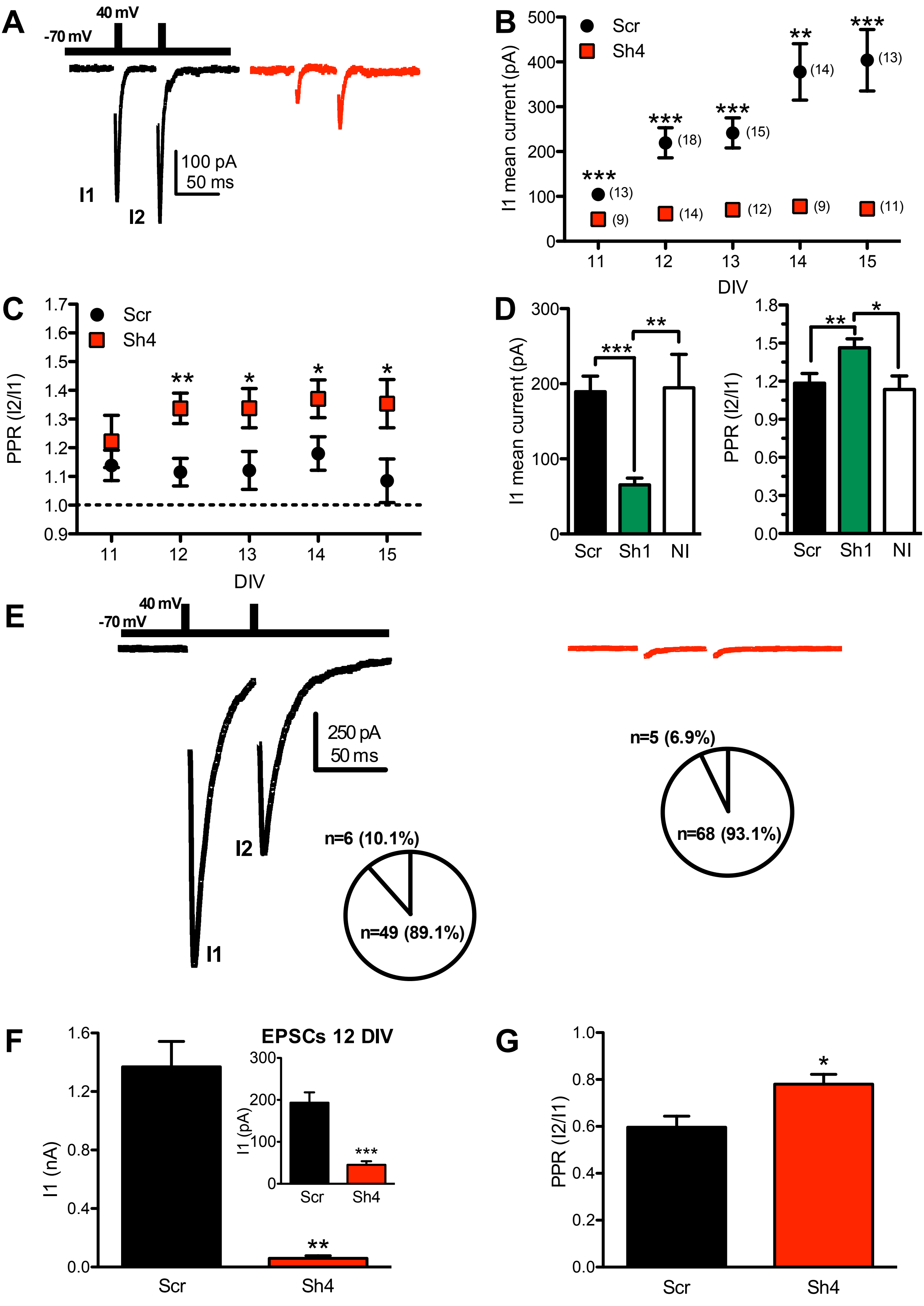

A
40 mV
-70 mV
100 pA
50 ms
I1
I2
B
Scr
Sh4
I1 mean current (pA)
DIV
(13)
(9)
(18)
(14)
(15)
(12)
(14)
(9)
(13)
(11)
C
Scr
Sh4
PPR (I2/I1)
DIV
D
I1 mean current (pA)
PPR (I2/I1)
Scr
Sh1
NI
E
40 mV
-70 mV
250 pA
50 ms
I2
I1
n=6 (10.1%)
n=49 (89.1%)
n=5 (6.9%)
n=68 (93.1%)
F
EPSCs 12 DIV
I1 (nA)
I1 (pA)
Scr
Sh4
G
PPR (I2/I1)
Scr
Sh4

Figure S5

Figure S5 (related to Fig. 4) Knockdown of PRRT2 impairs PSCs in both glutamatergic and GABAergic hippocampal autaptic neurons

A. Representative recording traces of eEPSCs from PRRT2-KD synapses (Sh4, red traces) and control synapses (Scr, black trace).

B,C. eEPSC amplitude evoked by the first pulse (I1; **B**) and paired pulse ratio (PPR=I2/I1; **C**) in neurons transduced with Scr (black symbols) or Sh4 (red symbols) as a function of the days in vitro (DIV). The number of cells recorded in n=3 independent cell culture preparations ranged between 9 and 18.

D. Average eEPSCs amplitude (I1; left) and PPR (right) evoked in autaptic cells infected with lentiviral vectors coding for either Scr (n=24; black bars) or PRRT2-Sh1 (n= 20; green bars). Data obtained in non-infected cells (n= 10; open bars) are shown for comparison.

E. Representative eIPSCs recorded in autaptic neurons infected with either Scr (black trace) or Sh4 (red trace). eIPSCs were evoked by two voltage steps to +40 mV, lasting 0.5 ms, applied at an interpulse interval of 50 ms from a holding potential of -70 mV. Inset: pie plots showing the percent of inhibitory neurons responding to electrical stimulation with respect to the total neurons transduced with either Scr (left) or Sh4 (right). In all synaptic currents, the Na^+ current was blanked for clarity.

F,G. Average eIPSC amplitude (**F**) and PPR (**G**) in neurons transduced with either Scr (black bar; n=6) or Sh4 (red bar; n=5) and recorded at 11-15 DIV. The inset in B shows the change of EPSCs evoked in glutamatergic autaptic neurons in the very same culture preparations and recorded at 12 DIV (Scr: black bars, n=9; Sh4: red bars, n=6).

All data are expressed as means ± SEM. *p<0.05; **p<0.01, ***p<0.001; panels B,C,F,G: unpaired Student's *t*-test or Mann-Whitney U test; panel D: one-way ANOVA/Bonferroni's multiple comparison test (I1) and Kruskal-Wallis/Dunn's multiple comparison test (PPR).

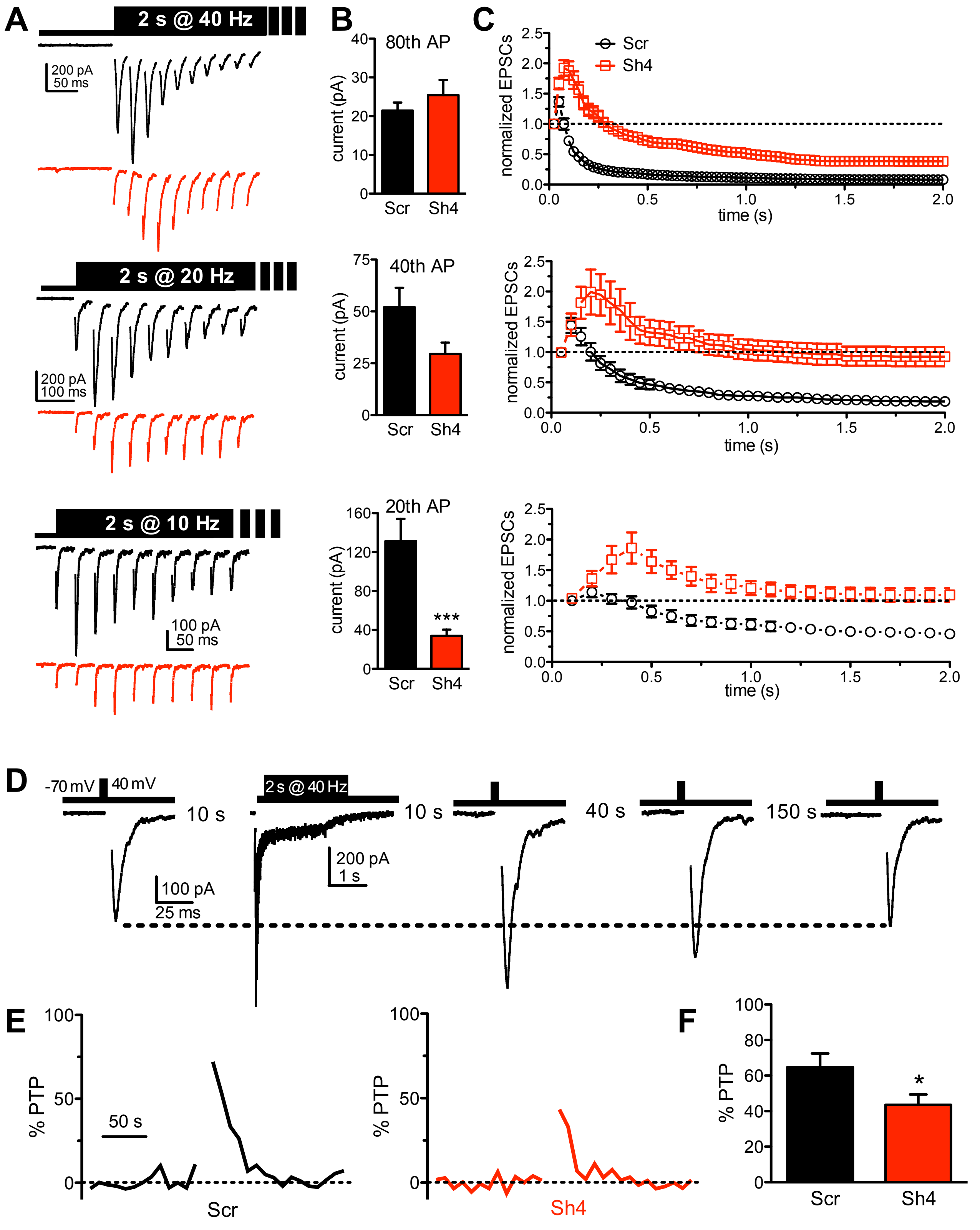

A
2 s @ 40 Hz
200 pA
50 ms
2 s @ 20 Hz
200 pA
100 ms
2 s @ 10 Hz
100 pA
50 ms
B
80th AP
current (pA)
Scr
Sh4
40th AP
20th AP
***
C
Scr
Sh4
normalized EPSCs
time (s)
D
-70 mV
40 mV
10 s
2 s @ 40 Hz
100 pA
25 ms
200 pA
1 s
40 s
150 s
E
% PTP
50 s
Scr
Sh4
F
*

Figure S6

Figure S6 (related to Fig. 6). PRRT2 knockdown dramatically enhances synaptic facilitation and reduced post-tetanic potentiation at excitatory autapses

A. PRRT2 knockdown enhances synaptic facilitation and attenuates synaptic depression. Representative traces showing synchronous EPSCs evoked by tetanic stimulation of 2 s @ 40 (top), 20 (middle) and 10 (bottom) Hz in autaptic neurons transduced with either Scr (black) or PRRT2-Sh4 (red) (40 Hz: Scr n=38, Sh4 n=34; 20 Hz: Scr n=14, Sh4 n=11; 10 Hz: Scr n=12, Sh4 n=11). Stimulation artifacts were blanked for clarity. Only the first 10 currents in the train are shown.

B. Mean ± SEM of the absolute values of the last eEPSC in the train in autaptic neurons transduced with either Scr (black bars) or Sh4 (red bars) are shown.

C. Mean (± SEM) normalized values of eEPSCs amplitude showing the time course of synaptic facilitation and depression in autaptic neurons treated as in A.

D. PRRT2 knockdown decreases post-tetanic potentiation**.** Representative eEPSCs evoked before and 10, 40 and 150 s after tetanic stimulation (2 s@40 Hz). Stimulation artifacts were blanked for clarity.

E. Representative time-course of the normalized eEPSC amplitude in an autaptic neuron infected with either Scr (black trace) or Sh4 (red trace).

F. Mean (± SEM) percentages of post-tetanic potentiation in autaptic neurons transduced with Scr (n=30) or Sh4 (n=28). All data are expressed as means ± SEM. *p<0.05, ***p<0.001, unpaired Student's *t*-test or Mann-Whitney's U-test.

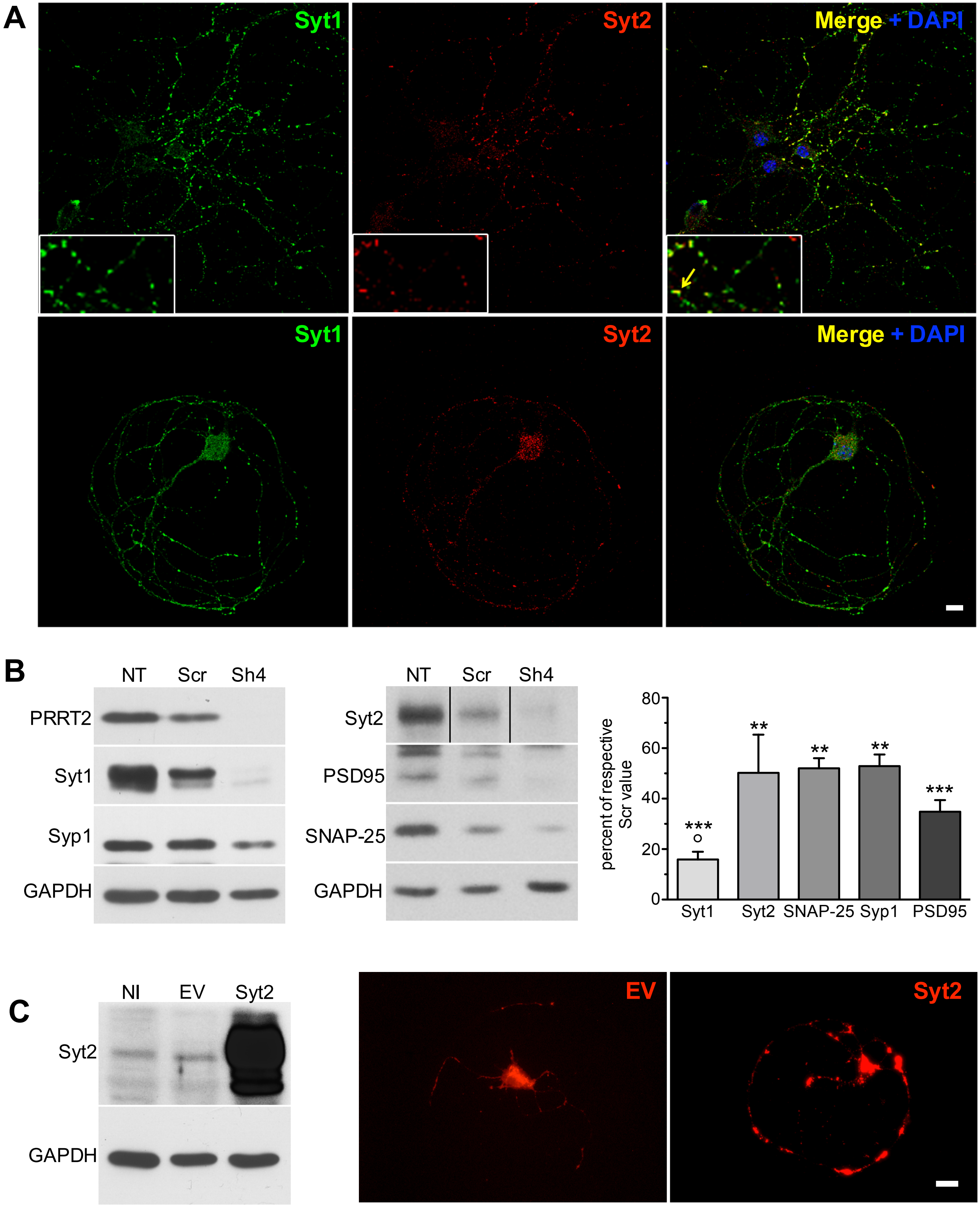
A
Syt1
Syt2
Merge + DAPI
Syt1
Syt2
Merge + DAPI
B
NT
Scr
Sh4
PRRT2
Syt1
Syp1
GAPDH
NT
Scr
Sh4
Syt2
PSD95
SNAP-25
GAPDH
percent of respective Scr value
80
60
40
20
0
***
°
**
**
**
***
Syt1
Syt2
SNAP-25
Syp1
PSD95
C
NI
EV
Syt2
Syt2
GAPDH
EV
Syt2

Figure S7

Figure S7 (related to Fig. 7). Expression of Syt1 and Syt2 in low-density and autaptic primary hippocampal neurons after PRRT2 silencing

A. Double-staining of low-density hippocampal neurons (*top row*) or autaptic hippocampal neurons (*bottom row*) with rabbit Syt1 antibodies (1:250; green) and mouse Syt2 antibodies (1:25; red). In the merge panel shown on the right, nuclei are stained with DAPI (blue). Insets: higher magnification of synaptic boutons shows partial co-localization of the two Syt isoforms (arrow). Scale bar, 10 μm.

B. Expression levels of pre- and post-synaptic proteins in low-density hippocampal neurons transduced with either Scr or Sh4 or left non-infected. Neurons were infected at 7 DIV and harvested at 15 DIV. Samples were separated by SDS-PAGE and analyzed by immunoblotting with antibodies to Syt1, Syt2, SNAP-25, synaptophysin-1 (Syp1) and PSD95. *Left:* a representative immunoblot is shown. Vertical lines in the blot indicate that the lanes were on the same gel, but have been repositioned in the figure. Immunoreactivity for GAPDH was used as a control for equal loading. *Right:* Immunoblots were quantified by densitometric analysis. The expression level of the various presynaptic proteins in PRRT2-KD neurons, calculated in percent of the levels in Scr-treated neurons, is shown as means ± SEM of n=5 independent experiments. $^{**}p<0.01$, $^{***}p<0.001$ *vs* Scr-treated neurons; $^{\circ}p<0.05$ Syt1 *vs* other synaptic proteins; ANOVA/Dunnett's multiple comparison test.

C. Overexpression of Syt2 in low-density and autaptic primary neurons. Left: representative western blot of Syt2 expression in low-density hippocampal neurons that were non-infected (NI), infected with the empty vector (EV) or infected with the Syt2 overexpressing vector. Immunoreactivity for GAPDH was used as a control for equal loading. Right: Syt2 immunoreactivity in autaptic neurons that were transduced with either empty vector (EV) or Syt2 overexpressing vector. Scale bar, 10 μm.

SUPPLEMENTAL EXPERIMENTAL PROCEDURES

Generation of PRRT2 shRNAs and sh-resistant PRRT2. Based on the ORF of mouse PRRT2 transcript, 5 shRNAs were designed (Mission shRNA custom cloning, Sigma-Aldrich):

Sh1: GGCCACAGACCTCAGTTTAAA
Sh2: GAGGTCCTGACAGAAAGTACA
Sh3: CTCGGGACTATATCATCCTTG
Sh4: CATGTGGCCTGTCAACATTGT
Sh5: TGTCCAGGTTGTAGCAGAGAT

As a negative control, we used the following scrambled ShRNA sequence, based on the sequence of Sh1: GCCGACACTCGTCAGTTTAAA. An additional control ShRNA, with a fully unrelated sequence that does not target any known human or mouse genes (Luciferase shRNA; Sigma-Aldrich) was occasionally used with similar results. Sequence alignment of human and mouse PRRT2 regions targeted by the five shRNAs (see below, highlighted in yellow) revealed the presence of several mismatches with the human sequence, accounting for the specificity of the shRNAs in knocking down the endogenous mouse PRRT2 expression.

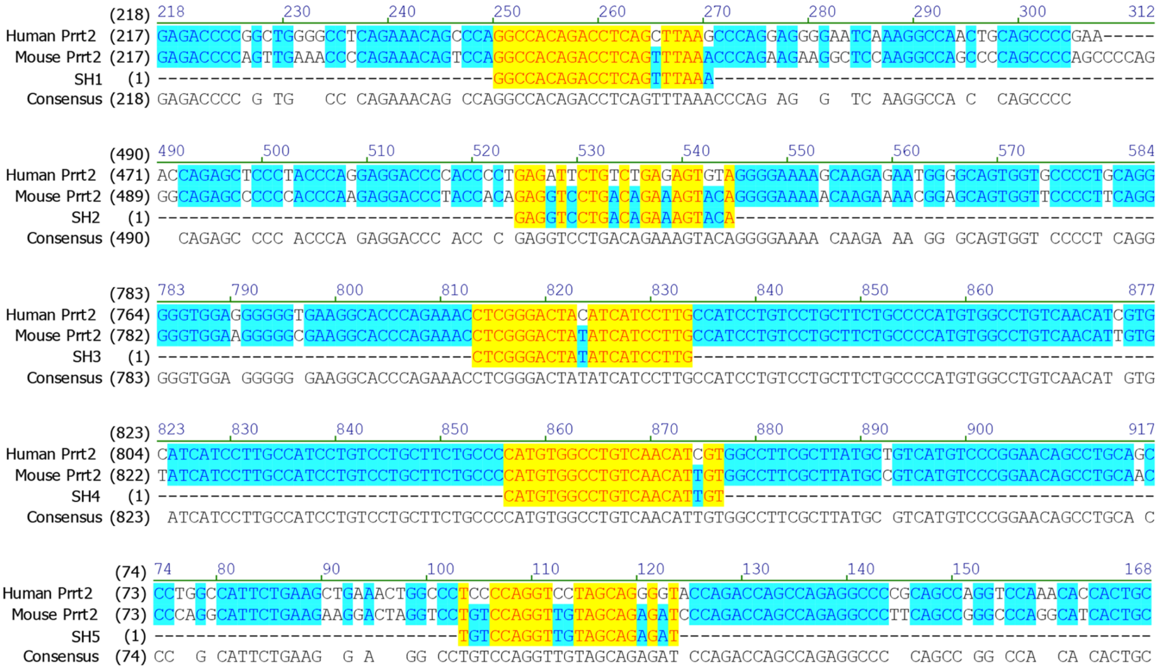

For rescue experiments, we generated PRRT2-Cherry Sh1-res and PRRT2-Cherry Sh4-res constructs carrying silent mutations that render them insensible to knocking-down.

SH1 resistant: GGCCACAGACCTGAGCCTGAA
SH4 resistant: CATGTGGCCAGTGAATATCGT

Synaptosome preparation. Synaptosomes were purified from the P2 fractions by centrifugation on discontinuous Percoll gradient from the pooled cortex of three adult mice (3-6 months old). The tissue was homogenized in 10 ml of ice-cold HB buffer (0.32 M sucrose, 1 mM EDTA, 10 mM Tris, pH 7.4) containing protease inhibitors, using a glass-Teflon homogenizer. The resultant homogenate was centrifuged at 1,000 x g for 5 min at 4°C in order to remove nuclei and debris and the supernatant was further centrifuged at 18,900 x g for 10 min at 4°C to obtain the P2 fraction. The pellet was resuspended in 2 ml of HB buffer and gently stratified on a discontinuous Percoll gradient (3%-10%-23%). After a 10 min centrifugation at 18,900 x g at 4°C, the synaptosomal fraction from the 10% and 23% Percoll interface was collected, washed in Krebs buffer (140 mM NaCl, 5 mM KCl, 5 mM $NaHCO_3$, 1.3 mM $MgSO_4$, 1 mM phosphate buffer pH 7.4, 10 mM Tris/Hepes pH 7.4) to eliminate Percoll and used as the starting material for subsequent ultrafractionation.

Ultrasynaptic fractionation. Ultrasynaptic fractionation was performed as previously described (Phillips et al., 2001; Feligioni et al., 2006). Briefly, synaptosomes were pelleted by centrifugation (16,000 x g, 5 min, 4°C) and resuspended in 300 µl of 0.32 M sucrose, 0.1 mM $CaCl_2$. An aliquot was removed and kept as total. Protease inhibitors were used in all purification steps. Synaptosomes were then diluted 1:10 in ice-cold 0.1 mM $CaCl_2$ and mixed with an equal volume of 2X solubilization buffer (2 % Triton X-100, 40 mM Tris, pH 6, 4°C). After

a 30 min incubation at 4°C, the insoluble material (synaptic junction) was pelleted by centrifugation (40,000 x g, 30 min, 4°C). The supernatant (non-synaptic synaptosomal protein; NSSP) was decanted and the proteins precipitated with 6 volumes of acetone at -20°C overnight and then centrifuged (18,000 x g, 30 min, -15°C). The synaptic junction pellet (containing the insoluble postsynaptic density and the presynaptic active zone) was resuspended in 10 volumes of 1X solubilization buffer (1% Triton X-100, 20 mM Tris, pH 8, 4°C), incubated for 30 min at 4°C and then centrifuged (40,000 x g, 30 min, 4°C). The proteins in the supernatant (active zone, AZ) were precipitated in 6 volumes of acetone at -20°C overnight and then centrifuged (18,000 x g, 30 min, -15°C), while the pellet was kept as postsynaptic density fraction (PSD). All pellets (PSD, AZ, NSSP) were resuspended in 5% SDS, quantified by BCA assay, and loaded on SDS-PAGE gels for electrophoresis and consecutive Western Blotting.

Subcellular fractionation for SV purification. Subcellular fractions were prepared from rat forebrain, and SVs were purified through the step of controlled-pore glass chromatography as described (Huttner et al., 1983). Aliquots of purified SVs were exposed to 200 mM NaCl treatment, which results in quantitative removal of extrinsic SV proteins such as synapsin I. Both untreated and salt-treated SVs (USV and SSV, respectively) were resuspended in 0.3 M glycine, 5 mM Hepes/NaOH, pH 7.4, at a protein concentration of 1.5–2 mg/ml.

Western blotting and antibodies. Protein concentration of the samples was determined using the BCA or Bradford assay and equivalent amounts of protein were subjected to SDS-PAGE on 10-12% polyacrylamide gels and blotted onto nitrocellulose membranes (Whatman). Blotted membranes were blocked for 1 h in 5% milk in Tris-buffered saline (10 mM Tris, 150 mM NaCl, pH 8.0) plus 0.1% Triton X-100 and incubated overnight at 4°C with the following primary antibodies: rabbit anti-PRRT2 (1:500, Sigma-Aldrich), rabbit anti-synaptophysin-1 (1:5000, Synaptic Systems), rabbit anti-SNAP-25 (1:1000, Cell Signaling), mouse anti-PSD95 (1:3000, Synaptic Systems), rabbit anti-Synaptotagmin-1 (1:1000, Synaptic Systems), rabbit anti-Synaptotagmin-2 (1:1000, Synaptic Systems), rabbit anti-Syntaxin 1A (1:1000, Synaptic Systems), rabbit anti-Vamp2 (1:1000, Synaptic Systems), rabbit anti-Munc18-1 (1:2000, Synaptic Systems), rabbit anti-Complexin 1/2 (1:2000, Synaptic Systems), rabbit anti-RIM1 (1:1000, Synaptic Systems), rabbit anti-RIM binding protein-1 (1:1000, Synaptic Systems), rabbit anti-GAPDH (1:10000, Cell Signaling), rabbit anti-calnexin (1:15000, Sigma-Aldrich). Membranes were washed and incubated for 1 h at room temperature with peroxidase-conjugated goat anti-rabbit (1:5000; Bio-Rad) or with peroxidase-conjugated goat anti-mouse (1:5000; Bio-Rad) antibodies. Bands were revealed with the ECL chemiluminescence detection system (Thermo Scientific, Waltham, Ma, USA).

Pull down and co-immunoprecipitation assays. Nterm-3XFlag-PRRT2 and Flag-bacterial alkaline phosphatase (3XFlag-BAP) as a control (Sigma-Aldrich, cat. E7658) were overexpressed in HEK293T cells and purified by anti-Flag M2 Affinity gel (Sigma-Aldrich). The immunocomplex was incubated for 4 hrs with crude synaptosome extracts (P2) prepared from mouse brain in lysis buffer (1% Triton X-100, 150 mM NaCl, 50 mM TrisHCl pH 7.4, 1 mM EDTA pH 8.0, with protease inhibitor cocktail) to allow the binding of potential interacting proteins. Pulled down proteins together with aliquots of the input material and the supernatants were analyzed by Western blotting with antibodies directed against presynaptic proteins. For immunoprecipitation (IP), 10 μg of either anti-Syt1 antibodies (mouse clone 41.1, Synaptic Systems) or anti-Syt2 antibodies (mouse ZNP-1; Abcam) or mouse/rabbit control IgGs (Sigma-Aldrich) were pre-coated with Protein G Sepharose resin (GE Healthcare) overnight and incubated with total mouse brain lysate in IP buffer (150 mM NaCl, 50 mM Tris-HCl pH 7.4, 2 mM EDTA, 1% Triton X-100). After extensive washes in IP buffer and detergent-free IP buffer, samples were resolved by SDS-PAGE and subjected to western blotting with anti-PRRT2 antibodies.

RNA extraction and real time PCR. Total RNA was extracted by using the RNeasy Mini or Micro Kit (Qiagen) and then treated with DNase with the DNA-free DNA Removal kit (Ambion), following manufacturer's instructions. RNA concentration was quantified by using the Nanodrop-1000 spectrophotometer (Thermo Scientific). Retrotranscription was performed on equal amounts of RNA by using the M-MLV Reverse Transcriptase and random primers (Invitrogen), following manufacturer's instructions and including RT-negative controls. Real time PCR analyses were performed using the SYBR Green I Master mix (Roche), on a Lightcycler 480 (Roche), with the following protocol: 95°C for 5 min; 10 s at 95°C / 20 s at the specific annealing temperature (T_a) / 10 s at 72°C for 45 cycles; melting curve (heating ramp from 55°C to 95°C) in order to check for amplification specificity. The following primers (final concentration 0.25 μM) and annealing temperatures were used: *PRRT2* For-1 AGGTAGCCTAAGCCGTCATCC, *PRRT2* Rev-1 CAGGCTGTTCCGGGACATGAC, T_a=61°C; *PRRT2* For-2 CATCGCCTCCTGCGTCATCAAC, *PRRT2* Rev-2: CTCAGGCTCCCTTGGTCCCTAG, T_a=63°C; *H3f3a* For GTGAAGAAACCTCATCGTTACAGGCCTGGT, *H3f3a* Rev CTGCAAAGCACCAATAGCTGCACTCTGGAA, T_a=64°C; *Tbp* For GAGCTCTGGAATTGTACCGCAG, *Tbp* Rev CATGATGACTGCAGCAAATCGC, T_a=62°C; *Tubb3* For GCGTATACTACAATGAGGCCTCC,

Tubb3 Rev GTTGCCAGCACCACTCTGACC, T_a=63°C; *Hprt* For CAGACTGAAGAGCTACTGTAATG, *Hprt* Rev GGGCTGTACTGCTTAACCAGG, T_a=62°C. Relative quantification of *PRRT2* expression was made using the $2^{-\Delta\Delta Ct}$ method (Pfaffl, 2001), normalizing data to the geometric mean of three housekeeping transcripts (*H3f3a*, *Tbp*, *Hprt* for primary neurons and *H3f3a*, *Tbp*, *Tubb3* for tissue samples) (Vandesompele et al., 2002) and to a calibrator (developmental stage at which the peak of expression was observed: 10 DIV for primary hippocampal neurons, 14 DIV for primary cortical neurons and P60 for tissue samples).

Cell culture procedures. Low-density and autaptic hippocampal neurons were prepared from C57BL6/J mice as previously described (Baldelli et al., 2007). Animals were sacrificed by CO_2 inhalation, and 17/18-day embryos (E17-18) were removed immediately by cesarean section. In brief, hippocampi or cerebral cortices were dissociated by enzymatic digestion in 0.125% Trypsin for 20 min at 37°C and then triturated with a fire-polished Pasteur pipette. No antimitotic drugs were added to prevent glia proliferation. Autaptic neurons were prepared as described previously (Bekkers and Stevens, 1991; Chiappalone et al., 2009) with slight modifications. Dissociated neurons were plated at very low density (20 cells/mm^2) on microdots (40-300 μm in diameter) obtained by spraying a mixture of poly-D-lysine (0.1 mg/ml) and collagen (0.25 mg/ml) on petri dishes, previously pretreated with 0.15% agarose. Both glial cells and single autaptic neurons were present under this culture condition.

Immunocytochemistry and analysis of synapse density. Neurons were fixed at 14 DIV (7 days post-infection) in 4% formaldehyde, freshly prepared from paraformaldehyde, in 0.1 M phosphate buffer, pH 7.4 (PB) for 20 minutes at room temperature (RT) and immunostained for SNAP-25, synaptophysin, vGLUT1 and tGFP in various combinations. Briefly, after several washes in phosphate-buffered saline (PBS), cells were permeabilized and blocked for 30 minutes in 5% Normal Goat Serum (NGS), 0.1 % saponin in PBS and then incubated with primary antibodies diluted in 5% NGS and 0.1% saponin in PBS up to two hrs. Antibodies were used as follows: anti-SNAP-25 (1:200; Synaptic Systems), anti-synaptophysin-1 (1:200; Synaptic Systems), anti-VGLUT1 (1:1000; Synaptic Systems #135304), anti-tGFP (1:200; Origene #TA150041). Neurons were then washed twice in 0.1 % saponin or Triton X-100 in PBS and blocked for 10 min in 5 % NGS and 0.1 % saponin or Triton X-100 in PBS before being incubated in the same buffer with Alexa conjugated secondary antibodies (1:500, Invitrogen). After several washes in PBS coverslips were mounted using Prolong Gold anti-fade reagent with DAPI staining (Invitrogen). As a control for the staining and the acquisition procedure the primary antibodies were omitted. Images were acquired using a 63X objective in a Leica SP5 confocal (Leica Microsystem, Vienna).
For the analysis of synapse density, low density hippocampal neurons were infected at 7 DIV and processed for immunofluorescence at 14 DIV. Cells were rinsed once with Krebs–Ringer's solution (KRH)–EGTA (130 mM NaCl, 5 mM KCl, 1.2 mM KH_2PO_4, 1.2 mM $MgSO_4$, 2 mM $MgCl_2$, 2 mM EGTA, 25 mM Hepes, 6 mM glucose, pH 7.4), fixed for 15 minutes with 4% paraformaldehyde, 4% sucrose in 120 mM sodium phosphate buffer, pH 7.4, supplemented with 2 mM EGTA. Coverslips were rinsed twice with phosphate-buffered saline (PBS) and then incubated overnight at 4°C into a humidified chamber with the primary antibodies (mouse anti-Bassoon (1:200, Stressgen) and rabbit anti-Homer 1 (1:150, Synaptic Systems) diluted in goat serum dilution buffer (GSDB; 15% goat serum, 450 mM NaCl, 0.3% Triton X-100, 20 mM sodium phosphate buffer, pH 7.4). Cells were washed three times with PBS and then incubated with the appropriate secondary antibodies for 1 hour at room temperature. After three washes with PBS for 10 min each, coverslips were mounted with Dako fluorescence mounting medium. Specimens were acquired with an Axio Imager.A2 microscope (Zeiss, Oberkochen, Germany) and images were processed using the colocalization plugin of ImageJ: five dendrites per neuron were selected, and the colocalization analysis was performed between images after subtracting an appropriate threshold to evaluate the simultaneous presence of a pre- and a post-synaptic protein (Bsn and Homer 1, respectively). We considered the colocalization puncta with an area in the range of 0.1–1 μm^2, corresponding to an overlapping area of pre-synaptic and post-synaptic proteins to identify *bona fide* synaptic boutons. We counted the puncta present within 30 μm dendrite tract starting from the cell body. Data are referred to three independent experiments carried out in duplicate. Ten neurons for each sample were analyzed. Statistical analysis was performed using the Student's t-tests, comparing the Sh-RNA treated sample to the corresponding scramble control.

Live imaging of exo-endocytosis. Hippocampal cultures, grown onto poly-L-lysine-treated glass coverslips, were put in the stimulation chamber (Warner Instruments, Hamden, CT), immersed in Tyrode Solution (140 mM NaCl, 3 mM KCl, 2 mM $CaCl_2$, 1 mM $MgCl_2$, 10 mM HEPES-buffered to pH 7.4, 10 mM glucose) and located on the stage of an IX-81 motorized inverted epifluorescence microscope (Olympus, Hamburg, Germany). The imaging buffer was supplemented with glutamate receptor inhibitors (CNQX 10 μM; APV 50 μM; TOCRIS) to reduce spontaneous activity and prevent recurrent excitation during stimulation. An MT20 Hg-Xe lamp (Olympus) was used as light source with 480±20 nm excitation, 495 nm dichroic and 525±50 nm emission filters to detect the GFP (SypHy or tGFP) signal and 560±40 nm excitation, 585 nm dichroic and 630±75 nm

emission filters to detect the SypHy m-Orange2 or the m-Cherry signal. Time-lapses were acquired at 1 Hz for 100 s with an Orca-ER IEEE1394 CCD camera (Hamamatsu Photonics, Hamamatsu City, Japan) using a UplanSapo 60X1.35 NA oil-immersion objective (Olympus). Cells were maintained in a saline solution at room temperature through a laminar-flow perfusion and the selected field was stimulated after 10 seconds of baseline acquisition. Action potentials (AP) were evoked by passing 1-ms current pulses through platinum-iridium electrodes using a AM 2100 stimulator (AM-Systems, Carlsborg, WA). One image on GFP/mCherry channel was acquired at the beginning of each experiment to verify the PRRT2 silencing on the processes to be analyzed by pHluorin assay. Circular ROIs of 1.7 mm diameter were positioned manually at the center of each responsive synapse.

To evaluate the effects of PRRT2 silencing on SV trafficking, stimulation protocols were applied to estimate the release from either the readily releasable pool (40 action potentials @20Hz) or the recycling pool (400 action potentials@20Hz) (Fassio et al., 2011). After 10 s of baseline acquisition (F_0), neurons were stimulated in the presence or absence of 1 μM bafilomycin and finally subjected to alkalinization by perfusion with 50 mM NH_4Cl (F_{max}). Images were analyzed using the Xcellence RT software (Olympus). The total increase in the fluorescence signal (ΔF) was calculated by subtracting F_0 and the ΔF was normalized to the fluorescence value obtained by alkalization of the entire vesicle pool using NH_4Cl (ΔF_{max}). The time constant of endocytosis (τ decay phase) was calculated by the fitting the post-stimulus decay with a single-exponential function. To get release rates, the traces recorded during stimulation were fitted as single exponential functions and the relative kinetics were plotted as time constants of release (τ rising phase). N refers to the number of coverslips analyzed from 3-4 different preparations. Data were collected from 20-40 boutons per coverslip. Exocytosis studied by grossly corresponds to the time integral of release in response to a long-lasting (20 sec) high-frequency (20 Hz) stimulation that includes the synchronous release, its dynamics of depression and the asynchronous release. Therefore, it cannot be compared with the amplitude of EPSCs evoked by single Aps, but rather with the integral of the charge released during sustained high-frequency stimulations.

Transmission Electron Microscopy. Autaptic hippocampal neurons and low-density cultures of hippocampal neurons were infected at 7–11 DIV with either scramble shRNA or PRRT2 shRNAs and processed for transmission electron microscopy (TEM). To unambiguously identify transduced neurons, we correlated the tGFP fluorescence signal with EM analysis. To do so, we identified tGFP-positive neurons at the epifluorescence microscope and marked their position by scratching the coverslip around the neuron with a pencil point diamond-tip glass-cutter. Neurons were fixed at 14–18 DIV with 1.2% glutaraldehyde in 66 mM sodium cacodylate buffer, post-fixed in 1% OsO_4, 1.5% $K_4Fe(CN)_6$, 0.1 M sodium cacodylate, *en bloc* stained with 1% uranyl acetate, dehydrated, and flat embedded in epoxy resin (Epon 812, TAAB). After baking for 48 hrs, the position of the scratches on coverslips was detected under a stereomicroscope and marked on the resin block with a marker pen. The glass coverslip was removed from the Epon block by thermal shock and isolated autaptic neurons were identified by means of a stereomicroscope with the help of the pen marker positional clue. Embedded neurons were the excised from the block, and mounted on a cured Epon block for sectioning using an EM UC6 ultramicrotome (Leica Microsystems). Ultrathin sections (60–70 nm thick) were collected on 200-mesh copper grids (EMS) and observed with a JEM-1011 electron microscope (Jeol, Tokyo, Japan) operating at 100 kV using an ORIUS SC1000 CCD camera (*Gatan,* Pleasanton, CA). Low-density cultured neurons were studied using the same procedure. For each experimental condition, at least 30 images of synapses were acquired at 10,000x magnification (sampled area per experimental condition: 36 μm^2). Synaptic profile area, SV number, and distribution relative to the active zone (AZ) were determined using ImageJ software. For the 3D reconstruction, the standard TEM sample preparation protocol was followed, and samples were embedded in Epon resin. Serial 60-nm-thick sections were collected on carbon-coated copper slot formvar and carbon-coated grids, and serial synaptic profiles acquired. Serial sections were aligned using the Midas of IMOD. Synapses with one single AZ, at least one docked SV and >100 but <800 total SVs were reconstructed with the software IMOD. The quantitative analysis of synaptic density was performed using NIH ImageJ software. A counting frame of known area (8.67 x 5.78 μm) was created using Adobe Illustrator CS5 software and was superimposed to the hippocampal image; only the asymmetric and symmetric synapses present in each image and intersecting the right and superior edges of the frame were counted to obtain the synapse density. Volume density of the synapses was calculated from the two-dimensional count of synaptic profile, using the stereological formula $N_{vs}= N_a/(H+t)$, where N_a is the number of synapses per unit area, t is the section thickness and H is the mean tangent diameter ($H = (\pi D)/4$, where D is the mean diameter of the synaptic contact), as previously described (Kurt et al., 2004). For the analysis of synaptic ultrastructure under stimulation, primary hippocampal neurons were infected as described above. Infected neurons were field stimulated at 14-18 DIV (7 days post-infection) with a train stimulation protocol of 30 s @ 10Hz with an insulated pulse stimulator (A-M Systems). Experiments were performed in Tyrode's buffer containing 50 μM APV and 10 μM CNQX. Immediately after the end of the train (0 s) and after 20 min recovery, neurons were fixed for electron microscopy in 1.25% glutaraldehyde in 66 mM sodium cacodylate buffer at 37 °C and then processed as described for the

conventional transmission electron microscopy. Under these conditions, we estimated a complete fixation of synapses within 1-3 s from fixative addition (Leung, 1994).

Patch-clamp recordings. Patch pipettes, prepared from thin-borosilicate glass (Hilgenberg, Mansfield, Germany), were pulled and fire-polished to a final resistance of 2-4 MΩ when filled with standard internal solution. Evoked postsynaptic currents (ePSCs) were recorded using a double EPC-10 amplifier (HEKA Electronic, Lambrecht, Germany). For whole-cells recordings, cells were maintained in a standard external solution containing (in mM): 140 NaCl, 2 $CaCl_2$, 1 $MgCl_2$, 4 KCl, 10 glucose, 10 HEPES (pH 7.3 with NaOH). When using external solutions with different ionic composition, salts were replaced equimolarly. To record eEPSCs, where otherwise not indicated, D-(-)-2-amino-5-phosphonopentanoic acid (D-AP5; 50 μM; Tocris, Bristol, UK) and bicuculline methiodide (30 μM, Tocris) were added to the Tyrode solution to block N-methyl-D-aspartate (NMDA) and $GABA_A$Rs, respectively. To record eIPSCs, 6-cyano-7-nitroquinoxaline-2,3-dione (CNQX; 10 μM) and CGP 58845 (10 μM) were added to the external solution to block non-NMDA and $GABA_B$Rs, respectively. The standard internal solution was (in mM): 126 KGluconate, 4 NaCl, 1 $MgSO_4$, 0.02 $CaCl_2$, 0.1 BAPTA, 15 Glucose, 5 HEPES, 3 ATP, 0.1 GTP (pH 7.2 with KOH). For experiments in which eIPSCs were studied, following internal solution was used (in mM): 120 KGluconate, 4 NaCl, 20 KCl, 1 $MgSO_4$, 0.1 EGTA, 15 Glucose, 5 HEPES, 3 ATP, 0.1 GTP (pH 7.2 with KOH). All experiments were performed at room temperature (22-24 °C). Neurons were voltage-clamped at -70 mV. Unclamped action potentials (APs) were evoked by a brief depolarization of the cell body to +40 mV for 0.5 ms @ 0.1 Hz. eE/IPSCs were acquired at 10-20 kHz sample frequency and filtered at half the acquisition rate with an 8-pole low-pass Bessel filter. Recordings with leak currents >100 pA or series resistance >20 MΩ were discarded. Data acquisition was performed using PatchMaster programs (HEKA Elektronic, Lambrecht, Germany). eE/IPSCs were inspected visually, and only those that were not contaminated by spontaneous activity were considered. To calculate the peak current during an isolated stimulus or a train of stimuli, we first subtracted an averaged trace containing the stimulus artifact and the AP current, but lacking any discernable synaptic current (i.e. synaptic failures). Such traces were easily identified toward the end of a train of stimuli, when synaptic depression was maximal. These traces were averaged and scaled to the peak Na^+ current contaminating the eE/IPSCs.

Short-term plasticity paradigms. To analyze paired-pulse ratio (PPR), two brief depolarizing pulses were applied to autaptic neurons at 50 ms time intervals. For each couple of eE/IPSCs, PPR was calculated as the ratio I_2/I_1, where I_1 and I_2 are the amplitudes of the eEPSCs/eIPSCs evoked by the conditioning (1) and test (2) stimuli, respectively. To correctly estimate the amplitude of I_2, the baseline of I_2 was defined as the final value of the decay phase of I_1 and the amplitude of I_2 was calculated by subtracting the residual amplitude of I_1 from the peak value of I_2. For the evaluation of the synaptic responses during tetanic stimulation, the time interval was shorter than the time needed for an eEPSC to return to baseline, so eEPSCs overlapped partially. Then, to correctly estimate the EPSC amplitude, the baseline of each event was defined as the final value of the decay phase of the preceding eEPSC and the amplitude of eEPSC number n was calculated by subtracting the residual amplitude of the previous eEPSCs from its peak value. For the evaluation of post-tetanic potentiation (PTP), autaptic neurons were depolarized with short-trains of stimuli (2 s @ 40 Hz). The maximal PTP induced by the tetanic stimulation was determined by measuring the maximal amplitude of the eEPSCs, usually obtained 10 or 20 s after the end of the train. The time-course of the PTP was followed with a stimulation frequency to 0.1 Hz.

Cumulative eEPSC amplitude analysis. The size of the readily releasable pool of synchronous release (RRP_{syn}) and the probability that any given SV in the RRP will be released (Pr) were calculated using the cumulative amplitude analysis (Schneggenburger et al.,1999 and 2002; Baldelli et al., 2007). The cumulative amplitude plot was determined by summing up peak EPSC amplitudes during 40 repetitive stimuli applied at 40 Hz. This analysis assumes that depression during the steady-state phase is limited by a constant recycling of SVs and an equilibrium occurs between released and recycled SVs and that Pr_{syn} during the train approaches the 1 value (Schneggenburger et al., 1999). Assuming that the slow linear rise is due to the equilibrium between the release-induced depletion and the constant replenishment of the RRP, back-extrapolation of the linear portion to time zero yields a rough estimation of the size of the RRP current of synchronous release (RRP_{syn}), while the release probability Pr is calculated as the ratio between the first eEPSC (I1) and the RRP_{syn}. The number of data points for the linear fitting of the steady-state phase was evaluated by calculating the best fit including the maximal number of data points starting from the last data point (generally the last 15-20 data points). To estimate the number of SVs (N_{syn}) forming the RRP_{syn}, RRP_{syn} was divided by the unitary current (Q_{av}, corresponding to the average amplitude of autaptic mEPSCs).

Estimation of RRP_{total} by the hypertonic sucrose method. The RRP size of the total release (RRP_{tot}) was estimated using a hypertonic stimulation (Rosenmund and Stevens, 1996; Baldelli et al., 2007). Autaptic neurons were voltage clamped at –70 mV and stimulated by a 1 ms voltage step to 20/100 mV applied at a frequency of 0.05 Hz, evoking an isolated EPSC. One minute later, a hypertonic extracellular solution (supplemented with 500 mM sucrose; Yamaguchi et al., 2002) was focally micro-applied to the same neuron for 6 s. The charge transfer in the transient part of the synaptic current induced by the sucrose application was

measured as RRP_{tot}. The number of RRP_{tot} quanta was then calculated by dividing the RRP_{tot} by the unitary mEPSC charge. The experiments were performed by constantly superfusing the autaptic neurons with a Tyrode external solution. The perfusion solution (flow rate ≈ 200 μl/min) could be rapidly changed (50/60 ms) and puffs of hypertonic solution were applied over controlled periods of time (6 sec). The tip of the perfusion pipette (50/100 μm) was placed close to the soma (80/150 μm).

Asynchronous and spontaneous release analysis. The delayed asynchronous release of the excitatory autapses was evoked by a tetanic stimulation lasting 2 s @ 40 Hz. Asynchronous release was estimated by measuring the area (pA*ms) of the spontaneous EPSCs that follow the stimulation train, in 9 consecutive time-windows lasting 1 s each, for a total time-period of 9 seconds. Miniature EPSCs (mEPSC) were collected from low-density and autaptic neurons incubated in the presence of tetrodotoxin (TTX, 1 μM; Tocris) to block spontaneous action potentials propagation. mEPSC analysis was performed by using the Minianalysis program (Synaptosoft, Leonia, NJ). The amplitude and frequency of mPSCs were calculated using a peak detector function using appropriate threshold amplitude and threshold area.

SUPPLEMENTAL DISCUSSION

The paroxysmal nature of the attacks and the variable phenotypes in patients bearing PRRT2 loss-of-function mutations, together with the association with epilepsy and migraine, suggest the presence of an acute defect in the excitation/inhibition balance. In PRRT2-KD, we observed an impairment in synaptic transmission in both glutamatergic and GABAergic synapses, although the effect seemed more severe for inhibitory transmission. To account for an excitation/inhibition imbalance that explains the human phenotypes, two possibilities might be considered. It is possible that the regional distribution of PRRT2 among excitatory and inhibitory neurons varies in the various brain areas, bringing about shifts in the excitation/inhibition balance associated with PRRT2 haploinsufficiency. An alternative explanation is that the more pronounced effects of PRRT2 KD in inhibitory neurons may engender an anomalous excitation/inhibition imbalance under rare conditions of heightened network activity, due to positive feedback cycles. Such positive feedback cycles lead to activity-dependent reductions in the efficacy of neuronal inhibition through the phenomenon of activity-dependent disinhibition, occasionally crossing the threshold needed to generate an ictal state (Staley, 2015).

SUPPLEMENTAL REFERENCES

Baldelli P, Fassio A, Valtorta F, Benfenati F (2007) Lack of synapsin I reduces the readily releasable pool of synaptic vesicles at central inhibitory synapses. J Neurosci 27, 13520-13531

Bekkers JM, Stevens CF (1991) Excitatory and inhibitory autaptic currents in isolated hippocampal neurons maintained in cell culture. Proc Natl Acad Sci USA 88: 7834-7838

Chiappalone M, Casagrande S, Tedesco M, Valtorta F, Baldelli P, Martinoia S, Benfenati F (2009) Opposite changes in glutamatergic and GABAergic transmission underlie the diffuse hyperexcitability of synapsin I-deficient cortical networks. Cereb Cortex 19, 1422-1439

Fassio A, Patry L, Congia S, Onofri F, Piton A, Gauthier J, Pozzi D, Messa M, Defranchi E, Fadda M, Corradi A, Baldelli P, Lapointe L, St-Onge J, Meloche C, Mottron L, Valtorta F, Khoa Nguyen D, Rouleau GA, Benfenati F, Cossette P (2011) SYN1 loss-of-function mutations in autism and partial epilepsy cause impaired synaptic function. Hum Mol Genet 20, 2297-2307

Feligioni M, Holman D, Haglerod C, Davanger S, Henley JM (2006) Ultrastructural localisation and differential agonist-induced regulation of AMPA and kainate receptors present at the presynaptic active zone and postsynaptic density. J Neurochem 99, 549-560

Huttner WB, Schiebler W, Greengard P, De Camilli P (1983) Synapsin I (protein I), a nerve terminal-specific phosphoprotein. III. Its association with synaptic vesicles studied in a highly purified synaptic vesicle preparation. J Cell Biol 96, 1374-1388

Kurt MA, Kafa MI, Dierssen M, Davies DC (2004) Deficits of neuronal density in CA1 and synaptic density in the dentate gyrus, CA3 and CA1, in a mouse model of Down syndrome. Brain Res 1022, 101-109

Leung A (1994) Fixation. In "Laboratory histopathology: a complete reference" (AE Woods and RC Ellis eds.) Churchill Livingstone, New York.

Pfaffl MW (2001) A new mathematical model for relative quantification in real-time RT-PCR. Nucleic Acids Res 29: e45

Phillips GR, Huang JK, Wang Y, Tanaka H, Shapiro L, Zhang W, Shan WS, Arndt K, Frank M, Gordon RE, Gawinowicz MA, Zhao Y, Colman DR (2001) The presynaptic particle web: ultrastructure, composition, dissolution, and reconstitution. Neuron 32, 63-77

Rosenmund C, Stevens CF (1996) Definition of the readily releasable pool of vesicles at hippocampal synapses. Neuron 16, 1197-1207

Schneggenburger R, Meyer AC, Neher E (1999) Released fraction and total size of a pool of immediately available transmitter quanta at a calyx synapse. Neuron 23, 399-409

Schneggenburger R, Sakaba T, Neher E (2002) Vesicle pools and short-term synaptic depression: lessons from a large synapse. Trends Neurosci 25, 206-212

Staley K (2015) Molecular mechanisms of epilepsy. Nature Neuroscience 18, 367-372

Vandesompele J, De Preter K, Pattyn F, Poppe B, Van Roy N, De Paepe A, Speleman F (2002) Accurate normalization of real-time quantitative RT-PCR data by geometric averaging of multiple internal control genes. Genome Biol 3: research0034.1–research0034.11

Yamaguchi K, Tanaka M, Mizoguchi A, Hirata Y, Ishizaki H, Kaneko K, Miyoshi J, Takai Y (2002) A GDP/GTP exchange protein for the Rab3 small G protein family up-regulates a postdocking step of synaptic exocytosis in central synapses. Proc Natl Acad Sci USA 99, 14536-14541
