# Supplementary material for: PRRT2 Is a Key Component of the Ca2+-Dependent Neurotransmitter Release Machinery
Source: Cell Rep. 2016 Mar 24;15(1):117–31. doi: 10.1016/j.celrep.2016.03.005 (PMC4826441; doi:10.1016/j.celrep.2016.03.005)
Supplement: Document S2. Article plus Supplemental Information [file mmc2.pdf]

Cell Reports

Article

PRRT2 Is a Key Component of the Ca^{2+}-Dependent Neurotransmitter Release Machinery

Graphical Abstract

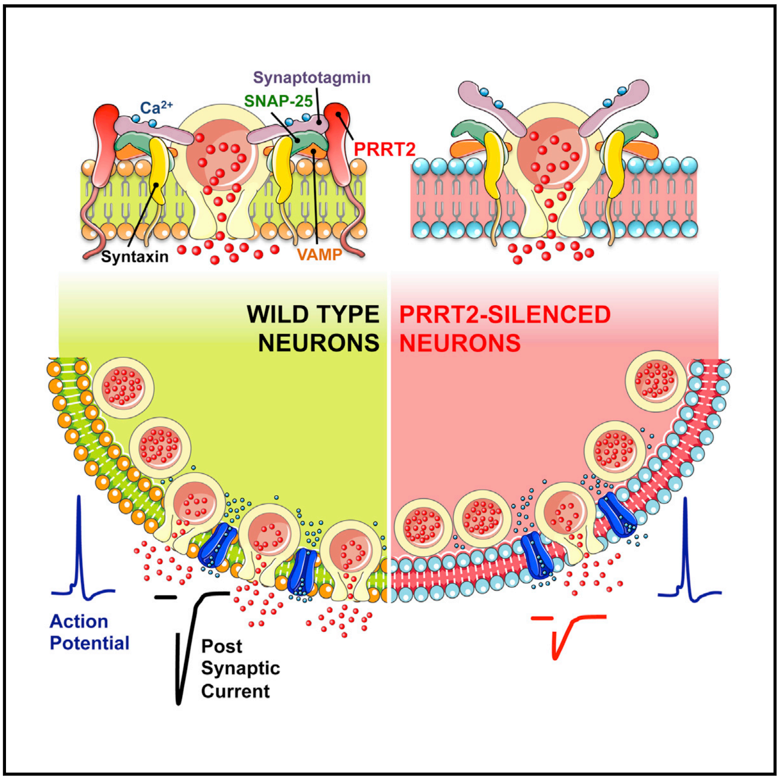

Highlights

- PRRT2 is a presynaptic protein
- PRRT2 is required for synchronous neurotransmitter release
- PRRT2 silencing decreases synaptic density and release probability
- PRRT2 interacts with the fast Ca^{2+} sensors synaptotagmin 1/2

Authors

Pierluigi Valente, Enrico Castroflorio, Pia Rossi, ..., Pietro Baldelli, Anna Corradi, Fabio Benfenati

Correspondence

fabio.benfenati@iit.it

In Brief

Valente et al. show that PRRT2, a single causative gene for a group of paroxysmal neurological diseases, is a key component of regulated exocytosis. Silencing PRRT2 dramatically impairs neurotransmitter release by markedly reducing release probability. PRRT2 interacts with the fast Ca^{2+} sensors synaptotagmin 1/2 and endows the SNARE complex with Ca^{2+} sensitivity.

Valente et al., 2016, Cell Reports *15*, 117–131
April 5, 2016 ©2016 The Authors
http://dx.doi.org/10.1016/j.celrep.2016.03.005

Cell Reports
Article

OPEN ACCESS
CellPress

PRRT2 Is a Key Component of the Ca^{2+}-Dependent Neurotransmitter Release Machinery

Pierluigi Valente,[1] Enrico Castroflorio,[1,2] Pia Rossi,[1] Manuela Fadda,[1] Bruno Sterlini,[1,2] Romina Ines Cervigni,[3] Cosimo Prestigio,[1,2] Silvia Giovedì,[1] Franco Onofri,[1] Elisa Mura,[3] Fabrizia C. Guarnieri,[3] Antonella Marte,[1] Marta Orlando,[2] Federico Zara,[4] Anna Fassio,[1,2] Flavia Valtorta,[3] Pietro Baldelli,[1,2] Anna Corradi,[1,5] and Fabio Benfenati[1,2,5,*]
[1]Department of Experimental Medicine, University of Genova, Viale Benedetto XV, 3, 16132 Genova, Italy
[2]Center for Synaptic Neuroscience and Technology, Istituto Italiano di Tecnologia, Largo Rosanna Benzi 10, 16132 Genova, Italy
[3]San Raffaele Scientific Institute and Vita Salute University, Via Olgettina 58, 20132 Milano, Italy
[4]Department of Neuroscience, Istituto Giannina Gaslini, Via Gerolamo Gaslini, 5, 16148 Genova, Italy
[5]Co-senior author
*Correspondence: fabio.benfenati@iit.it
http://dx.doi.org/10.1016/j.celrep.2016.03.005

SUMMARY

Heterozygous mutations in proline-rich transmembrane protein 2 (PRRT2) underlie a group of paroxysmal disorders, including epilepsy, kinesigenic dyskinesia, and migraine. Most of the mutations lead to impaired PRRT2 expression, suggesting that loss of PRRT2 function may contribute to pathogenesis. We show that PRRT2 is enriched in presynaptic terminals and that its silencing decreases the number of synapses and increases the number of docked synaptic vesicles at rest. PRRT2-silenced neurons exhibit a severe impairment of synchronous release, attributable to a sharp decrease in release probability and Ca^{2+} sensitivity and associated with a marked increase of the asynchronous/synchronous release ratio. PRRT2 interacts with the synaptic proteins SNAP-25 and synaptotagmin 1/2. The results indicate that PRRT2 is intimately connected with the Ca^{2+}-sensing machinery and that it plays an important role in the final steps of neurotransmitter release.

INTRODUCTION

Over the last 4 years, several studies have identified an array of heterozygous nonsense, frameshift, and missense mutations in the gene encoding proline-rich transmembrane protein 2 (PRRT2) in a large number of cases affected by different paroxysmal disorders such as benign familial infantile seizures, infantile convulsion choreoathetosis, migraine, hemiplegic migraine, paroxysmal kinesigenic dyskinesia/choreoathetosis, benign familial infantile seizures/epilepsy, and episodic ataxia (Chen et al., 2011; Lee et al., 2012; for review, see Ebrahimi-Fakhari et al., 2015). The astonishing pleiotropy of the phenotypic expression of PRRT2 mutations points to an overlap in the pathogenic pathways and to a very important role of this gene in regulating synaptic transmission and network activity.

The PRRT2 gene is located in human chromosome 16 and consists of four exons encoding a poorly characterized protein of 340 amino acids. Most mutations identified in PRRT2 are phenotypically highly penetrant and cause truncation of the protein because of nonsense or frameshifts mutations that result in mRNA degradation by nonsense-mediated mRNA decay or degradation of the protein (Wu et al., 2014), suggesting a loss-of-function mechanism of action.

PRRT2 mRNA has been shown to be almost exclusively expressed in neurons in the cortex, hippocampus, basal ganglia, and cerebellum (Chen et al., 2011; Lee et al., 2012; Heron et al., 2012; Trabzuni et al., 2011), which are all regions putatively involved in the pathogenesis of the PRRT2-linked diseases. The functional role of this protein is totally unknown. A yeast two-hybrid screen highlighted a potential interaction of PRRT2 with synaptosomal-associated protein 25 kDa (SNAP-25), one of the presynaptic soluble N-ethylmaleimide sensitive factor (NSF) attachment protein receptor (SNARE) proteins triggering fusion of synaptic vesicles (SVs) (Stelzl et al., 2005; Lee et al., 2012). Recently, PRRT2 has also been found among proteins associated with α-amino-3-hydroxy-5-methyl-4-isoxazolepropionic acid (AMPA)-type glutamate receptors by a high-resolution proteomics study (Schwenk et al., 2014), and its mutation was associated with increased levels of extracellular glutamate (Li et al., 2015).

To investigate the role of PRRT2 in presynaptic physiology, we silenced PRRT2 expression in primary neurons using RNA interference. PRRT2 knockdown caused a marked impairment in synaptic transmission because of a decrease in the density of synaptic connections and a sharp reduction in release probability. Both effects were fully reversible upon re-expression of shRNA-resistant PPRT2. In addition to SNAP-25, the search for PRRT2 partners identified the fast Ca^{2+} sensors synaptotagmins 1/2, responsible for triggering synchronous neurotransmitter release. The results indicate that PRRT2 is intimately connected with the Ca^{2+}-sensing machinery of neurotransmitter release.

RESULTS

PRRT2 Is a Presynaptic Protein Developmentally Expressed during Synaptogenesis

We evaluated the regional expression of PRRT2 protein in various areas of the adult mouse brain and the developmental

This is an open access article under the CC BY license (http://creativecommons.org/licenses/by/4.0/).

OPEN ACCESS
CellPress

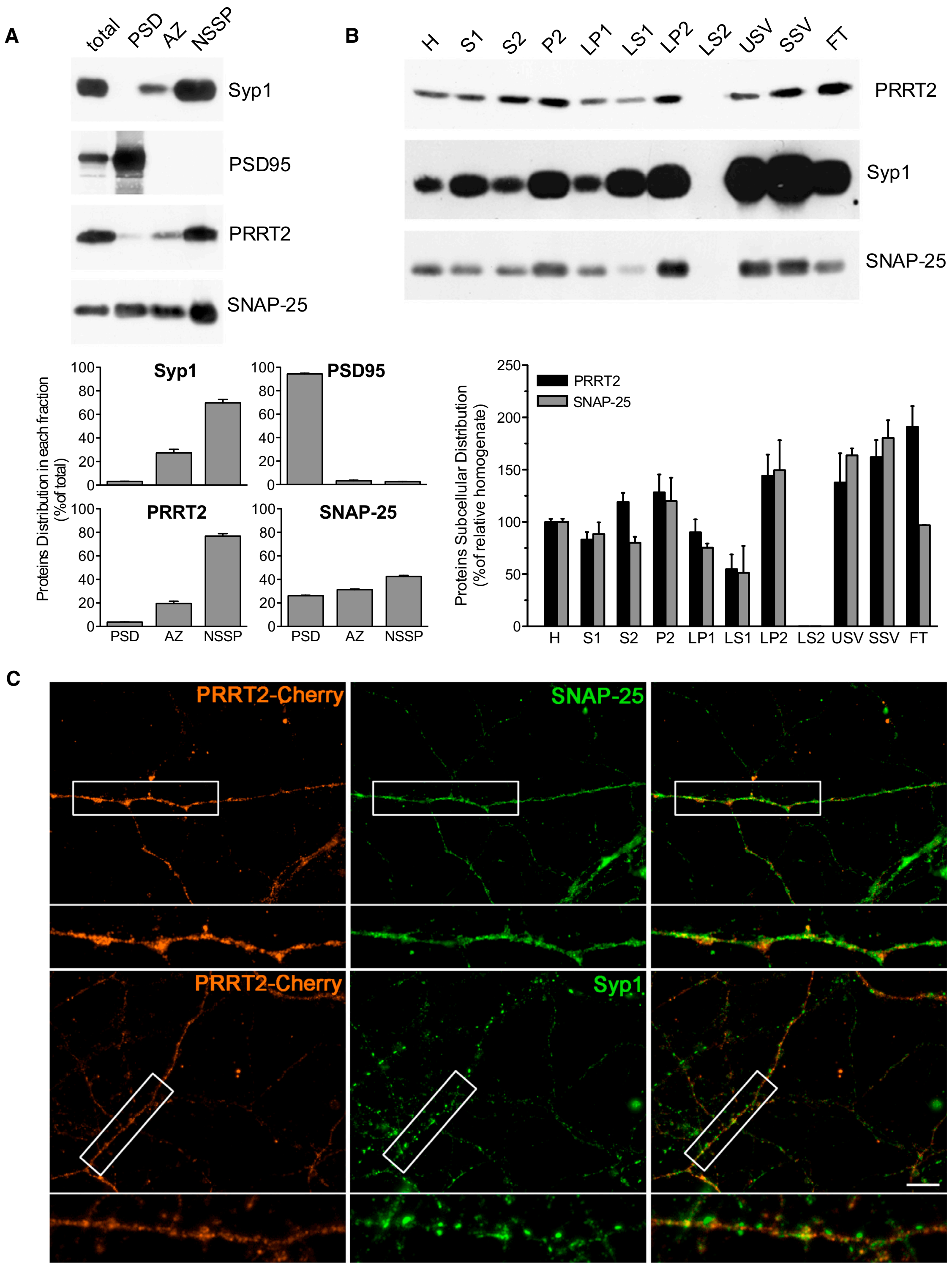

Figure 1. PRRT2 Is Localized at the Presynaptic Level

(A) Ultrafractionation of brain synaptosomes. Purified synaptosomes from adult mouse brain were subjected to ultrasynaptic fractionation to separate the AZ, PSD, and NSSP made by extrinsic and integral membrane proteins of the nerve terminal. Aliquots of total synaptosomes and of each ultrasynaptic fraction (10–30 μg) were probed with antibodies against PRRT2, SNAP-25, and protein markers to validate ultrasynaptic compartments such as synaptophysin-1 (Syp1) and PSD95 (top). Immunoblots were quantified by densitometric analysis of the fluorograms, and the values are expressed in mean (± SEM) percentages of the

(legend continued on next page)

expression profile of PRRT2 in both mouse brain and primary neuronal cultures (Figure S1). PRRT2 expression was prominent throughout the adult brain, with the highest levels in the cerebellum, basal ganglia, and neocortex (Figure S1A). In the cerebral cortex and hippocampus, the PRRT2 gene was already expressed at early postnatal stages, and its expression increased to reach a plateau at 1 month of life, a period of intense synapse formation and rearrangement (Figures S1B and S1C). The same pattern was reproduced in primary hippocampal and cortical neurons, where PRRT2 expression, already discernible at early stages, was greatly enhanced between 10 and 21 days in vitro (DIVs), a temporal window of intense synaptogenesis (Figures S1B and S1D). In contrast, PRRT2 expression was almost undetectable in primary astroglial cultures (data not shown), consistent with the neuron-specific expression of PRRT2.

To verify the subcellular distribution of PRRT2 at the synapse, we fractionated purified synaptosomes to isolate synaptic junctions, subsequently separated into active zone (AZ), postsynaptic density (PSD), and a fraction including extrinsic and integral membrane proteins associated with the presynaptic area (nonsynaptic synaptosomal protein [NSSP]) (Phillips et al., 2001). PRRT2 was enriched in the NSSP fraction, like SNAP-25 and synaptophysin. Although the protein appeared to be substantially absent from the PSD, low levels were associated with the AZ fraction (Figure 1A). We further analyzed PRRT2 enrichment along a subcellular fractionation procedure, yielding highly purified SVs (Huttner et al., 1983). Interestingly, we found that SNAP-25 and PRRT2 had a similar subcellular distribution and associated with both SVs and presynaptic membrane fractions (LP2 and FT) (Figure 1B). These data confirm that PRRT2 can be considered a bona fide presynaptic protein and suggest that it could undergo trafficking between the presynaptic membrane and the SV compartments, as described previously for SNAP-25 (Walch-Solimena et al., 1995). The lack of reliable anti-PRRT2 antibodies for immunocytochemistry did not allow us to unambiguously visualize the endogenously expressed protein in tissue and primary neurons. Therefore, to test for the localization of the protein in neuronal cells, 10-DIVs hippocampal neurons were transduced with lentiviruses encoding for PRRT2-mCherry and immunostained at 15 DIVs. In agreement with the biochemical experiments, PRRT2 colocalized with SNAP-25 and synaptophysin, confirming its targeting to the synapse (Figure 1C).

To silence PRRT2 expression, five short hairpin RNAs (shRNAs) were designed on the basis of the open reading frame of the mouse PRRT2 transcript and inserted into lentiviral vectors expressing either TurboGFP (tGFP) or the mCherry cytosolic reporter for cell identification. Four shRNAs were effective in knocking down PRRT2 expression to very low levels in HeLa cells, whereas control scrambled shRNAs (Scr) were virtually ineffective (data not shown). The two most active PRRT2-shRNAs (Sh1 and Sh4) and two control (Scr) shRNAs were chosen for the subsequent studies. Because the tested shRNAs were directed against the PRRT2 coding sequence, we generated shRNA-resistant PRRT2 clones fused to mCherry for rescuing the PRRT2 knockdown. Infection of low-density mouse cortical neurons with the appropriate lentiviral vectors resulted in an effective silencing of PRRT2 and in the expression of the shRNA-resistant version of PRRT2 overriding the RNA interference (Figures S1E–S1I).

Knockdown of PRRT2 in Low-Density Primary Neurons Decreases the Density of Synaptic Contacts and Alters the Synaptic Ultrastructure

Because PRRT2 expression paralleled synapse formation and rearrangement, we studied whether its knockdown (KD) was associated with changes in synaptic density or assembly. The effects of PRRT2 KD on synaptic connectivity in primary hippocampal neurons were analyzed by counting synapse density in mature (14 DIVs) neurons infected with either Sh4 or Sh1 at 7 DIVs (Figures 2A and 2B; Figures S2A and S2B). Synaptic boutons were unambiguously identified by counting puncta with a size of <1 μm double-positive for pairs of pre/postsynaptic proteins such as Bassoon/Homer1 (Figures 2A and 2B; Figure S2A) or synaptophysin/PSD95 (Figure S2B). Strikingly, the density of excitatory synapses along dendrites was decreased by ≈50% in neurons silenced for PRRT2 with either shRNA, an effect that was fully rescued by the expression of shRNA-resistant PRRT2 (Figure 2B; Figure S2B).

Next we ascertained whether the ultrastructure of the rarified synapses was affected by PRRT2 downregulation (Figures 2C–2I). Conventional transmission electron microscopy on excitatory synapses from primary neurons infected as above confirmed the decrease in synapse density in the absence of major changes in the terminal ultrastructure (Figures 2C and 2D). To analyze in greater detail the morphology of the PRRT2 KD terminals, we performed serial sectioning followed by 3D reconstruction (Figure 2E). This analysis detected a 2-fold increase in the number of docked SVs in PRRT2-silenced synapses in the context of an unaltered total number of SVs (Figures 2F and 2G). Consistent with this finding, the frequency distribution of the distance of SVs from the AZ showed an accumulation of SVs at shorter distances in PRRT2 KD terminals (Figure 2H). Interestingly, the average diameter of SVs in silenced synapses

total amount (bottom). The partition of PSD95, Syp1, and SNAP-25 in the corresponding fractions is shown. Note that PRRT2 preferentially partitioned in the NSSP fraction, similarly to Syp1 and SNAP-25.

(B) Subcellular distribution of endogenous PRRT2 in neurons. Forebrain fractions obtained at various stages of SV purification were analyzed by western blotting using antibodies to PRRT2, SNAP-25, and Syp1 (top). H, homogenate; S1, post-nuclear supernatant; S2, supernatant of P2; P2, crude synaptosomes; LP1, crude synaptic plasma membranes; LS1, supernatant of LP1; LP2, crude synaptic vesicles; LS2, synaptosol; USV, highly purified synaptic vesicles; SSV, salt-treated highly purified synaptic vesicles; FT, flowthrough fraction containing small presynaptic membranes. Immunoblots were quantified as in (A), and the value of each subcellular fraction is expressed in percentage of homogenate as means ± SEM (bottom).

(C) Localization of PRRT2 in mature neurons. Primary hippocampal neurons transduced at 10 DIVs with PRRT2-mCherry (red) were subjected to immunostaining at 15 DIVs with antibodies to PRRT2, SNAP-25, and Syp1. PRRT2 immunoreactivity (red) largely overlapped with the staining of the two presynaptic proteins in axonal and nerve terminal areas. Scale bar, 10 μm.

See also Figure S1.

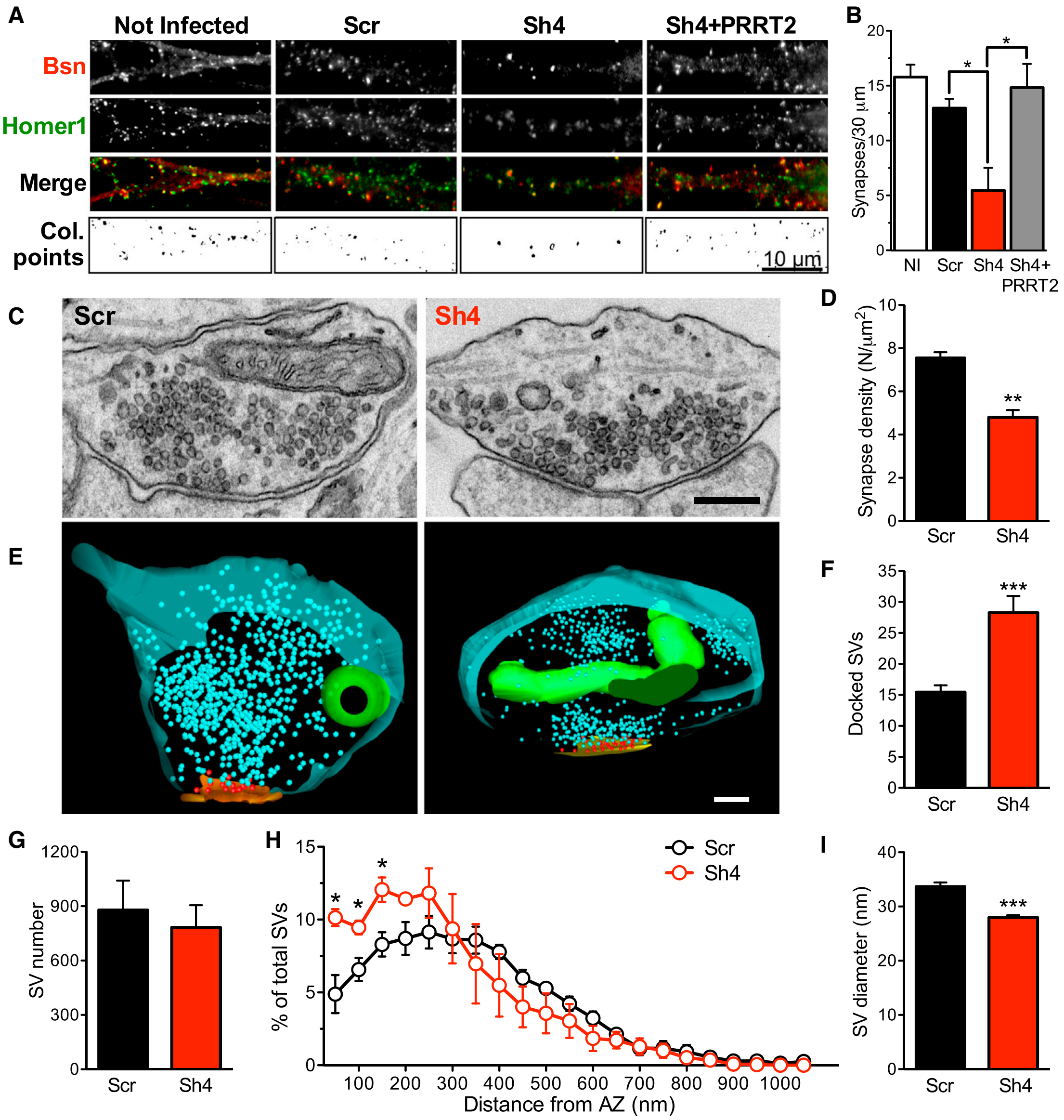

Figure 2. PRRT2 Knockdown Decreases Synapse Density and Increases Docked SVs in Low-Density Hippocampal Neurons

(A) Representative images of dendrites of hippocampal neurons infected at 7 DIVs with Scr, Sh4, and Sh4 + Sh4-resistant PRRT2 (Sh4+PRRT2) or left uninfected and analyzed at 14 DIVs. Synaptic boutons were identified by double immunostaining for Bassoon (Bsn, red) and Homer1 (green). The colocalization panels (Col. points) highlight the double-positive puncta (black) corresponding to synapses. Scale bar, 10 μm.

(B) Quantitative analysis of synaptic puncta counted on 30-μm dendrite tracts starting from the cell body in neurons treated as in (A). Data are means ± SEM from three independent experiments, each carried out in duplicate. Five dendrites for each neuron, from at least ten neurons for each sample, were counted. *$p < 0.05$, one-way ANOVA/Bonferroni's multiple comparison test. NI, not infected.

(C). Conventional TEM analysis of nerve terminals from PRRT2 KD hippocampal neurons revealed an increase in docked SVs and a preservation of the total SVs with respect to control neurons. Shown are representative TEM images of nerve terminals from neurons transduced with either Scr or Sh4 at 7 DIVs and fixed/processed at 14 DIVs. Scale bar, 200 μm.

(D) Quantitative TEM analysis of the synaptic density from serial ultrathin sections. The volume density of symmetric and asymmetric synapses was calculated from the 2D count of synaptic profiles in sections from Sh4- (red bars) and Scr-treated (black bars) neurons and is expressed as mean (± SEM) number of synapses per square micrometer.

(E). 3D reconstructions of synaptic terminals from serial ultrathin sections confirm the increase in the number of docked SVs in low-density neuronal cultures. Shown are representative 3D reconstructions from 60-nm-thick serial sections obtained from Scr-treated (left) and PRRT2 KD (right) synapses. Total SVs and SVs physically docked at the AZ are depicted as blue and red spheres, respectively. The AZ and mitochondria are shown in yellow and green, respectively. Scale bar, 200 nm.

(F and G) Morphometric analysis of three-dimensionally reconstructed synapses. PRRT2 KD synapses (red bars) displayed an increased number of AZ-docked SVs (F) and a preserved total number of SVs (G) with respect to Scr-treated synapses (black bars).

(legend continued on next page)

was significantly smaller than the average diameter of SVs in control terminals (Figure 2I), with an approximate one-third reduction in SV volume.

We next assessed whether these ultrastructural changes were altered by neuronal activity. To this aim, we imaged Scr- and Sh4-infected neurons immediately after a field train stimulation (300 action potentials [APs] at 10 Hz) and 20 min later (Figure S2C). Immediately after the stimulation, the density of SVs was found to be decreased to a similar extent in both Scr- and Sh4-treated synapses, but, in PRRT2 KD synapses, recovery was delayed. Interestingly, the increase in the density of docked SVs observed at rest in PRRT2 KD neurons was not evident immediately after the stimulation but reappeared after 20 min of recovery, suggesting a possible role of PRRT2 as a negative regulator of SV docking at rest.

Knockdown of PRRT2 in Low-Density Primary Neurons Affects SV Recycling without Modifying the Size of the Readily Releasable Pool

To couple morphological with functional analysis of SV trafficking and monitor the SV exo-endocytotic cycle at single synapses, we employed synaptophysin-pHluorin (SypHy), a pH-sensitive fluorescent sensor exquisitely designed to monitor the SV exo-endocytosis cycle at single synapses (Figure S3A; Miesenböck et al., 1998; Kavalali and Jorgensen, 2014). SypHy was co-expressed in neurons transduced with either the Scr or Sh4 variant expressing mCherry as a spectrally non-overlapping reporter. To evaluate the effect of PRRT2 KD on SV recycling evoked by electrical activity, field stimulation protocols triggering APs were applied (400 APs at 20 Hz) that allowed us to estimate the extent and kinetics of exocytosis and endocytosis (Verstegen et al., 2014). Experiments were performed at 17 DIVs (5 days after infection), and mCherry/SypHy double-positive puncta on axonal processes were selected for analysis (Figure 3A). We found that the SypHy fluorescence increase evoked by tetanic stimulation was significantly lower in PRRT2-silenced boutons in the absence of noticeable effects on the kinetics of "post-stimulus" endocytosis (Figures 3B and 3C). When the kinetics of release during stimulation were evaluated and fitted according to a mono-exponential model, a 2-fold increase in the time constant was observed in PRRT2-silenced neurons (Figure 3C). To exclude interference from endocytosis and vesicle re-acidification, we repeated the analysis in the presence of the proton pump inhibitor bafilomycin, which allows extracting the contribution of exocytosis to the rising phase of the fluorescence response to stimulation (Figure S3A). Interestingly, the significant decrease in the fluorescence peak and slowdown of the release kinetics were virtually abolished, indicating that the extent and dynamics of exocytosis, as measured by the SypHy assay, were not markedly altered by PRRT2 silencing (Figure 3C). This also suggests that PRRT2 silencing may affect during-stimulus endocytosis and/or SV re-acidification.

To clarify whether PRRT2-silenced synapses are impaired in the priming/fusion steps of release, we stimulated neurons with 40 APs at 20 Hz to measure the readily releasable pool (RRP) of SVs and assess whether the increased docked SVs observed by electron microscopy at rest were functional. We found that both the fluorescence peak at the end of the 2-s stimulation (representing the RRP size) and the kinetics of fluorescence return to basal levels at the end of the stimulation (describing the post-stimulus endocytosis) were virtually unaffected by PRRT2 silencing, indicating that the priming process produces a normal pool of releasable SVs (Figures 3D and 3E).

To confirm the abovementioned data, we used an alternative, spectrally distinct reporter of exo-endocytosis, Synaptobrevin2-Orange (Syb2O, a pH-sensitive pHluorin based on the SV protein VAMP2/synaptobrevin; Ramirez et al., 2012) co-expressed with the green tGFP reporter version of either Sh4- or Sh1-expressing vectors. The effects of PRRT2 silencing observed with the SypHy assay were fully reproduced using the Syb2O reporter and an alternative shRNA to knockdown PRRT2 (Figure S3B–S3E).

PRRT2 Silencing Reversibly Knocks Down Spontaneous and Evoked Synaptic Transmission in Autaptic Neurons

To analyze how PRRT2 KD affects the function and strength of excitatory and inhibitory synapses and avoid the confounding effects of non-transduced neurons, we used autaptic hippocampal neurons. We first checked whether autaptic neurons exhibited the same morphological and ultrastructural changes observed in low-density cultures. VGLUT1-immunoreactive puncta representing autapses were significantly less numerous in tGFP-positive PRRT2-silenced autaptic neurons, confirming the results described in low-density cultures (Figures S4A and S4B). Except for a decrease in total SVs (that was not apparent in low-density neurons), silenced autaptic neurons exhibited a phenotype identical to low-density neurons, with a significant increase in the density of SVs docked at the AZ (Figures S4C and S4D), a shift in the frequency distribution of the distance of SVs from the AZ with accumulation of SVs at shorter distances (Figure S4E), and a decrease in SV diameter (Figure S4F).

Having confirmed the morphological effects of PRRT2 silencing, we proceeded to analyze spontaneous and evoked synaptic transmission in transduced autaptic neurons identified based on tGFP/mCherry fluorescence (Figure 4A). Miniature excitatory postsynaptic currents (mEPSCs) were continuously recorded at the soma of voltage-clamped neurons held at

(H) Spatial distribution of SVs in nerve terminals of Scr-treated (black symbols) and Sh4-treated (red symbols) neurons. The mean (± SEM) number of SVs located within successive 50-nm shells from the AZ and normalized by the total SV content of each terminal is given as mean ± SEM as a function of the distance from the AZ.

(I) Morphometric analysis of SV diameter. PRRT2 KD synapses (red bars) displayed a smaller SV size with respect to Scr-treated synapses (black bars).

Nerve terminal areas (0.689 ± 0.044 μm^2 and 0.741 ± 0.035 μm^2 for Scr- and PRRT2 sh-RNA-infected neurons, respectively) and AZ lengths (0.302 ± 0.023 μm and 0.347 ± 0.013 μm for Scr- and PRRT2 sh-RNA-infected neurons, respectively) were similar in the two experimental groups (140 and 150 synapses for Scr and PRRT2 shRNA-infected neurons, respectively, from three independent preparations). *$p < 0.05$, **$p < 0.01$, ***$p < 0.001$, unpaired Student's t test (E, G, and H) and Kolmogorov-Smirnov test (F). See also Figure S2.

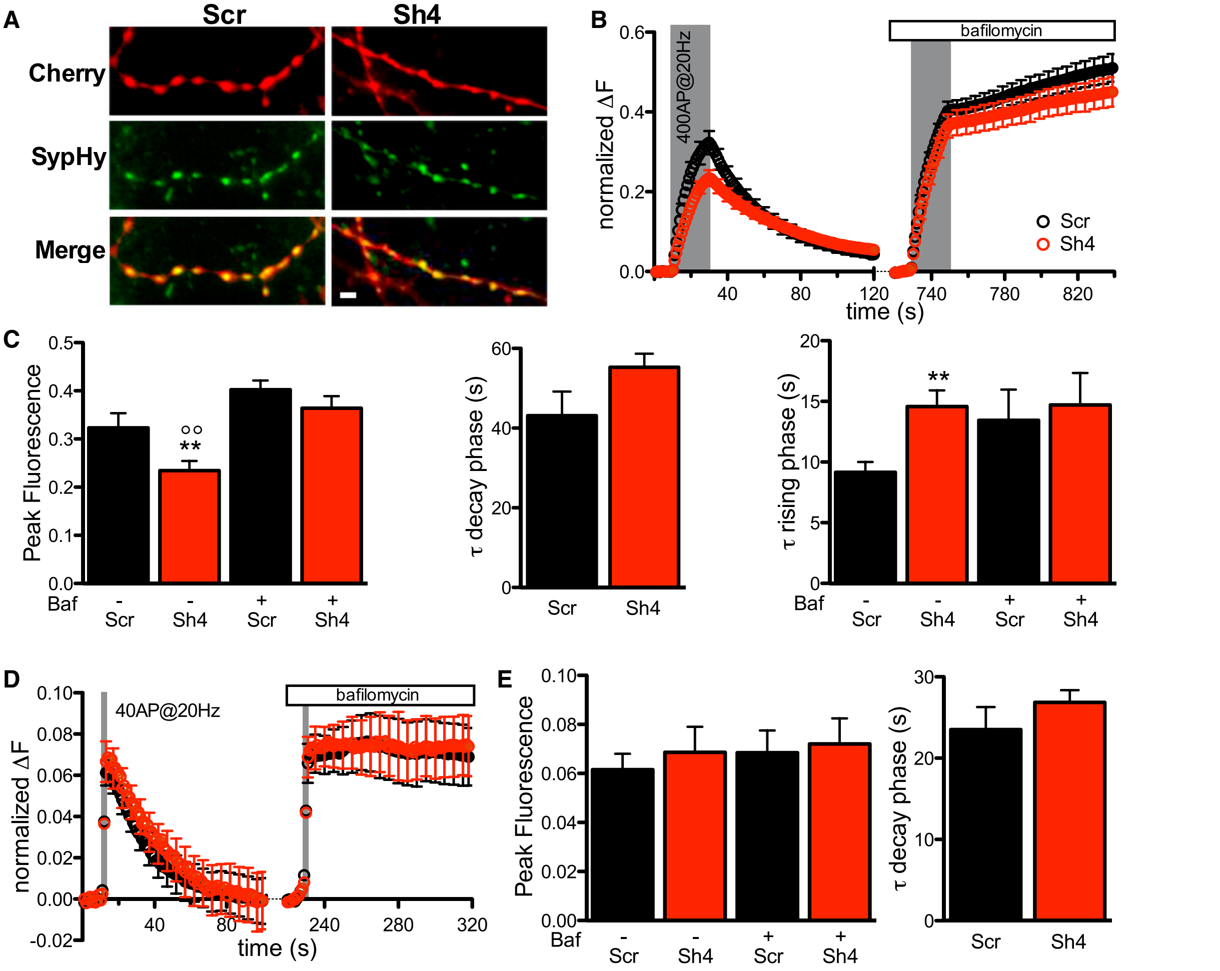

Figure 3. PRRT2 Knockdown Slows Down SV Cycling in Response to Sustained High-Frequency Stimulation without Altering Exocytosis

(A). Representative experimental field showing low-density hippocampal neurons co-expressing the reporter of SV exo-endocytosis synaptophysin-pHluorin (Syphy) and either Sh4 or Scr (mCherry). The merged images show the colocalization of the two markers at synaptic puncta. Scale bar, 10 μm.

(B). Ensemble averaged traces of SypHy fluorescence from PRRT2 KD synapses (Sh4, red trace) and control synapses (Scr, black trace) recorded in response to electrical field stimulations at 20 Hz for 20 s (shaded area) in the absence or presence of bafilomycin (1 μM). Data are normalized to the maximum fluorescence intensity reached under NH_4Cl perfusion (normalized ΔF).

(C) Left: quantitative evaluation of the SV pool released during the stimulation and plotted as peak fluorescence reached at the end of the stimulation in the absence (left) or presence (right) of bafilomycin (Baf). Center: quantitative evaluation of the kinetics of SV endocytosis (τ decay phase) by single exponential fitting of the post-stimulus curves for Sh4-treated (red) and Scr-treated (black) synapses. Right: kinetics of SV release under stimulation at 20 Hz (left) and relative time constant (τ) of the rising phase in the absence or presence of bafilomycin determined by exponential fitting of individual experiments. The rate of SV release was greatly reduced in PRRT2 KD synapses (Sh4, red trace) compared with control synapses (Scr, black trace), but the rate of pure exocytosis determined by blocking reacidification with bafilomycin was not significantly altered by PRRT2 KD. Data are expressed as mean ± SEM (shown every five time points in B) from 11 (Sh4, 340 synapses) and 13 (Scr, 380 synapses) experiments from three different preparations. **$p < 0.01$ versus Scr; °°$p < 0.01$ versus Sh4+Baf; one-way ANOVA/ Bonferroni's or Kruskal-Wallis/Dunn's tests.

(D and E) Evaluation of the readily releasable pool in PRRT2-silenced neurons.

(D) Ensemble-averaged SypHy traces from PRRT2 KD synapses (Sh4, red trace) and control synapses (Scr, black trace) recorded in response to electrical field stimulations with 40 APs at 20 Hz (shaded area) in the absence or presence of bafilomycin. Fluorescence values were normalized to the maximum fluorescence intensity reached under NH_4Cl perfusion (normalized ΔF).

(E) Left: peak fluorescence at the end of the stimulus recorded in the absence or presence of bafilomycin. Right: time constant of endocytosis (τ decay phase) evaluated by fitting the fluorescence decay after stimulation by a single exponential function for Sh4-treated (red) and Scr-treated (black) synapses.

Data are expressed as mean ± SEM (shown every five time points in D) from nine (Sh4, 260 synapses) and ten (Scr, 240 synapses) experiments from three different preparations. See also Figure S3.

−70 mV (V_h) in the presence of tetrodotoxin (TTX) to block spontaneous APs (Figure 4B; Chiappalone et al., 2009). Strikingly, the mEPSC frequency fell 3- to 4-fold in PRRT2-silenced cultures (Figure 4C), an extent much larger than expected from the decrease in synapse density observed in PRRT2-silenced autaptic excitatory neurons. Moreover, the mEPSC amplitude,

OPEN ACCESS
CellPress

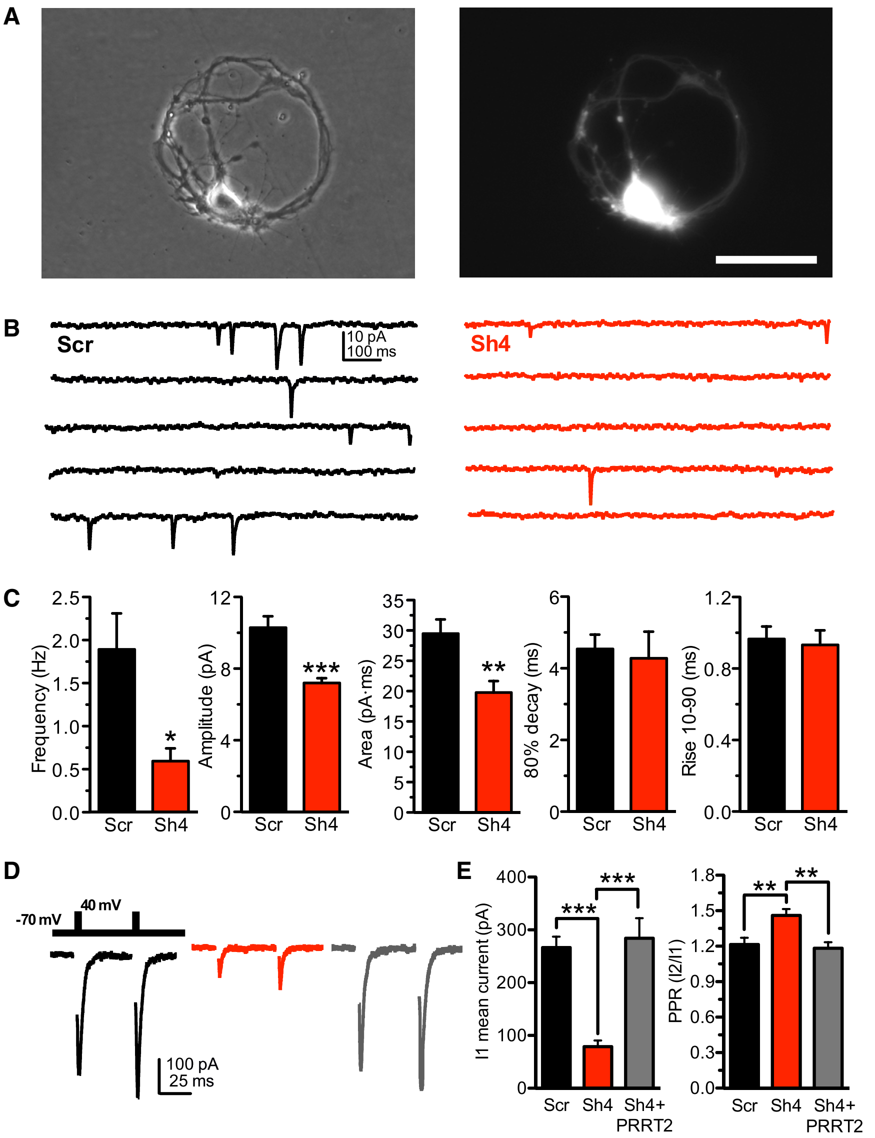

Figure 4. PRRT2 Knockdown Dramatically Decreases Spontaneous and Evoked Synaptic Transmission in Hippocampal Autaptic Neurons

(A) Phase-contrast micrographs of a typical hippocampal autaptic neuron (left). The positivity of the same cell to infection with the Sh4 construct was probed by fluorescence imaging of the tGFP reporter (right). Scale bar, 100 μm.

(B) Representative recording traces of mEPSCs from PRRT2-KD synapses (Sh4, red traces) and control synapses (Scr, black trace).

(C) Analysis of mEPSCs. From left to right, shown are mean ± SEM frequency, amplitude, charge, 80% decay time, and 10%–90% rise time of mEPSCs calculated for PRRT2 KD (n = 11, red bars) and control (n = 15, black bars) neurons. All values were obtained from 100–1000 events recorded from each cell in 5-min recordings.

(D) Representative eEPSCs recorded in excitatory autaptic neurons infected with Scr (black trace/bar, n = 39), PRRT2-Sh4 (red trace/bar, n = 32), or PRRT2-Sh4 + Sh-resistant PRRT2 (gray trace/bar, n = 21). eEPSCs were elicited by clamping the cell under study at −70 mV and stimulating it with two voltage steps to +40 mV lasting 0.5 ms at an interstimulus interval of 50 ms. The paired-pulse stimulation was applied every 10 s (inset).

(E) Decrease of eEPSC amplitude and increase of PPR by PRRT2 KD and rescue of the PRRT2 KD phenotype by expression of Sh-resistant PRRT2. Shown is the quantitative analysis of the eEPSC amplitude evoked by the first pulse (I1, left) and PPR (I2/I1, right) recorded in excitatory autaptic neurons treated as described in (D). A complete rescue of the EPSC amplitude and PPR was observed in silenced neurons expressing Sh-resistant PRRT2.

In all graphed currents, stimulation artifacts were blanked for clarity. Data are expressed as means ± SEM from the indicated numbers of cells recorded from at least three independent cell culture preparations. *$p < 0.05$, **$p < 0.01$, ***$p < 0.001$, unpaired Student's t test or Mann-Whitney's U test (C); Kruskal-Wallis/Dunn's multiple comparison test (E). See also Figures S4 and S5.

reflecting quantal size, was also decreased by approximately 30% in the absence of significant changes in the current waveform, as indicated by the analysis of decay and rise times (Figure 4C). A reduction of approximately the same extent was also observed in the mEPSC area (unitary charge). Such a change may be attributable to the corresponding reduction in the average SV volume, although concomitant postsynaptic effects cannot be excluded.

For the analysis of evoked transmission, transduced autaptic neurons, identified as excitatory or inhibitory based on the kinetics of postsynaptic currents (PSCs) and sensitivity to specific blockers of AMPA or γ-aminobutyric acid receptor type A ($GABA_A$) receptors, were stimulated with paired stimuli at an interpulse interval of 50 ms to evaluate the paired-pulse ratio (PPR), an indirect measure of release probability (Fioravante and Regehr, 2011). Strikingly, the vast majority of excitatory neurons knocked down for PRRT2 by either Sh4 or Sh1 and analyzed over time in culture displayed a dramatic decrease of EPSC amplitude in response to single stimuli with a strong increase in paired-pulse facilitation (Figures 4D and 4E; Figures S5A–S5D), suggesting a presynaptic defect affecting release probability. The effect of PRRT2 silencing was reversible; co-infection of the neurons with Sh-resistant PRRT2 was able to achieve a virtually complete return of current amplitude and PPR to the levels of Scr-infected neurons (Figures 4D and 4E). The magnitude of the synaptic impairment increased with development, suggesting an absent or delayed maturation of the release machinery in PRRT2-silenced neurons (Figure S5B).

A closely similar phenotype was observed in PRRT2-silenced inhibitory neurons, which represent 7%–10% of the total autaptic neuronal population under our culture conditions (Figure S5E).

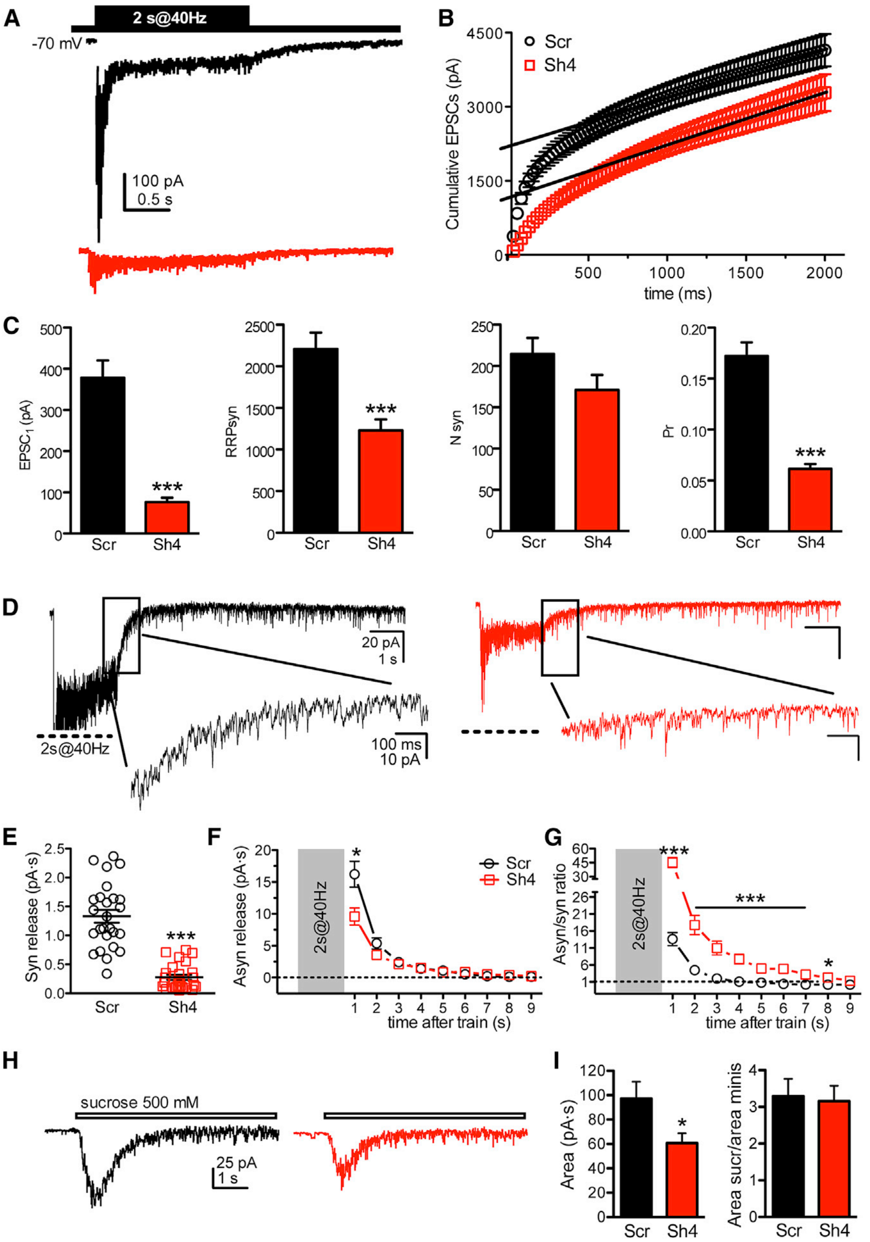

Figure 5. PRRT2 Knockdown Strongly Decreases Synchronous but Not Asynchronous Release in Excitatory Autapses

(A) Representative recordings of eEPSCs evoked by high-frequency stimulation (train of 80 stimuli at 40 Hz, inset) in autaptic hippocampal neurons infected with Scr (black trace) or PRRT2-Sh4 (red trace). Stimulation artifacts were blanked for clarity.

(B) Profiles of the mean cumulative amplitude of eEPSCs for neurons infected with Scr (black trace, n = 38) or PRRT2-Sh4 (red trace, n = 34) constructs. Data points in the 1- to 2-s range were fitted by a linear regression and back-extrapolated to time 0 (solid lines) to estimate the RRP_{syn}.

(C) Results of the quantal analysis. From left to right, shown are the mean ± SEM amplitude of the first eEPSC, RRP_{syn} size, number of RRP_{syn} quanta, and initial probability of release (Pr) estimated in neurons infected with Scr (n = 38, black bars) or PRRT2-Sh4 (n = 34, red bars).

(D) Representative traces showing asynchronous release evoked by a tetanic stimulation of 2 s at 40 Hz in autaptic neurons transduced with either Scr (black traces) or PRRT2-Sh4 (red traces). Traces in the insets are shown in an expanded timescale.

(E–G) Comparative analysis of synchronous and asynchronous release.

(E) Individual values and mean (± SEM) of the synchronous charge released from Scr-infected (black, n = 26) and Sh4-infected (red, n = 26) neurons stimulated by one AP 10 s before the train. Synchronous charge was estimated by measuring the area of the eEPSC in a time window of 5 ms following its activation.

(F) Time course of asynchronous release calculated by measuring the area of the spontaneous EPSCs evoked by tetanic stimulation. The area was calculated in nine time windows, each lasting 1 s.

(G) Time course of the synchronous/asynchronous ratio calculated for the nine time windows from the data shown in (C) and (B), respectively.

(H and I) Estimation of RRP_{total} by hypertonic stimulation of autaptic neurons.

(H) Representative EPSC responses activated by 6 s of hypertonic stimulation (horizontal bar) recorded in autaptic neurons transduced with either scrambled shRNA (black) or PRRT2-Sh4 (red).

(I) Mean values (± SEM) of the RRP_{total} charge transfer obtained by hypertonic stimulation and number of RRP_{total} quanta obtained in either Scr (black, n = 18) or PRRT2-Sh4 (red, n = 15) neurons by dividing the RRP_{total} charge by the unitary mEPSC charge.

Data are shown as means ± SEM. *p < 0.05, **p < 0.01, ***p < 0.001, unpaired Student's t test or Mann-Whitney *U* test.

The impairment of the evoked inhibitory current appeared to be even more dramatic than that of excitatory neurons recorded in the same plates (inset in Figure S5F). In analogy to what was observed for excitatory transmission, the strong impairment in inhibitory transmission was associated with an increase in PPR, which made paired-pulse depression milder (Figure S5G). The results suggest that PRRT2 downregulation affects the Ca^{2+} coupling between action potential and exocytosis by decreasing the probability of release (Pr), thereby rendering facilitation more intense in excitatory synapses or depression milder in inhibitory synapses.

PRRT2 Knockdown Decreases the Probability of Synchronous Release and Greatly Increases the Asynchronous/Synchronous Release Ratio

To identify the quantal parameters of release affected by PRRT2 silencing, we performed cumulative evoked EPSC (eEPSC) amplitude analysis in control and silenced autaptic excitatory neurons. When neurons were challenged with a train of 2 s at 40 Hz, a significant depression of eEPSCs became apparent during the train in both control and silenced neurons, irrespective of the amplitude of the first current in the train (Figure 5A). Accordingly, the cumulative profile showed a rapid rise followed

by a slower linear increase, reflecting the equilibrium between the depletion and constant replenishment of the RRP (Figure 5B).

Interestingly, the 4- to 5-fold reduction of the current amplitude in silenced neurons was contributed by a sharp (about −65%) decrease in the initial Pr together with a significant decrease of the RRP_{syn} total current. However, when the readily releasable pool current of synchronous release (RRP_{syn}) was normalized by the unitary current (Q_{av}, corresponding to the average amplitude of autaptic mEPSCs), the number of readily releasable SVs (N_{syn}) was not significantly different between control and PRRT2-silenced neurons (Figure 5C), suggesting, in agreement with the results of the SypHy assay, a normal priming process generating the RRP in PRRT2 KD neurons. Therefore, the strong impairment in evoked synchronous release is likely to involve changes in the Ca^{2+} dependence of release that can occur at the level of either Ca^{2+} entry or Ca^{2+} sensing.

Because asynchronous release has a distinct Ca^{2+} sensitivity, we measured both the synchronous and the delayed asynchronous release as the transferred charge in response to trains of 2 s at 40 Hz (Medrihan et al., 2013; Figure 5D). Although the synchronous charge, measured as the response to a single AP 10 s before the train, was dramatically decreased in PRRT2-silenced neurons (Figure 5E), both control and silenced autapses showed a robust asynchronous release after the train that slowly returned to baseline within 10 s (Figures 5F and 5G). In PRRT2-silenced autapses, asynchronous release was significantly decreased only immediately after the end of the train, but thereafter it was indistinguishable from that of control autapses (Figure 5F). When the asynchronous/synchronous charge ratio was calculated (Figure 5G), silenced neurons had a percent asynchronous versus synchronous release that was much larger than that of control neurons. This is compatible with the normal released SV pool observed with the SypHy assay (as shown previously shown for Syt1 knockout neurons; Nishiki and Augustine, 2004) and further suggests that the main impairment in the absence of PRRT2 resides in Ca^{2+} sensing commanding fast, synchronous release and not in the subsequent asynchronous SV fusion process.

Given the sharp decrease in synchronous release and the substantial preservation of asynchronous release, we assessed the RRP size of the total release (RRP_{total}) with a fully independent procedure; i.e., by exposing synapses to hypertonic sucrose stimulation (Figure 5H). Consistent with the data reported above, during the transient part of the high sucrose-induced eEPSC, a significant decrease of the charge transfer, a reliable measure of RRP_{total}, was observed in PRRT2-silenced neurons (Figure 5I). However, when RRP_{total} was normalized by the unitary SV charge, the resulting quantal content of RRP_{total} was comparable between control and PRRT2-silenced neurons (Figure 5I). The substantial preservation of the total pool of readily releasable SVs indicates that the same total SV pool is shared by synchronous and asynchronous release and further suggests that SV priming is not affected by PRRT2 silencing.

PRRT2 Knockdown Decreases the Ca^{2+} Sensitivity of Synchronous Release and Alters Short-Term Plasticity

Given the sharp impairment in spontaneous and evoked release, we investigated the Ca^{2+} sensitivity of release by increasing the extracellular Ca^{2+} concentration or by challenging synchronous and asynchronous release with the cell-permeable Ca^{2+} chelator EGTA-acetoxymethyl (EGTA-AM). In Scr-treated neurons, the increase in external Ca^{2+} from 2 to 4 mM enhanced the frequency and amplitude of mEPSCs (Figure 6A) as well as the amplitude of eEPSCs while decreasing PPR (Figure 6B), consistent with a heightened release probability. Strikingly, PRRT2-silenced neurons were totally insensitive to the increase in extracellular Ca^{2+} as far as both spontaneous and evoked release were concerned.

Known as a slow chelator, EGTA should be more active in inhibiting delayed asynchronous release rather than fast, synchronous release triggered by Ca^{2+} increases in the nanodomains adjacent to Ca^{2+} channels. However, in control hippocampal autapses, EGTA-AM application significantly reduced both synchronous and asynchronous release to approximately the same extent, indicating that fast release occurs within microdomains generated by the spatial overlap of local Ca^{2+} influxes through multiple adjacent Ca^{2+} channels (Wang and Augustine, 2014). Interestingly, the effect of EGTA was fully occluded by PRRT2 KD, consistent with an effect on the Ca^{2+} dependency of release (Figure 6C).

An alternative way to assess Ca^{2+} sensitivity of release is to investigate facilitation and potentiation, which are short-term plasticity phenomena depending on an activity-dependent Ca^{2+} build-up in the nerve terminal (Figure S6; Fioravante and Regehr, 2011). Excitatory autaptic neurons were stimulated with 2-s trains at frequencies ranging from 10–40 Hz, and the development of facilitation/depression was analyzed over time. PRRT2-silenced neurons were characterized by a massive and intense facilitation whose peak was significantly delayed with respect to that of control neurons (Figures S6A and S6C). Interestingly, the amplitude of the last EPSC of the train became indistinguishable from the last EPSC of control neurons that was largely depressed at 40 Hz of stimulation (Figure S6B). When the EPSC amplitude during the train was normalized with the amplitude of the first EPSC, PRRT2-silenced neurons exhibited an intense and prolonged facilitation without experiencing any depression during 10- or 20-Hz trains (Figure S6C). Although the pronounced facilitation and the increased PPR are the likely consequences of the low initial Pr, the delay in the facilitation peak further suggests an impaired Ca^{2+}-sensing ability. Post-tetanic potentiation (PTP) is a form of short-term synaptic plasticity contributed by increases in both RRP and Pr induced by the train stimulation (Fioravante and Regehr, 2011; Valente et al., 2012). A short high-frequency stimulation (HFS) (2 s at 40 Hz; Figure S6D) was used to evoke PTP, and a single stimulus applied every 10 s was used to monitor the plastic changes in eEPSC amplitude. PRRT2-silenced neurons expressed an approximately 30% smaller potentiation than Scr-treated control neurons (Figures S6E and S6F), consistent with an inability of PRRT2-silenced neurons to increase the Pr because of an impairment in the Ca^{2+} sensing process.

PRRT2 Interacts with SNARE Proteins and Synaptotagmins 1 and 2

The physiological phenotype of PRRT2 silencing prompted the search for possible presynaptic PRRT2 interactors implicated in

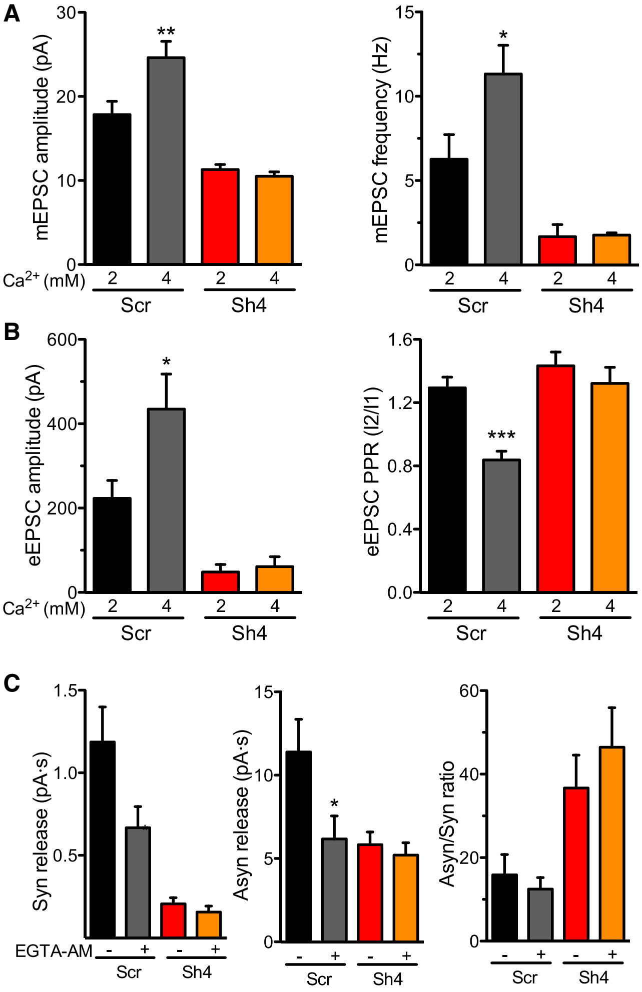

Figure 6. The Ca^{2+} Sensitivity of Spontaneous and Synchronous Release Is Decreased by PRRT2 Knockdown

(A) Amplitude (left) and frequency (right) of mEPSCs recorded in neurons transduced with Scr (black/gray bars, n = 9/7) and Sh4 (red/orange bars, n = 10/8) as a function of the extracellular Ca^{2+} concentration (2 mM Ca^{2+}, black/red bars; 4 mM Ca^{2+}, gray/orange bars).

(B) eEPSC amplitude evoked by the first pulse (I1, left) and PPR (I2/I1, right) recorded in neurons transduced with Scr (black/gray bars, n = 7) or PRRT2-Sh4 (red/orange bars, n = 6). The graph bars represent the mean of the EPSC amplitude and PPR recorded in individual cells before (black/red bars) and after (gray/orange bars) the increase of the extracellular Ca^{2+} concentration to 4 mM.

(C) Effects of EGTA-AM on synchronous and asynchronous release. Left: synchronous charge released from Scr-treated (black/gray bars, n = 12) and Sh4-treated (red/orange bars, n = 11) neurons stimulated by one AP 10 s before the train. Synchronous charge was estimated by measuring the area of the eEPSC in a time window of 5 ms following its activation. Center: asynchronous charge induced by a tetanic stimulation of 2 s at 40 Hz and calculated by measuring the area of the spontaneous EPSCs in the time window of 1 s after the end of the train. Right: synchronous/asynchronous ratio calculated for the nine time windows from the data shown in (C) and (B), respectively.

Data are means ± SEM. *p < 0.05, **p < 0.01, ***p < 0.001, one-way ANOVA/Bonferroni's multiple comparison test. See also Figure S6.

the post-docking events of release. We focused on presynaptic proteins that, when inactivated in knockout (KO) mice, cause a strong impairment in evoked release, including the SNARE proteins VAMP2, SNAP-25, and syntaxin 1, the fast Ca^{2+} sensors synaptotagmins (Syt) 1/2, Rab3 interacting molecule (RIM) binding protein, complexins 1/2, and Munc-18 (Südhof, 2013). FLAG-tagged PRRT2 or a FLAG-tagged control purified in eukaryotic cells was used to pull down PRRT2 interactors from synaptosomal extracts of the mouse brain. A significant pull-down of Syt2 as well as a somewhat weaker interaction with SNAP-25 and its SNARE partner VAMP2 were observed in the absence of significant interactions with the other proteins tested (Figure 7A). Although the interaction of PRRT2 with SNAP-25 has been reported previously (Stelzl et al., 2005; Lee et al., 2012; Li et al., 2015), the interaction with Syt2, a fast Ca^{2+} sensor highly homologous to Syt1 (Südhof, 2013), is of great interest in light of the physiological phenotype associated with PRRT2 silencing. Under our conditions, Syt2 was expressed by both low-density and autaptic hippocampal neurons, although to a much lesser extent and with a more restricted pattern than the major isoform Syt1 (Figure S7A), consistent with previous reports (Pang et al., 2006a; Fox and Sanes, 2007; Kerr et al., 2008).

In view of the high homology of Syt2 with Syt1, and to explain the wide-range synaptic phenotype of PRRT2 knockdown, we further considered the possibility of an interaction of PRRT2 with Syt1 that might have escaped the conventional pull-down assay. Co-immunoprecipitation experiments from brain extracts with anti-Syt1 and anti-Syt2 antibodies showed that PRRT2 indeed associates with both Syt1 and Syt2 (Figure 7B). Because the lack of an interactor may affect the lifetime of its partner, we investigated whether the levels of Syt1 and/or Syt2 in low-density hippocampal cultures were affected by PRRT2 silencing. Both Syt isoforms were decreased in PRRT2 KD neurons. However, although the levels of Syt2 were decreased to the same extent as those of other synaptic proteins, likely expressing the decrease in synapse density, Syt1 expression levels were significantly smaller (Figure S7B).

In support of the hypothesis that the wide-range synaptic phenotype of PRRT2 KD is attributable to the interaction with either or both Syt1/Syt2, we tried to rescue the impairment in the evoked synchronous release by overexpressing Syt2 in PRRT2 KD autaptic neurons (Figure S7C). Expression of Syt2 induced a significant rescue of PRRT2 KD-induced impairment in eEPSC amplitude, although its magnitude was smaller than the complete rescue obtained with Sh-resistant PRRT2 (Figure 7C).

DISCUSSION

The dominant effect of PRRT2 mutations associated with diverse paroxysmal disorders points to an important role of the protein in neuronal activity. Because most of the mutations display a loss-of-function pathogenic mechanism of action, we used an acute silencing approach through RNA interference to dissect out the physiological role of the protein at the synaptic level.

Presynaptic Actions of PRRT2

Putative interactions with the SNARE protein SNAP-25 and the GluA1 subunit of the AMPA-type glutamate receptor complex have been suggested on the basis of proteomic studies (Stelzl et al., 2005; Schwenk et al., 2014). These interactions were subsequently demonstrated using recombinant PRRT2 (Lee et al., 2012; Li et al., 2015), opening the question of whether PRRT2 acts at the presynaptic level, postsynaptic level, or both. In this work, we provide evidence for a strong involvement of PRRT2 in presynaptic function. PRRT2 is increasingly expressed during synaptogenesis and, in mature synapses, codistributes with presynaptic markers such as synaptophysin or SNAP-25. Consistently, PRRT2 silencing in primary neurons decreases the density of synaptic connections and alters the nerve terminal ultrastructure. Stronger evidence for a presynaptic action comes from the effects of PRRT2 silencing on synaptic transmission. Indeed, in the absence of PRRT2, a downregulation of neurotransmitter release occurs with a dramatic impairment in spontaneous and synchronous release. The major traits of the PRRT2 KD phenotype (namely, the decreased frequency of mEPSCs, the impairment of evoked currents in both excitatory and inhibitory synapses, the increase in PPR, the selective drop in the initial Pr, the changes in short-term plasticity responses such as facilitation, depression, or PTP that are expressed predominantly at presynaptic level, as well as the ultrastructural changes testifying to an accumulation of SVs in the proximity of the release sites at rest) all strongly indicate that PRRT2 is an important presynaptic component of the release machinery. SypHy imaging experiments also uncovered an additional effect of PRRT2 silencing on SV recycling and/or acidification that confirms the presynaptic site of action of PRRT2.

PRRT2 and Synapse Morphology

One major effect of acute PRRT2 silencing is a decrease in the density of synaptic connections. The decreased number of synaptic contacts is more likely attributable to a developmental effect of PRRT2 whose expression parallels the period of synaptic formation and remodeling, although plastic pruning of downregulated synapses cannot be ruled out. The decreased density of synaptic connections was not accompanied by dramatic changes in the synaptic ultrastructure, except for an increase in the density of docked SVs at rest that is relieved by activity, suggesting that PRRT2 negatively controls SV docking under resting conditions, and a smaller SV volume that parallels the decrease in quantum size observed in the analysis of miniature synaptic currents. Because a pool of PRRT2 is associated with SVs, it is tempting to speculate that it may participate in the control of the integrity, size, and curvature of these organelles. Further studies are needed to dissect the molecular mechanisms of this effect.

PRRT2 and the Quantal Properties of Release

Quantal analysis has shown that the quantum size is decreased, the RRP of SVs is preserved, and the probability of spontaneous and evoked release is severely decreased in the absence of PRRT2. Although the decrease in quantum size is of the same magnitude as the reduction in SV volume, several considerations point to a fundamental role of PRRT2 in the machinery for synchronous release; namely, the decrease in the frequency of spontaneous release was much larger than the decrease in synapse density, the probability of evoked release was dramatically decreased, and modifications of nerve terminal Ca^{2+} levels revealed a defective Ca^{2+} secretion coupling.

These results suggest that a defect in the progression of SVs through the post-docking steps of release exists in PRRT2-silenced synapses. Three independent evaluations prove that the RRP size is preserved and that priming is not markedly altered. The effects on Pr and on the efficiency of Ca^{2+}-dependent fusion would suggest that the defect of PRRT2 silencing resides in the Ca^{2+}-sensing apparatus and/or in the fusion machinery. However, the persistence of asynchronous release indicates that the fusion mechanism per se is not disabled in the absence of PRRT2 and that an acute defect in coupling the fast Ca^{2+} influx to exocytosis is likely to be involved.

PRRT2: Component of the Fusion Machinery or Ca^{2+}-Sensing Device?

To test for known interactors of PRRT2, we screened an array of major presynaptic proteins whose constitutive knockout strongly impairs evoked transmission (see Südhof, 2013, for a review). Besides confirming the interaction with SNAP-25, we found that PRRT2 also associates with VAMP2 and Syt1/2.

The interactions with the SNARE proteins SNAP-25 and VAMP2 would suggest that PRRT2 acts as a catalyst in the hemifusion/fusion processes, although the relative preservation of asynchronous release argues against a major impairment in SNARE function and fusion. On the other hand, the dramatic drop in evoked synchronous release, which was insensitive to manipulations of the intraterminal Ca^{2+} levels, and the delayed peak of facilitation point to a defective excitation-secretion coupling in which either Ca^{2+} entry or Ca^{2+} sensing could be affected.

The interactions with Syt1/Syt2 open the possibility that PRRT2 participates in the regulation of the Ca^{2+}-sensing apparatus for fast synchronous release. Such interactions may involve the large N-terminal region of PRRT2 that we have shown recently to have an intracellular topology (Rossi et al., 2016). Together with Syt9, Syt1 and Syt2 are the synaptotagmins that mediate fast Ca^{2+}-triggered SV exocytosis (Xu et al., 2007; Pang and Südhof, 2010; Kochubey et al., 2011). Syt1 and Syt2 are highly homologous, form hetero-oligomers on the SV membrane (Osborne et al., 1999), and are thought to act in much the same way. Knockout of either Syt abolishes Ca^{2+}-triggered synchronous release, and the two Syts appear to be interchangeable to some extent in rescuing their knockout phenotype (Xu et al., 2007; Nagy et al., 2006).

Deletion of Syt1 in neurons revealed that the protein is essential for mediating fast and synchronous fusion events in response to isolated APs but is not required for spontaneous fusion (that is enhanced) and slow asynchronous fusion (Geppert et al., 1994). At the mouse neuromuscular junction, where Syt1 and Syt2 are co-expressed, genetic deletion of Syt2 generates a strikingly similar phenotype to PRRT2 silencing with a dramatic reduction and slowdown of evoked release, decreased release probability, marked increase in facilitation, and asynchronous release during and after stimulus trains (Pang et al., 2006a). In the calyx of Held,

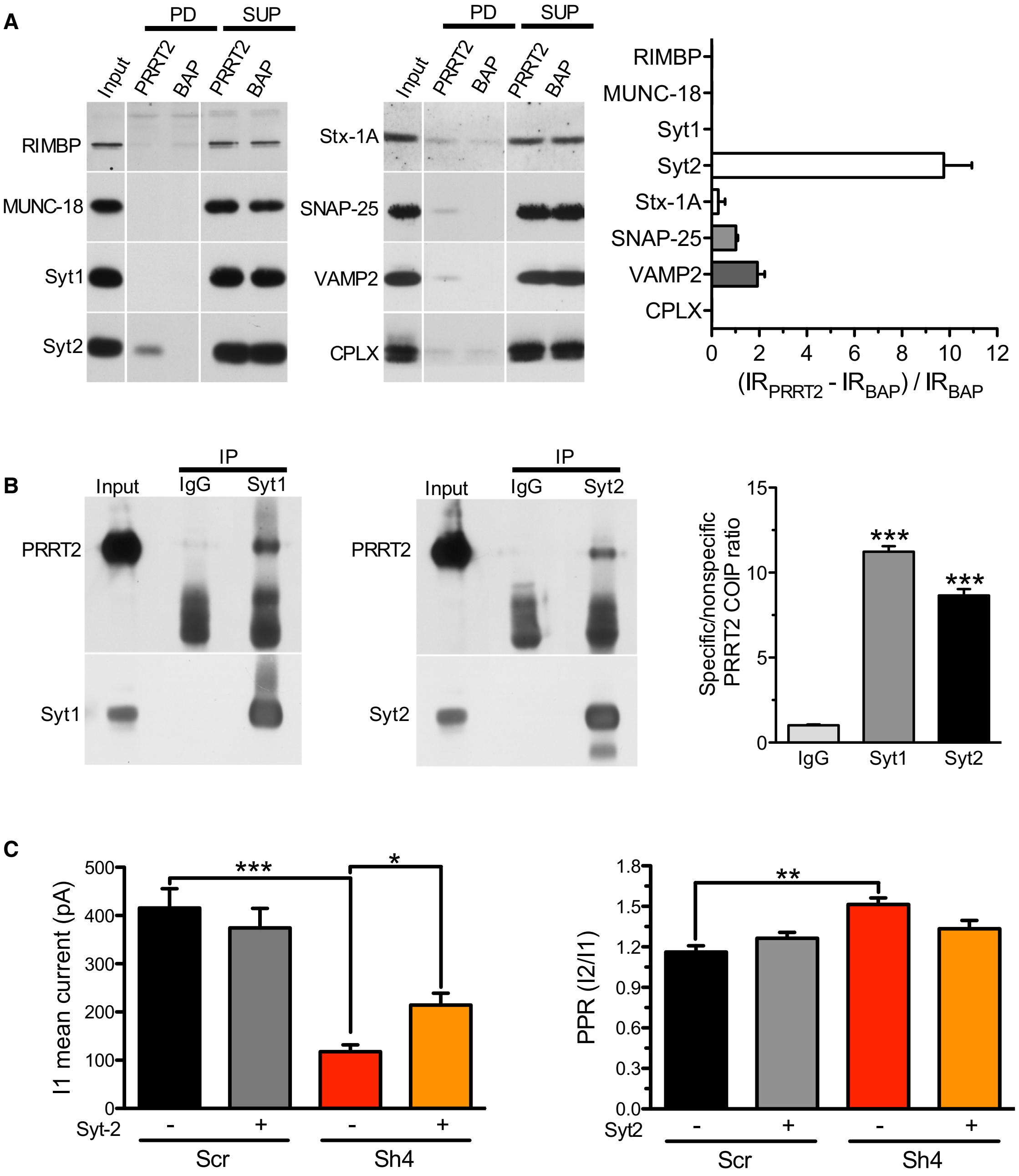

Figure 7. PRRT2 Interacts with the SNARE Complex Proteins and the Fast Ca^{2+} Sensors Synaptotagmin 1 and 2

(A) Pull-down assays with FLAG-tagged PRRT2. Left: FLAG-tagged PRRT2 (PRRT2) and bacterial alkaline phosphatase (BAP) vectors were expressed in HEK293T cells, purified by anti-FLAG M2 affinity gel, and incubated with synaptosome lysates. After pull-down (PD), pellets were solubilized and subjected to a western blotting assay together with aliquots of the starting material (input) and of the supernatants (SUP) using antibodies for a variety of potential presynaptic interactors of PRRT2, as shown. Vertical white lines in the blot indicate that the lanes were on the same gel but have been repositioned in the figure. Synaptosomal lysates incubated with FLAG-PRRT2 showed specific immunoreactivity for the SNARE complex proteins SNAP-25 and VAMP2 and for Syt2, which was not detected in FLAG-BAP precipitates. RIMBP, Rim binding protein; Syt, synaptotagmin; Stx-1A, syntaxin-1A, CPLX, complexins 1/2. Right: Immunoblots were quantified by densitometric analysis of the fluorograms obtained in the linear range of the emulsion response. The percent increase in the specific pull-down of the immunoreactivity (IR) by FLAG-PRRT2 with respect to the non-specific IR pulled down by the FLAG-BAP control ((IR$_{PRRT2}$ – IR$_{BAP}$) / IR$_{BAP}$) was calculated and is shown for each potential interactor as mean ± SEM (n = 3–4 independent PRRT2 preparations and subcellular fractionations).

(B) Co-immunoprecipitation of PRRT2 with Syt1 and Syt2. Detergent extracts of mouse brain were immunoprecipitated (IP) with monoclonal antibodies (mAbs) specific for Syt1 and Syt2 or with the respective control mouse immunoglobulin Gs (IgGs) as indicated. After electrophoretic separation of the immunocomplexes and western blotting, membranes were probed with anti-Syt1/anti-Syt2 antibodies to test the efficiency of the immunoprecipitation as well as with polyclonal

(legend continued on next page)

where Syt2 is the only fast Ca^{2+} sensor to be expressed, Syt2 KO deletes fast release but leaves asynchronous release unaffected (Sun et al., 2007). A similar dramatic reduction and slowdown of evoked release was present in a mouse strain bearing a destabilizing Syt2 mutation (Pang et al., 2006b) and exhibiting ataxia, one of the manifestations of PRRT2 mutations in patients.

Because it is known that spontaneous, synchronous, and asynchronous release modalities rely on distinct ranges of Ca^{2+} concentrations and, potentially, on distinct Ca^{2+} sensors, it is tempting to speculate that the interaction of PRRT2 with Syt1/2 is the key to mechanistically explain the strong and selective impairment of synchronous release with PRRT2 KD. The main phenotypic difference between Syt KOs and PRRT2 KD is observed in spontaneous release, which is increased in the former and impaired in the latter because of the concomitant decrease in synapse density. Our data suggest a model whereby PRRT2, by binding to SNAP-25 and Syt1/2, would increase the affinity of the SNARE complex for the fast Ca^{2+} sensor so that, in the absence of PRRT2, less SNARE complexes bind to the sensor, resulting in a dramatic decrease of the release probability. According to this model, the interactions between PRRT2 and Syt1/2 would be necessary to boost the Ca^{2+} sensitivity of evoked fast release, whereas they would be dispensable for the Syt-mediated clamping action on spontaneous release. Finally, the similar PRRT2-silencing phenotype found in excitatory and inhibitory autapses is fully consistent with an interaction of PRRT2 with both Syt1 and Syt2, which appear to be partly segregated in excitatory and inhibitory neurons, respectively (Fox and Sanes, 2007; Kerr et al., 2008; Pang et al., 2006a).

In conclusion, we demonstrate here that PRRT2 is an important regulator of the Ca^{2+}-sensing and SV fusion machinery. The results are consistent with a model in which PRRT2, by interacting with the fast Ca^{2+} sensors Syt1/2 and SNAREs, endows the SNARE complex with Ca^{2+} sensitivity. Further investigations will be needed to clarify the physiological regulation of these interactions and how their perturbation leads to brain paroxysmal activity.

EXPERIMENTAL PROCEDURES

Sprague-Dawley rats and C57BL/6J mice of either sex from Charles River Laboratories were used throughout. All experiments were carried out in accordance with the guidelines established by the European Communities Council (Directive 2010/63/EU of March 4, 2014) and were approved by the Italian Ministry of Health. The standard procedures for subcellular fractionation, western blotting, immunocytochemistry, pull-down and co-immunoprecipitation assays, real-time PCR, and cultures of low-density and autaptic hippocampal neurons are reported in detail in the Supplemental Experimental Procedures.

Transduction of Primary Neurons with PRRT2 shRNA and/or Sh-Resistant PRRT2

shRNAs (Scr, Sh1, and Sh4; Supplemental Experimental Procedures) were inserted into the pLKO.1-CMV-TurboGFP or pLKO.1-CMV-mCherry plasmid expressing either free tGFP or mCherry for the identification of the transfected cells. The PRRT2 Sh-resistant isoforms were subcloned into lentiviral vectors 743.pCCLsin.PPT.hPGK.GFP.Wpre (a gift from Dr. L. Naldini). Lentiviral vectors encoding Sh1, Sh4, or the Scr control RNAs were used to knock down the endogenous PPRT2 protein in hippocampal neurons. Neurons were infected by the addition of lentiviral vectors into cell medium at 6 DIVs. After 24 hr, the medium was removed and replaced with fresh and conditioned medium (1:1). A multiplicity of infection (MOI) of 5 of the lentivirus was used. For rescue experiments, neurons were co-transduced with lentiviruses expressing Sh1 or Sh4 shRNA and lentiviruses expressing the Sh1- or Sh4-resistant PRRT2-Cherry (5 MOI + 5 MOI) or with Scr sh-RNA (10 MOI). Syt2 overexpression was performed by co-infection of autaptic neurons with the Sh4 shRNA vector expressing soluble mCherry and with a lentiviral vector expressing Syt2 and soluble GFP (Biomatik). All physiological experiments were conducted between 10 and 15 DIVs.

Live Imaging of Exo-endocytosis

To couple the synaptic ultrastructure with functional analysis of SV trafficking, we employed Syphy and Syb2O, fluorescent probes exquisitely designed to monitor the SV exo-endocytosis cycle at single synapses (Figure S3A; Miesenböck et al., 1998; Ramirez et al., 2012). Optical recordings were performed at 17 DIV (5 days after transduction), as described in detail in the Supplemental Experimental Procedures.

Transmission Electron Microscopy

Autaptic hippocampal neurons and low-density cultures of hippocampal neurons were infected at 7–11 DIVs with either scramble shRNA or PRRT2 shRNAs and processed for transmission electron microscopy (TEM). The detailed procedures are reported in the Supplemental Experimental Procedures.

Patch-Clamp Recordings

Whole-cell patch-clamp recordings were made from autaptic neurons grown on microislands, as described previously (Valente et al., 2012). The detailed procedures are reported in the Supplemental Experimental Procedures.

Statistical Analysis

Data were expressed as means ± SEM for number of cells (n). Normal distribution was assessed using D'Agostino-Pearson's normality test. To compare two sample groups, Student's t test or Mann-Whitney *U* test was used. To compare more than two sample groups, ANOVA followed by Bonferroni's/Dunnett's post hoc tests or Kruskal-Wallis test followed by Dunn's post hoc test were used. Statistical analysis was carried out using OriginPro-8 (OriginLab) and Prism (GraphPad) softwares.

SUPPLEMENTAL INFORMATION

Supplemental Information includes Supplemental Discussion, Supplemental Experimental Procedures, and seven figures and can be found with this article online at http://dx.doi.org/10.1016/j.celrep.2016.03.005.

AUTHOR CONTRIBUTIONS

P.V. performed the electrophysiological experiments and analyzed the data. E.C. and M.O. performed and analyzed the ultrastructural experiments. P.R., B.S., and R.I.C. performed the cell biology studies. M.F. performed the live imaging experiments. F.O. and S.G. performed the biochemical experiments. B.S., A.M., and P.R. performed the affinity purification experiments. F.C.G. and E.M. ran the developmental studies and prepared the experimental tools. C.P. contributed to the electrophysiological experiments. A.F., P.B., and F.Z. supervised the electrophysiological and live imaging experiments, analyzed data, and contributed to writing the paper. F.B., F.V., and A.C. designed and

anti-PRRT2 antibodies. Left: a representative immunoblot is shown. Right: quantification of the PRRT2 immunoreactive signal in the immunoprecipitated samples, normalized to the binding to the mouse IgG control (means ± SEM, n = 3 independent experiments). Input, 10 μg total extract.

(C) Overexpression of Syt2 partially rescues the impairment in synchronous release of PRRT2 KD neurons. Autaptic hippocampal neurons were infected at 7 DIVs with Scr (n = 31), Sh4/mCherry (Sh4, n = 30), Syt2/GFP (Syt2, n = 28), or Sh4+Syt2 (n = 27) and recorded at 14 DIVs. The histograms show the means ± SEM of the eEPSC amplitude evoked by the first pulse (I1, left) and of the PPR (I2/I1, right). *$p < 0.05$, **$p < 0.01$; ***$p < 0.001$; Kruskal-Wallis/Dunn's multiple comparison test (I1); one-way ANOVA/Bonferroni's multiple comparison test (PPR). See also Figure S7.

supervised the research. F.B. and F.V. wrote the paper and supported the study.

ACKNOWLEDGMENTS

We thank Drs. Yongling Zhu and Charles F. Stevens (The Salk Institute) for the synaptophysin-pHluorin constructs; Ege T. Kavalali (Department of Neuroscience, University of Texas Southwestern Medical Center) for the SynaptobrevinOrange2 constructs; Thomas C. Südhof (Stanford University) for the synaptotagmin-2 construct; Luigi Naldini (Tiget) for lentiviral constructs and protocols; and Silvia Casagrande (Department of Experimental Medicine, University of Genova), Diego Moruzzo (Center for Synaptic Neuroscience and Technology, Istituto Italiano di Tecnologia), and Elena Monzani (San Raffaele Scientific Institute) for assistance with the preparation of primary cultures and viral constructs. This study was supported by research grants from the Italian Ministry of University and Research (PRIN 2010/11 to F.B., F.V., and F.O. and FIRB 2010 "Futuro in Ricerca" to S.G.), EU FP7 Integrating Project "Desire" (Grant Agreement 602531 to F.B. and F.Z.), and EU ITN "ECMED" (Grant Agreement 642881 to F.B.). The support of Telethon-Italy (Grant GGP13033 to F.B., F.V., and F.Z.), the CARIPLO Foundation (Grant 2013 0879 to F.V. and F.B.), and Italian Ministry of Health Ricerca Finalizzata 2013 (to F.V., F.B., and F.Z.) is also acknowledged.

Received: August 16, 2015
Revised: January 14, 2016
Accepted: February 25, 2016
Published: March 24, 2016

REFERENCES

Chen, W.J., Lin, Y., Xiong, Z.Q., Wei, W., Ni, W., Tan, G.H., Guo, S.L., He, J., Chen, Y.F., Zhang, Q.J., et al. (2011). Exome sequencing identifies truncating mutations in PRRT2 that cause paroxysmal kinesigenic dyskinesia. Nat. Genet. *43*, 1252–1255.

Chiappalone, M., Casagrande, S., Tedesco, M., Valtorta, F., Baldelli, P., Martinoia, S., and Benfenati, F. (2009). Opposite changes in glutamatergic and GABAergic transmission underlie the diffuse hyperexcitability of synapsin I-deficient cortical networks. Cereb. Cortex *19*, 1422–1439.

Ebrahimi-Fakhari, D., Saffari, A., Westenberger, A., and Klein, C. (2015). The evolving spectrum of PRRT2-associated paroxysmal diseases. Brain *138*, 3476–3495.

Fioravante, D., and Regehr, W.G. (2011). Short-term forms of presynaptic plasticity. Curr. Opin. Neurobiol. *21*, 269–274.

Fox, M.A., and Sanes, J.R. (2007). Synaptotagmin I and II are present in distinct subsets of central synapses. J. Comp. Neurol. *503*, 280–296.

Geppert, M., Goda, Y., Hammer, R.E., Li, C., Rosahl, T.W., Stevens, C.F., and Südhof, T.C. (1994). Synaptotagmin I: a major Ca^{2+} sensor for transmitter release at a central synapse. Cell *79*, 717–727.

Heron, S.E., Grinton, B.E., Kivity, S., Afawi, Z., Zuberi, S.M., Hughes, J.N., Pridmore, C., Hodgson, B.L., Iona, X., Sadleir, L.G., et al. (2012). PRRT2 mutations cause benign familial infantile epilepsy and infantile convulsions with choreoathetosis syndrome. Am. J. Hum. Genet. *90*, 152–160.

Huttner, W.B., Schiebler, W., Greengard, P., and De Camilli, P. (1983). Synapsin I (protein I), a nerve terminal-specific phosphoprotein. III. Its association with synaptic vesicles studied in a highly purified synaptic vesicle preparation. J. Cell Biol. *96*, 1374–1388.

Kavalali, E.T., and Jorgensen, E.M. (2014). Visualizing presynaptic function. Nat. Neurosci. *17*, 10–16.

Kerr, A.M., Reisinger, E., and Jonas, P. (2008). Differential dependence of phasic transmitter release on synaptotagmin 1 at GABAergic and glutamatergic hippocampal synapses. Proc. Natl. Acad. Sci. USA *105*, 15581–15586.

Kochubey, O., Lou, X., and Schneggenburger, R. (2011). Regulation of transmitter release by Ca(2+) and synaptotagmin: insights from a large CNS synapse. Trends Neurosci. *34*, 237–246.

Lee, H.Y., Huang, Y., Bruneau, N., Roll, P., Roberson, E.D., Hermann, M., Quinn, E., Maas, J., Edwards, R., Ashizawa, T., et al. (2012). Mutations in the gene PRRT2 cause paroxysmal kinesigenic dyskinesia with infantile convulsions. Cell Rep. *1*, 2–12.

Li, M., Niu, F., Zhu, X., Wu, X., Shen, N., Peng, X., and Liu, Y. (2015). PRRT2 Mutant Leads to Dysfunction of Glutamate Signaling. Int. J. Mol. Sci. *16*, 9134–9151.

Medrihan, L., Cesca, F., Raimondi, A., Lignani, G., Baldelli, P., and Benfenati, F. (2013). Synapsin II desynchronizes neurotransmitter release at inhibitory synapses by interacting with presynaptic calcium channels. Nat. Commun. *4*, 1512.

Miesenböck, G., De Angelis, D.A., and Rothman, J.E. (1998). Visualizing secretion and synaptic transmission with pH-sensitive green fluorescent proteins. Nature *394*, 192–195.

Nagy, G., Kim, J.H., Pang, Z.P., Matti, U., Rettig, J., Südhof, T.C., and Sørensen, J.B. (2006). Different effects on fast exocytosis induced by synaptotagmin 1 and 2 isoforms and abundance but not by phosphorylation. J. Neurosci. *26*, 632–643.

Nishiki, T., and Augustine, G.J. (2004). Dual roles of the C2B domain of synaptotagmin I in synchronizing Ca2+-dependent neurotransmitter release. J. Neurosci. *24*, 8542–8550.

Osborne, S.L., Herreros, J., Bastiaens, P.I., and Schiavo, G. (1999). Calcium-dependent oligomerization of synaptotagmins I and II. Synaptotagmins I and II are localized on the same synaptic vesicle and heterodimerize in the presence of calcium. J. Biol. Chem. *274*, 59–66.

Pang, Z.P., and Südhof, T.C. (2010). Cell biology of Ca^{2+}-triggered exocytosis. Curr. Opin. Cell Biol. *22*, 496–505.

Pang, Z.P., Melicoff, E., Padgett, D., Liu, Y., Teich, A.F., Dickey, B.F., Lin, W., Adachi, R., and Südhof, T.C. (2006a). Synaptotagmin-2 is essential for survival and contributes to Ca^{2+} triggering of neurotransmitter release in central and neuromuscular synapses. J. Neurosci. *26*, 13493–13504.

Pang, Z.P., Sun, J., Rizo, J., Maximov, A., and Südhof, T.C. (2006b). Genetic analysis of synaptotagmin 2 in spontaneous and Ca^{2+}-triggered neurotransmitter release. EMBO J. *25*, 2039–2050.

Phillips, G.R., Huang, J.K., Wang, Y., Tanaka, H., Shapiro, L., Zhang, W., Shan, W.S., Arndt, K., Frank, M., Gordon, R.E., et al. (2001). The presynaptic particle web: ultrastructure, composition, dissolution, and reconstitution. Neuron *32*, 63–77.

Ramirez, D.M., Khvotchev, M., Trauterman, B., and Kavalali, E.T. (2012). Vti1a identifies a vesicle pool that preferentially recycles at rest and maintains spontaneous neurotransmission. Neuron *73*, 121–134.

Rossi, P., Sterlini, B., Castroflorio, E., Marte, A., Onofri, F., Valtorta, F., Maragliano, L., Corradi, A., and Benfenati, F. (2016). Novel Topology of Proline-Rich Transmembrane Protein 2 (PRRT2): Hints for an Intracellular Function at the Synapse. J. Biol. Chem. *2016*, 21.

Schwenk, J., Baehrens, D., Haupt, A., Bildl, W., Boudkkazi, S., Roeper, J., Fakler, B., and Schulte, U. (2014). Regional diversity and developmental dynamics of the AMPA-receptor proteome in the mammalian brain. Neuron *84*, 41–54.

Stelzl, U., Worm, U., Lalowski, M., Haenig, C., Brembeck, F.H., Goehler, H., Stroedicke, M., Zenkner, M., Schoenherr, A., Koeppen, S., et al. (2005). A human protein-protein interaction network: a resource for annotating the proteome. Cell *122*, 957–968.

Südhof, T.C. (2013). Neurotransmitter release: the last millisecond in the life of a synaptic vesicle. Neuron *80*, 675–690.

Sun, J., Pang, Z.P., Qin, D., Fahim, A.T., Adachi, R., and Südhof, T.C. (2007). A dual-Ca^{2+}-sensor model for neurotransmitter release in a central synapse. Nature *450*, 676–682.

Trabzuni, D., Ryten, M., Walker, R., Smith, C., Imran, S., Ramasamy, A., Weale, M.E., and Hardy, J. (2011). Quality control parameters on a large dataset of regionally dissected human control brains for whole genome expression studies. J. Neurochem. *119*, 275–282.

Valente, P., Casagrande, S., Nieus, T., Verstegen, A.M., Valtorta, F., Benfenati, F., and Baldelli, P. (2012). Site-specific synapsin I phosphorylation participates in the expression of post-tetanic potentiation and its enhancement by BDNF. J. Neurosci. *32*, 5868–5879.

Verstegen, A.M., Tagliatti, E., Lignani, G., Marte, A., Stolero, T., Atias, M., Corradi, A., Valtorta, F., Gitler, D., Onofri, F., et al. (2014). Phosphorylation of synapsin I by cyclin-dependent kinase-5 sets the ratio between the resting and recycling pools of synaptic vesicles at hippocampal synapses. J. Neurosci. *34*, 7266–7280.

Walch-Solimena, C., Blasi, J., Edelmann, L., Chapman, E.R., von Mollard, G.F., and Jahn, R. (1995). The t-SNAREs syntaxin 1 and SNAP-25 are present on organelles that participate in synaptic vesicle recycling. J. Cell Biol. *128*, 637–645.

Wang, L.Y., and Augustine, G.J. (2014). Presynaptic nanodomains: a tale of two synapses. Front. Cell. Neurosci. *8*, 455.

Wu, L., Tang, H.D., Huang, X.J., Zheng, L., Liu, X.L., Wang, T., Wang, J.Y., Cao, L., and Chen, S.D. (2014). PRRT2 truncated mutations lead to nonsense-mediated mRNA decay in Paroxysmal Kinesigenic Dyskinesia. Parkinsonism Relat Disord. *20*, 1399–1404.

Xu, J., Mashimo, T., and Südhof, T.C. (2007). Synaptotagmin-1, -2, and -9: Ca$^{(2+)}$ sensors for fast release that specify distinct presynaptic properties in subsets of neurons. Neuron *54*, 567–581.

Cell Reports, Volume *15*

Supplemental Information

PRRT2 Is a Key Component of the Ca^{2+}-Dependent Neurotransmitter Release Machinery

Pierluigi Valente, Enrico Castroflorio, Pia Rossi, Manuela Fadda, Bruno Sterlini, Romina Ines Cervigni, Cosimo Prestigio, Silvia Giovedì, Franco Onofri, Elisa Mura, Fabrizia C. Guarnieri, Antonella Marte, Marta Orlando, Federico Zara, Anna Fassio, Flavia Valtorta, Pietro Baldelli, Anna Corradi, and Fabio Benfenati

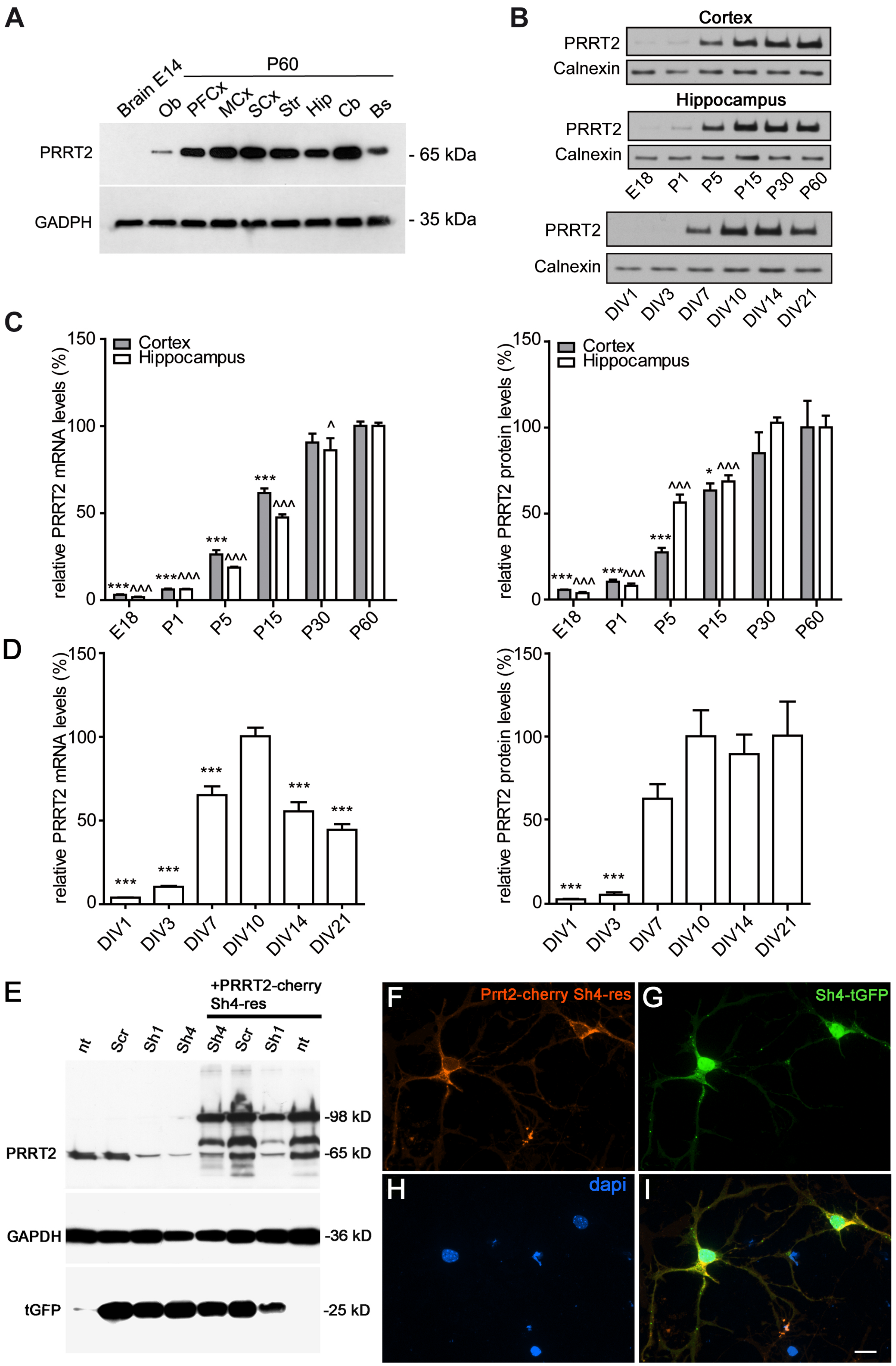
A
Brain E14
Ob
PFCx
MCx
SCx
Str
Hip
Cb
Bs
P60
PRRT2
- 65 kDa
GADPH
- 35 kDa
B
Cortex
PRRT2
Calnexin
Hippocampus
PRRT2
Calnexin
E18
P1
P5
P15
P30
P60
PRRT2
Calnexin
DIV1
DIV3
DIV7
DIV10
DIV14
DIV21
C
Cortex
Hippocampus
relative PRRT2 mRNA levels (%)
relative PRRT2 protein levels (%)
D
relative PRRT2 mRNA levels (%)
relative PRRT2 protein levels (%)
E
+PRRT2-cherry
Sh4-res
nt
Scr
Sh1
Sh4
Sh4
Scr
Sh1
nt
PRRT2
-98 kD
-65 kD
GAPDH
-36 kD
tGFP
-25 kD
F
Prrt2-cherry Sh4-res
G
Sh4-tGFP
H
dapi
I

Figure S1

Figure S1 (related to Fig. 1). Physiological expression of PRRT2 and downregulation in primary neurons by RNA interference

A. Regional expression of PRRT2 in the mouse brain. Western blot analysis of PRRT2 protein levels in mouse brain regions at embryonic (E14) and postnatal (P60) stages of development. Brain, total brain; Ob, olfactory bulb; PFCx, Prefrontal cortex; MCx, Motor cortex; SCx, Somatosensory cortex; Str, striatum, Hip, hippocampus; Cb, cerebellum, Bs, brain stem. GAPDH was used as loading control.

B-D. Temporal expression profile of PRRT2 in the developing mouse brain and in primary hippocampal neurons. **B**. Representative immunoblots of PRRT2 and calnexin expression in the cortex and hippocampus of developing mice and in primary hippocampal neurons as a function of the days in vitro (DIV). **C**. Expression profile of PRRT2 mRNA (*left*) and protein (*right*) analyzed by qRT-PCR and western blotting, respectively in the cerebral cortex (grey bars) and hippocampus (open bars) of developing mice (from embryonic day 18 to postnatal day 60). The levels of PRRT2 mRNA and protein were very low, albeit detectable, during the first stages of development (E18 and P1), increased between P5 and P30 and remained almost stable in the adulthood. Data are expressed as mean percentage intensity (± SEM) of 3 different animals with respect to P60. *$p<0.05$, ***$p<0.001$, *vs* P60 cortex; ^$p<0.05$, ^^^$p<0.001$ *vs* P60 hippocampus; one-way ANOVA followed by the Dunnett's multiple comparison test. **D**. Expression profile of PRRT2 mRNA (*left*) and protein (*right*) analyzed in primary cultures of E18 hippocampal neurons at various stages of development (from 1 to 21 DIV). PRRT2 mRNA and protein levels were barely detectable during the first stages of development (1-3 DIV), started to increase at DIV 7, peaked at DIV 10 and remained relatively high and stable thereafter. Data are expressed as mean percentage intensity ± SEM of 3 independent cultures with respect to DIV10. ***$p<0.001$ *vs* DIV10; one-way ANOVA/Dunnett's multiple comparison test.

E-I. Silencing and rescue of endogenous PRRT2 in primary neurons. **E.** Primary neurons were transduced at 6 DIV with lentiviral vectors (pLKO.1-CMV-turboGFP) coding for Sh1, Sh4 or Scr with or without co-infection with the appropriate Sh-resistant PRRT2 construct fused to mCherry, and analyzed by western blotting at 12 DIV. Endogenous PRRT2 (65 KDa apparent molecular mass) was silenced by either Sh1 or Sh4, whereas its expression was not affected by the Scr shRNA. When neurons were co-transduced with lentiviruses expressing Sh4 and the Sh4-resistant PRRT2, the endogenous PRRT2 (65 KDa) was still downregulated, while the exogenous sh-resistant PRRT2 fused to mCherry (98 KDa) was fully expressed. nt, untreated neurons. GAPDH was used as loading control, tGFP was used as ShRNA transfection reporter. **F-I.** The expression of the Sh4-resistant PRRT2, visualized by the intrinsic mCherry fluorescence (**F**), was not affected by the expression of Sh4, visualized by the green fluorescence of tGFP in the same cells (**G**). **H.** DAPI staining. **I.** Merged image of panels F, G and H. Scale bar, 20 µm.

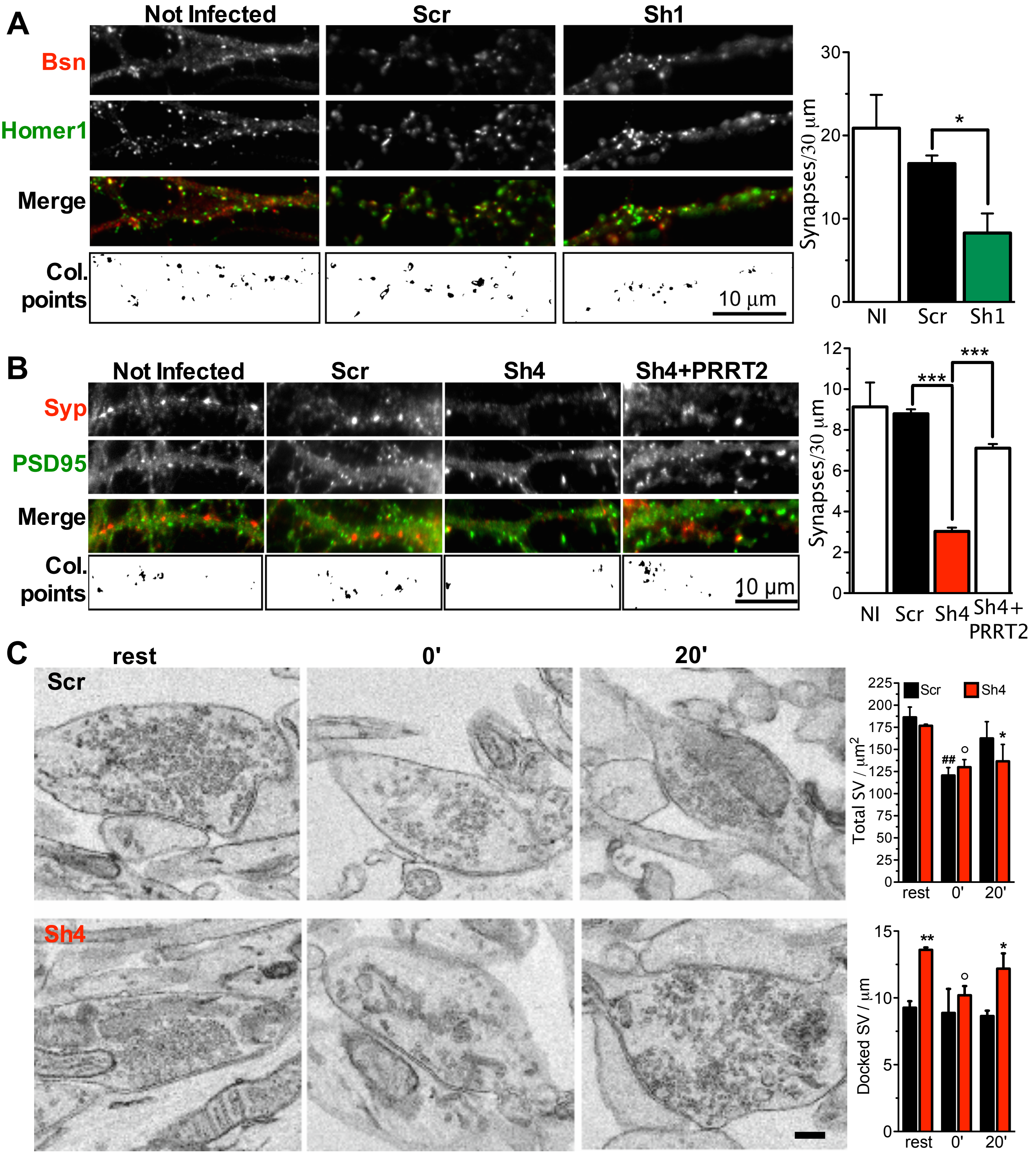
A
Not Infected
Scr
Sh1
Bsn
Homer1
Merge
Col. points
10 µm
Synapses/30 µm
30
20
10
0
*
NI
Scr
Sh1
B
Not Infected
Scr
Sh4
Sh4+PRRT2
Syp
PSD95
Merge
Col. points
10 µm
Synapses/30 µm
12
10
8
6
4
2
0
***
***
NI
Scr
Sh4
Sh4+ PRRT2
C
rest
0'
20'
Scr
Sh4
Scr
Sh4
Total SV / µm²
225
200
175
150
125
100
75
50
25
0
##
o
*
rest
0'
20'
Docked SV / µm
15
10
5
0
**
o
*
rest
0'
20'

Figure S2

Figure S2 (related to Fig. 2). PRRT2 knockdown decreases the total number of excitatory synapses and alters synaptic ultrastructure in an activity-dependent fashion

A. *Left:* Representative images of dendrites of hippocampal neurons infected at 7 DIV with Scr, Sh1 or left uninfected (NI) and analyzed at 14 DIV. Synaptic boutons were identified by double immunostaining for Bassoon (Bsn; red) and Homer1 (green). The colocalization panels (Col. points) highlight the double-positive puncta (black) corresponding to *bona fide* synapses. Scale bar, 10 μm. *Right:* Quantitative analysis of synaptic puncta counted on 30-μm dendrite tracts starting from the cell body. Data are means ± SEM from n=3 independent experiments, each carried out in duplicate. Five dendrites for each neuron, from at least 10 neurons for each sample were counted. *p<0.05, one-way ANOVA/Bonferroni's multiple comparison test.

B. *Left:* Representative images of dendrites of hippocampal neurons infected at 7 DIV with Scr, Sh4, Sh4 + Sh4-resistant PRRT2 or left uninfected (NI) and analyzed at 14 DIV. Synaptic boutons were identified by double immunostaining for a distinct couple of pre/post-synaptic markers, namely synaptophysin (Syp; red) and PSD95 (green). The colocalization panels (Col. points) highlight the double-positive puncta (black) corresponding to *bona fide* synapses. Scale bar, 10 μm. *Right:* Quantitative analysis of synaptic puncta counted on 30-μm dendrite tracts starting from the cell body. Data are means ± SEM from n=3 independent experiments, each carried out in duplicate. Five dendrites for each neuron, from at least 10 neurons for each sample were counted. ***p<0.001, one-way ANOVA/Bonferroni's multiple comparison test.

C. *Left:* Representative transmission electron micrographs of presynaptic terminals from low-density hippocampal neurons infected at 7 DIV with either Scr (upper row) or Sh4 (lower row). Synaptic ultrastructure was evaluated by fixing neurons under basal conditions (left column), immediately after a train of 300 APs @ 10 Hz (0'; middle column) and after 20 min of recovery (20'; right column). Scale bar, 200 nm. *Right:* Results of the ultrastructural analysis. Bar plots show the mean (± SEM) total SV density (top) and docked SV density (bottom) calculated in Scr-treated (black bars) and Sh4-treated (red bars) neurons subjected to the depression/recovery protocol. No significant changes in the nerve terminal area and in the length of the AZ were found among the experimental groups. Synaptic area (mean ± SEM, μm^2): rest, 0.59±0.03 and 0.61±0.06; 0', 0.67±0.06 and 0.56±0.02; 20', 0.64±0.07 and 0.79±0.08; for Src- and Sh4-treated neurons, respectively. AZ length (mean ± SEM, μm): rest, 0.29±0.03 and 0.30±0.03; 0', 0.34±0.04 and 0.31±0.02; 20', 0.33±0.05 and 0.33±0.02; for Src- and Sh4-treated neurons, respectively. *p<0.05, **p<0.01, across genotype; ^{o}p<0.05, ^{oo}p<0.01, within genotype; one-way ANOVA for repeated measures/Bonferroni's multiple comparison test. n = 90 images per genotype from 3 independent experiments.

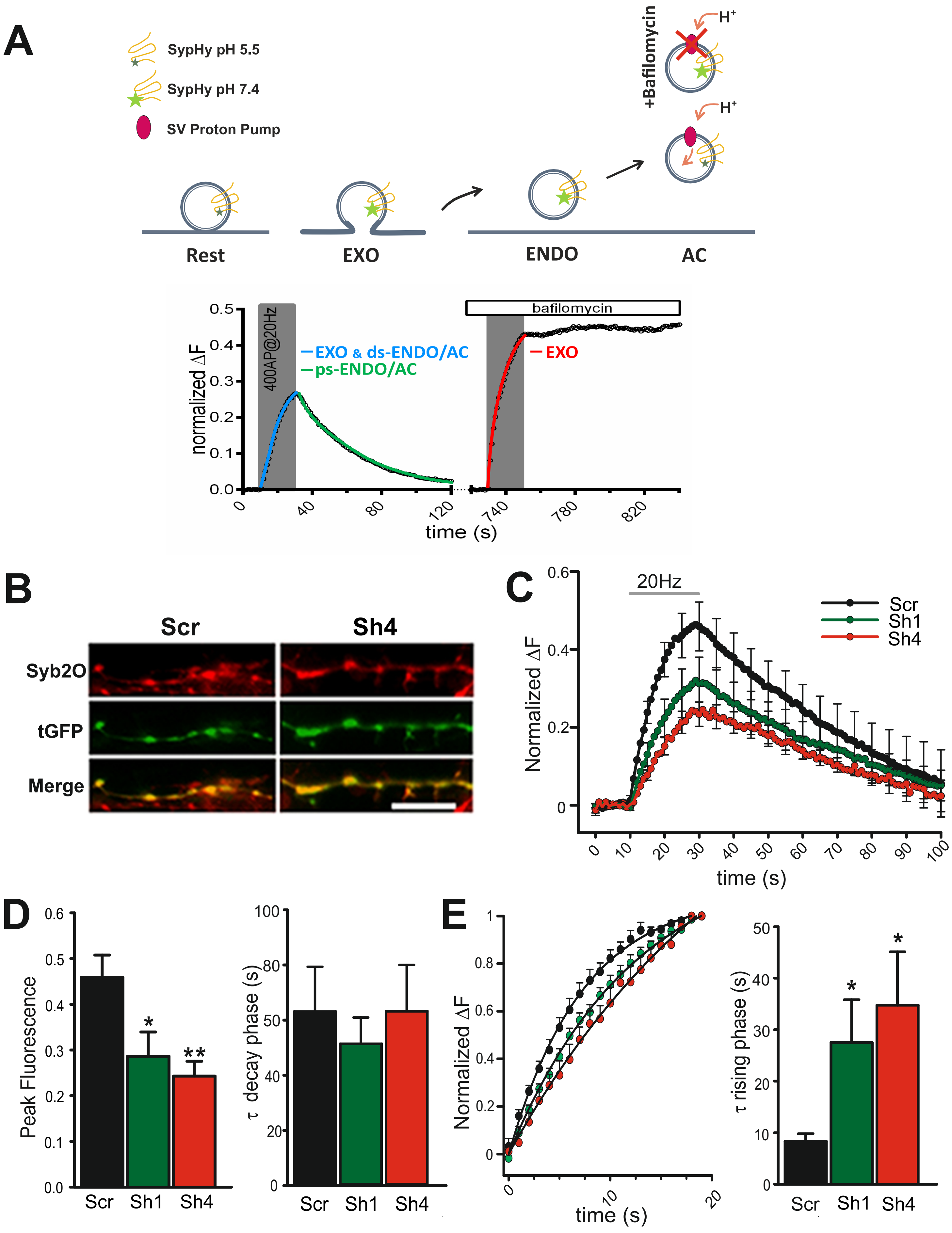
A
SypHy pH 5.5
SypHy pH 7.4
SV Proton Pump
+Bafilomycin
H+
Rest
EXO
ENDO
AC
bafilomycin
400AP@20Hz
EXO & ds-ENDO/AC
ps-ENDO/AC
EXO
normalized ΔF
time (s)
B
Scr
Sh4
Syb2O
tGFP
Merge
C
20Hz
Scr
Sh1
Sh4
Normalized ΔF
time (s)
D
Peak Fluorescence
τ decay phase (s)
Scr Sh1 Sh4
E
Normalized ΔF
time (s)
τ rising phase (s)

Figure S3

Figure S3 (related to Fig. 3). PRRT2 knockdown by either Sh1 or Sh4 slows down the kinetics of release in low-density hippocampal neurons as evaluated by the Syb2O reporter of exo-endocytosis

A. Schematic diagram illustrating the pH-sensitive features of synaptophysin-pHluorin (SypHy) to assay SV cycling. *Upper panel*: SypHy fluorescence is quenched by the lumenal acidic pH (5.5) of SVs at rest. Upon exocytosis (EXO) fluorescence increases due the exposure of lumenal EGFP to the extracellular pH (7.4). Endocytosis (ENDO) and the subsequent acidification (AC) of SVs quench the SypHy fluorescence again, a process that can be blocked by H^+-ATPase inhibitors such as bafilomycin. *Lower panel*: Representative time-course of SypHy fluorescence. EXO is induced by repetitive field stimulation (400APs@20Hz) in the absence (*left*) or presence (*right*) of bafilomycin. The blue line represents the rate of SypHy fluorescence increase during stimulation and is contributed by EXO + during-stimulus (DS) ENDO/AC. The green line indicates the rate of SypHy fluorescence decay after stimulation and reflects post-stimulus (PS) ENDO/AC. The rate of SypHy fluorescence increase during stimulation in presence of bafilomycin (red line) represents pure EXO.

B. Representative experimental field showing hippocampal neurites co-expressing Syb2O and PRRT2 Sh4 or Scr (tGFP). The merge panel shows the virtually complete colocalization of the two markers at synaptic puncta. Scale bar, 10 µm.

C. Ensemble averaged Syb2O traces from PRRT2-KD synapses (Sh1, green trace; Sh4, red trace) and control synapses (Scr, black trace) recorded in response to electrical field stimulations at 20 Hz for 20s (gray horizontal line) and normalized to the maximum fluorescence intensity reached under NH_4Cl perfusion (Normalized ΔF). Data are means ± SEM values shown every five time points.

D. Quantitative evaluation of the SV pool released during the stimulation (left panel) and plotted as peak fluorescence reached at the end of the stimulation for Sh1- (green), Sh4- (red) and Scr- (black) treated synapses and of the kinetics of post-stimulus endocytosis (τ decay phase; right panel) determined by exponential fitting of individual experiments for neurons treated with Scr (black bars), Sh1 (green bars) or Sh4 (red bars) shRNA.

E. Kinetics of the fluorescence rising phase during stimulation in the experiments shown in (D) and relative rates (τ) determined in the exponential fitting procedure.

With both shRNAs, the data precisely reproduce the changes induced by PRRT2 silencing that were evaluated by Syphy in Fig. 3. Data are expressed as mean ± SEM from 7 (Sh1, 120 synapses), 7 (Sh4, 140 synapses) and 6 (control, 130 synapses) experiments from 3 different preparations. $^*p<0.05$, $^{**}p<0.01$ *vs* Scr, ANOVA on Ranks/Dunn's multiple comparison test.

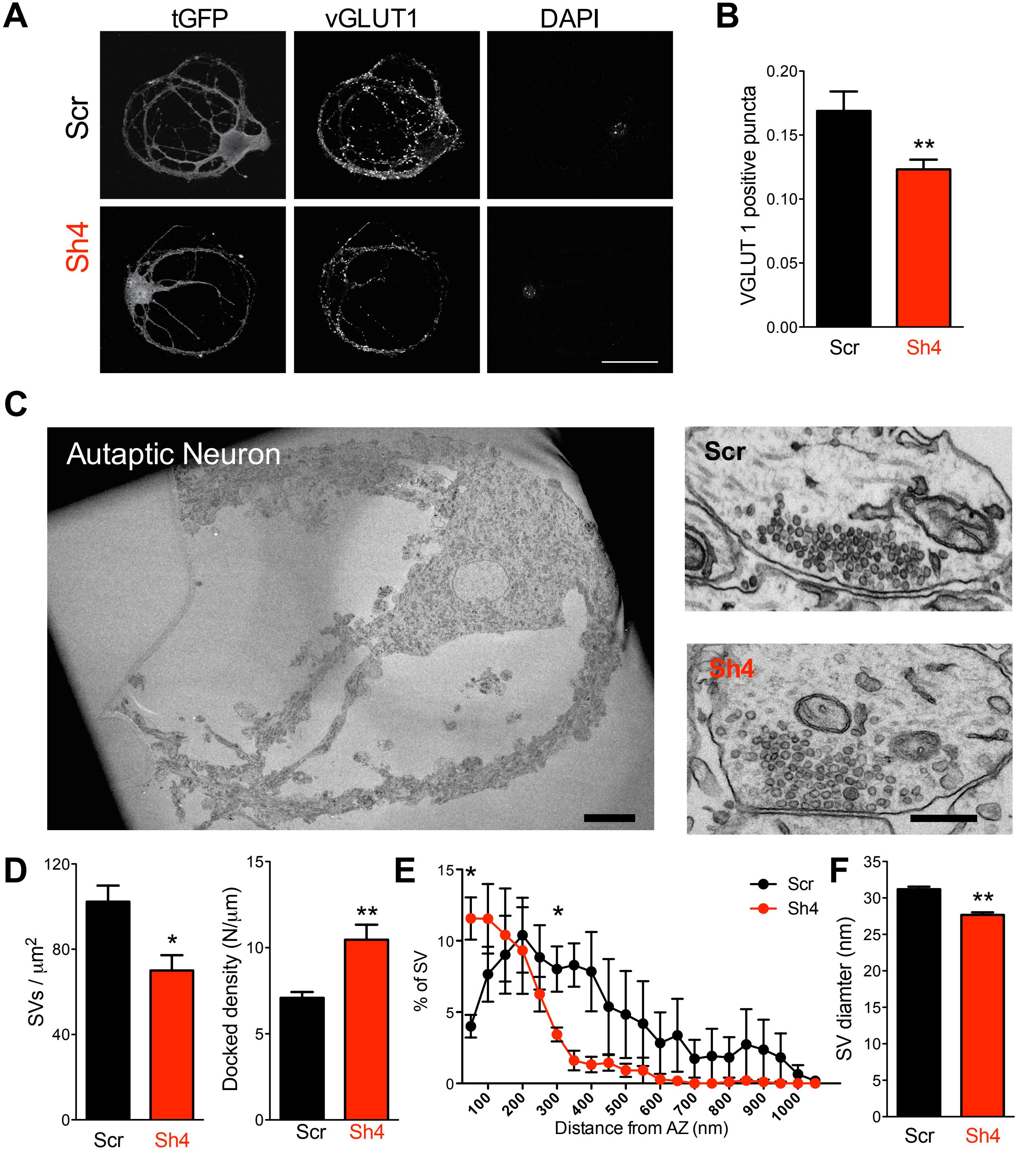
A
tGFP
vGLUT1
DAPI
Scr
Sh4
B
VGLUT 1 positive puncta
0.20
0.15
0.10
0.05
0.00
**
Scr
Sh4
C
Autaptic Neuron
Scr
Sh4
D
SVs / μm2
120
80
40
0
*
Scr
Sh4
Docked density (N/μm)
15
10
5
0
**
Scr
Sh4
E
% of SV
15
10
5
0
*
*
Scr
Sh4
100
200
300
400
500
600
700
800
900
1000
Distance from AZ (nm)
F
SV diamter (nm)
35
30
25
20
15
10
5
0
**
Scr
Sh4

Figure S4

Figure S4 (related to Fig. 4). Silencing of PRRT2 in autaptic hippocampal neurons decreases the number of synaptic contacts and alters SV pools

A. Representative images of autaptic neurons transduced with either Scr or PRRT2 shRNA (Sh4; tGFP fluorescence) and stained with an anti-vGLUT1 antibody. Scale bar, 20 µm.

B. Quantitative evaluation of the density of vGLUT1-positive puncta. Values are expressed as means ± SEM (n = 30 for both control and PRRT2-KD neurons).

C. Ultrastructural analysis of autaptic synaptic contacts. Representative EM images of control and silenced autaptic neurons: from left to right: low magnification electron micrograph of an entire autaptic neuron (scale bar, 20 µm) and close-ups of control and PRRT2-KD autaptic neurons at 14 DIV (scale bar, 200 nm). The synaptic area and active zone length were comparable in control and silenced autaptic neurons (synaptic area: 1.039 ± 0.099 μm^2 and 0.967 ± 0.062 μm^2; active zone length: 0.455 ± 0.027 µm and 0.403 ± 0.035 µm; means ± SEM from 86 and 65 autapses for Scr and PRRT2 sh-RNA-infected neurons, respectively from 5 independent neuronal preparations).

D. Morphometric analysis of control and PRRT2-KD synapses. PRRT2 KD synapses show a decreased number of SVs with a higher number of SV docked at the AZ.

E. Distribution of SVs in autaptic terminals of Scr-treated (black symbols) and Sh4-treated (red symbols) neurons. The percentage of SVs located within successive 50 nm shells from the AZ is given as mean ± SEM as a function of the distance from the AZ.

F. Morphometric analysis of SV diameter in control and PRRT2-KD synapses.

Data are shown as means ± SEM from n= 86 and 65 for control and PRRT2-KD neurons, respectively from n=5 independent preparations. *p<0.05, **p<0.01, unpaired Student's *t*-test (D,F) and Kolgomorov-Smirnov test (E).

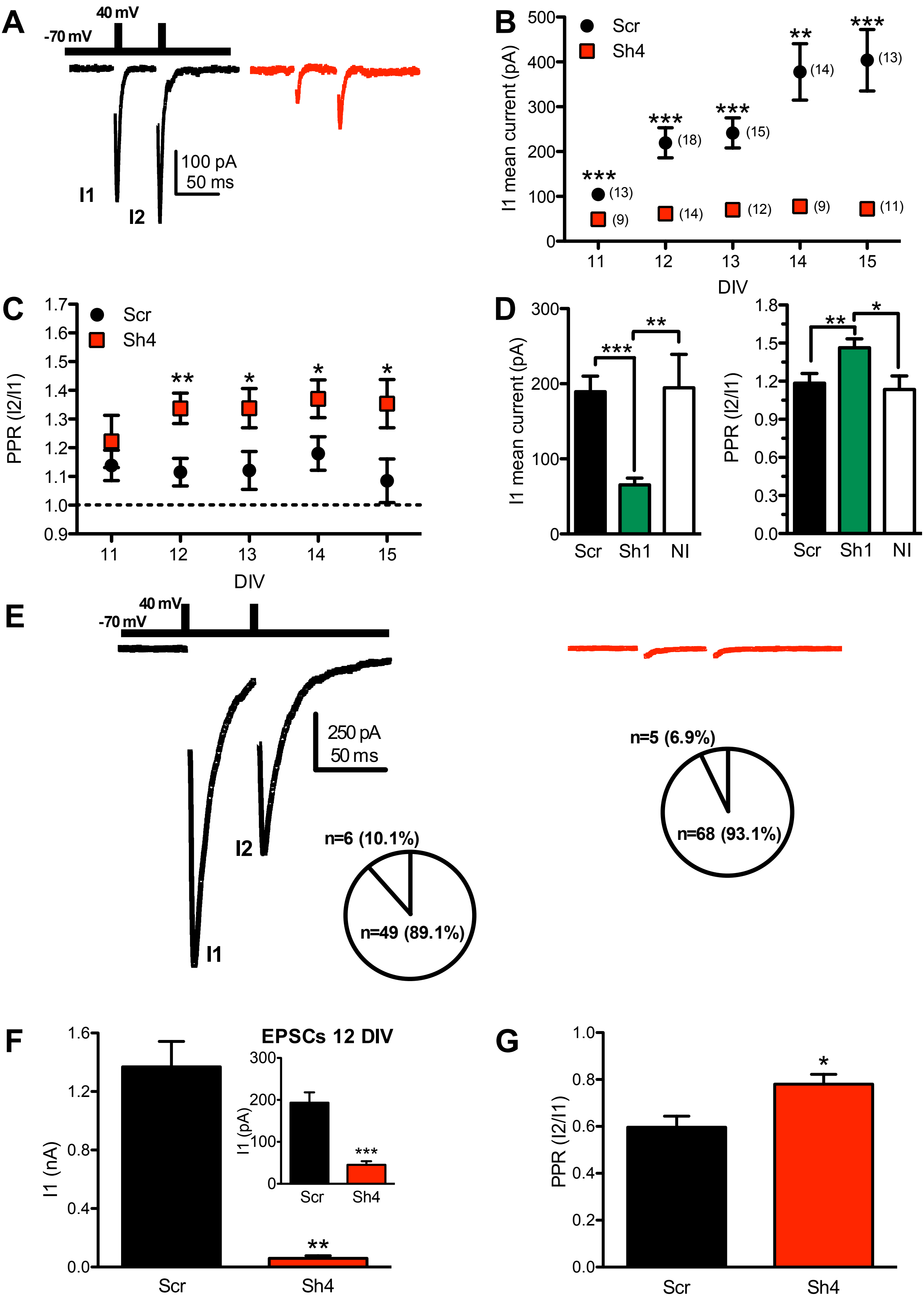
A
40 mV
-70 mV
100 pA
50 ms
I1
I2
B
Scr
Sh4
I1 mean current (pA)
DIV
C
Scr
Sh4
PPR (I2/I1)
DIV
D
I1 mean current (pA)
PPR (I2/I1)
Scr
Sh1
NI
E
40 mV
-70 mV
250 pA
50 ms
I1
I2
n=6 (10.1%)
n=49 (89.1%)
n=5 (6.9%)
n=68 (93.1%)
F
EPSCs 12 DIV
I1 (nA)
I1 (pA)
Scr
Sh4
G
PPR (I2/I1)
Scr
Sh4

Figure S5

Figure S5 (related to Fig. 4) Knockdown of PRRT2 impairs PSCs in both glutamatergic and GABAergic hippocampal autaptic neurons

A. Representative recording traces of eEPSCs from PRRT2-KD synapses (Sh4, red traces) and control synapses (Scr, black trace).

B,C. eEPSC amplitude evoked by the first pulse (I1; **B**) and paired pulse ratio (PPR=I2/I1; **C**) in neurons transduced with Scr (black symbols) or Sh4 (red symbols) as a function of the days in vitro (DIV). The number of cells recorded in n=3 independent cell culture preparations ranged between 9 and 18.

D. Average eEPSCs amplitude (I1; left) and PPR (right) evoked in autaptic cells infected with lentiviral vectors coding for either Scr (n=24; black bars) or PRRT2-Sh1 (n= 20; green bars). Data obtained in non-infected cells (n= 10; open bars) are shown for comparison.

E. Representative eIPSCs recorded in autaptic neurons infected with either Scr (black trace) or Sh4 (red trace). eIPSCs were evoked by two voltage steps to +40 mV, lasting 0.5 ms, applied at an interpulse interval of 50 ms from a holding potential of -70 mV. Inset: pie plots showing the percent of inhibitory neurons responding to electrical stimulation with respect to the total neurons transduced with either Scr (left) or Sh4 (right). In all synaptic currents, the Na^+ current was blanked for clarity.

F,G. Average eIPSC amplitude (**F**) and PPR (**G**) in neurons transduced with either Scr (black bar; n=6) or Sh4 (red bar; n=5) and recorded at 11-15 DIV. The inset in B shows the change of EPSCs evoked in glutamatergic autaptic neurons in the very same culture preparations and recorded at 12 DIV (Scr: black bars, n=9; Sh4: red bars, n=6).

All data are expressed as means ± SEM. *p<0.05; **p<0.01, ***p<0.001; panels B,C,F,G: unpaired Student’s *t*-test or Mann-Whitney U test; panel D: one-way ANOVA/Bonferroni's multiple comparison test (I1) and Kruskal-Wallis/Dunn's multiple comparison test (PPR).

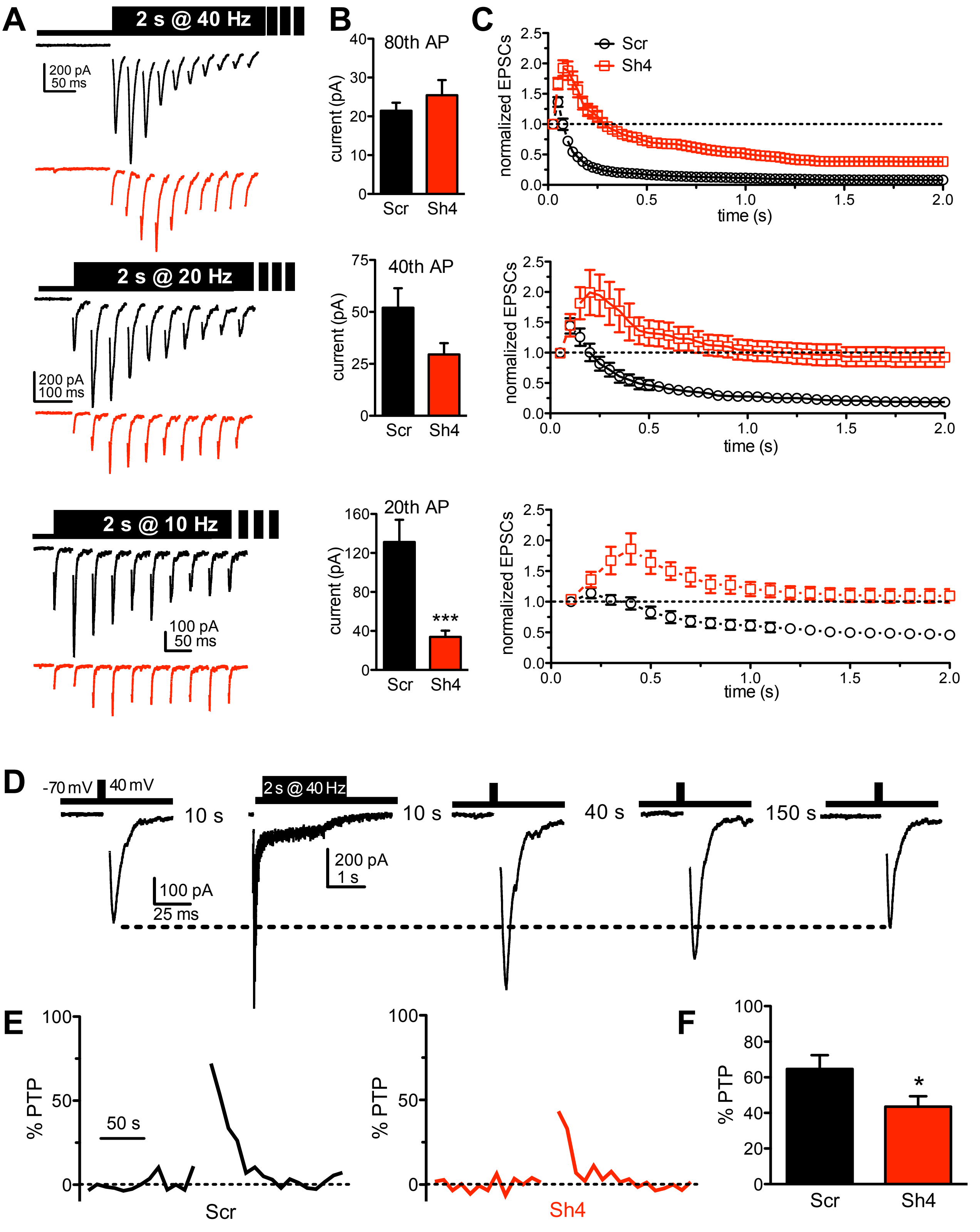
A
2 s @ 40 Hz
200 pA
50 ms
B
80th AP
current (pA)
Scr
Sh4
C
normalized EPSCs
Scr
Sh4
time (s)
2 s @ 20 Hz
200 pA
100 ms
40th AP
2 s @ 10 Hz
100 pA
50 ms
20th AP
***
D
-70 mV
40 mV
10 s
2 s @ 40 Hz
10 s
40 s
150 s
200 pA
1 s
100 pA
25 ms
E
% PTP
50 s
Scr
Sh4
F
*

Figure S6

Figure S6 (related to Fig. 6). PRRT2 knockdown dramatically enhances synaptic facilitation and reduced post-tetanic potentiation at excitatory autapses

A. PRRT2 knockdown enhances synaptic facilitation and attenuates synaptic depression. Representative traces showing synchronous EPSCs evoked by tetanic stimulation of 2 s @ 40 (top), 20 (middle) and 10 (bottom) Hz in autaptic neurons transduced with either Scr (black) or PRRT2-Sh4 (red) (40 Hz: Scr n=38, Sh4 n=34; 20 Hz: Scr n=14, Sh4 n=11; 10 Hz: Scr n=12, Sh4 n=11). Stimulation artifacts were blanked for clarity. Only the first 10 currents in the train are shown.

B. Mean ± SEM of the absolute values of the last eEPSC in the train in autaptic neurons transduced with either Scr (black bars) or Sh4 (red bars) are shown.

C. Mean (± SEM) normalized values of eEPSCs amplitude showing the time course of synaptic facilitation and depression in autaptic neurons treated as in A.

D. PRRT2 knockdown decreases post-tetanic potentiation**.** Representative eEPSCs evoked before and 10, 40 and 150 s after tetanic stimulation (2 s@40 Hz). Stimulation artifacts were blanked for clarity.

E. Representative time-course of the normalized eEPSC amplitude in an autaptic neuron infected with either Scr (black trace) or Sh4 (red trace).

F. Mean (± SEM) percentages of post-tetanic potentiation in autaptic neurons transduced with Scr (n=30) or Sh4 (n=28). All data are expressed as means ± SEM. *p<0.05, ***p<0.001, unpaired Student's *t*-test or Mann-Whitney's U-test.

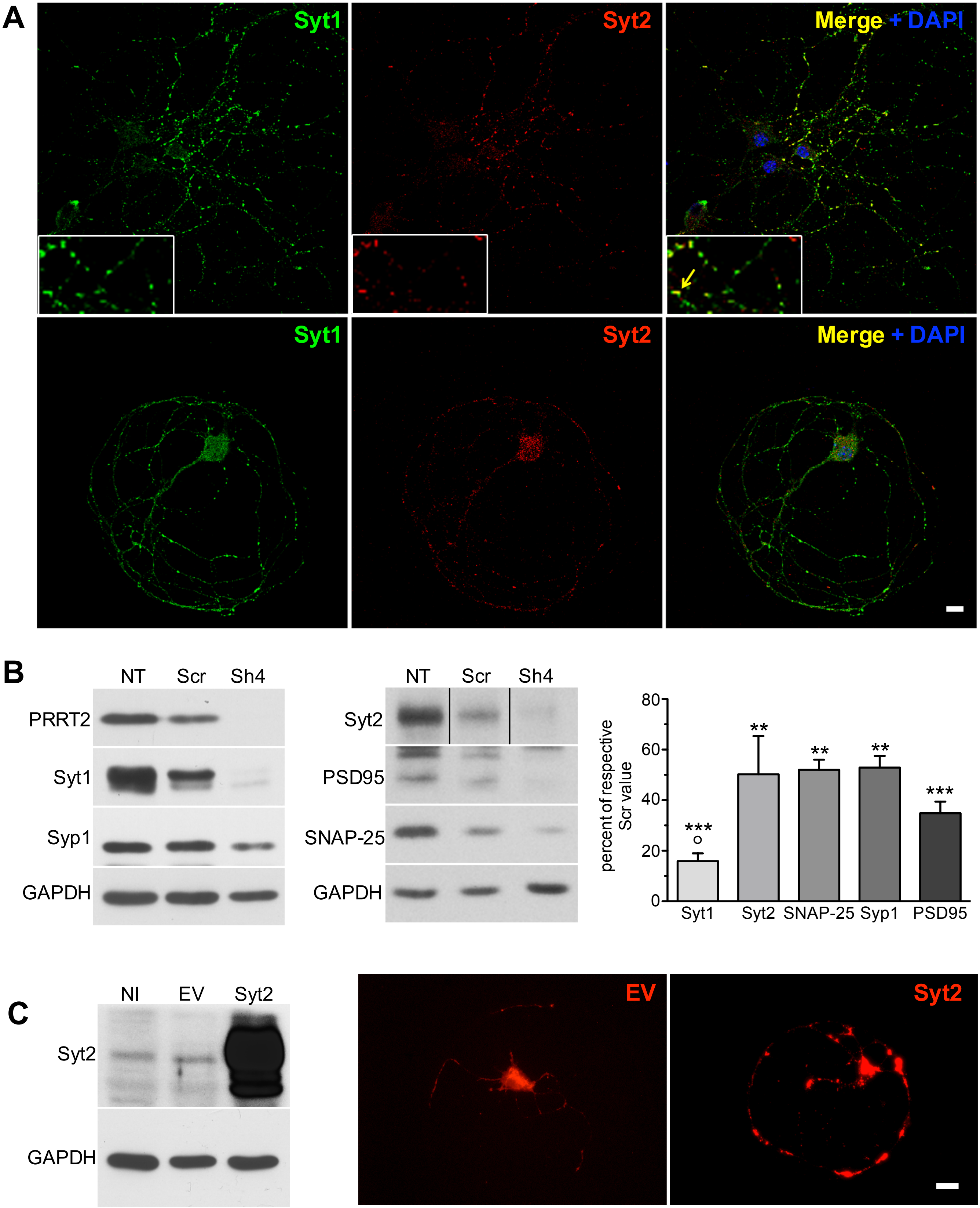
A
Syt1
Syt2
Merge + DAPI
Syt1
Syt2
Merge + DAPI
B
NT
Scr
Sh4
PRRT2
Syt1
Syp1
GAPDH
NT
Scr
Sh4
Syt2
PSD95
SNAP-25
GAPDH
percent of respective Scr value
80
60
40
20
0
***
**
**
**
***
Syt1
Syt2
SNAP-25
Syp1
PSD95
C
NI
EV
Syt2
Syt2
GAPDH
EV
Syt2

Figure S7

Figure S7 (related to Fig. 7). Expression of Syt1 and Syt2 in low-density and autaptic primary hippocampal neurons after PRRT2 silencing

A. Double-staining of low-density hippocampal neurons (*top row*) or autaptic hippocampal neurons (*bottom row*) with rabbit Syt1 antibodies (1:250; green) and mouse Syt2 antibodies (1:25; red). In the merge panel shown on the right, nuclei are stained with DAPI (blue). Insets: higher magnification of synaptic boutons shows partial co-localization of the two Syt isoforms (arrow). Scale bar, 10 μm.

B. Expression levels of pre- and post-synaptic proteins in low-density hippocampal neurons transduced with either Scr or Sh4 or left non-infected. Neurons were infected at 7 DIV and harvested at 15 DIV. Samples were separated by SDS-PAGE and analyzed by immunoblotting with antibodies to Syt1, Syt2, SNAP-25, synaptophysin-1 (Syp1) and PSD95. *Left:* a representative immunoblot is shown. Vertical lines in the blot indicate that the lanes were on the same gel, but have been repositioned in the figure. Immunoreactivity for GAPDH was used as a control for equal loading. *Right:* Immunoblots were quantified by densitometric analysis. The expression level of the various presynaptic proteins in PRRT2-KD neurons, calculated in percent of the levels in Scr-treated neurons, is shown as means ± SEM of n=5 independent experiments. **$p<0.01$, ***$p<0.001$ *vs* Scr-treated neurons; °$p<0.05$ Syt1 *vs* other synaptic proteins; ANOVA/Dunnett's multiple comparison test.

C. Overexpression of Syt2 in low-density and autaptic primary neurons. Left: representative western blot of Syt2 expression in low-density hippocampal neurons that were non-infected (NI), infected with the empty vector (EV) or infected with the Syt2 overexpressing vector. Immunoreactivity for GAPDH was used as a control for equal loading. Right: Syt2 immunoreactivity in autaptic neurons that were transduced with either empty vector (EV) or Syt2 overexpressing vector. Scale bar, 10 μm.

SUPPLEMENTAL EXPERIMENTAL PROCEDURES

Generation of PRRT2 shRNAs and sh-resistant PRRT2. Based on the ORF of mouse PRRT2 transcript, 5 shRNAs were designed (Mission shRNA custom cloning, Sigma-Aldrich):
Sh1: GGCCACAGACCTCAGTTTAAA
Sh2: GAGGTCCTGACAGAAAGTACA
Sh3: CTCGGGACTATATCATCCTTG
Sh4: CATGTGGCCTGTCAACATTGT
Sh5: TGTCCAGGTTGTAGCAGAGAT
As a negative control, we used the following scrambled ShRNA sequence, based on the sequence of Sh1: GCCGACACTCGTCAGTTTAAA. An additional control ShRNA, with a fully unrelated sequence that does not target any known human or mouse genes (Luciferase shRNA; Sigma-Aldrich) was occasionally used with similar results. Sequence alignment of human and mouse PRRT2 regions targeted by the five shRNAs (see below, highlighted in yellow) revealed the presence of several mismatches with the human sequence, accounting for the specificity of the shRNAs in knocking down the endogenous mouse PRRT2 expression.

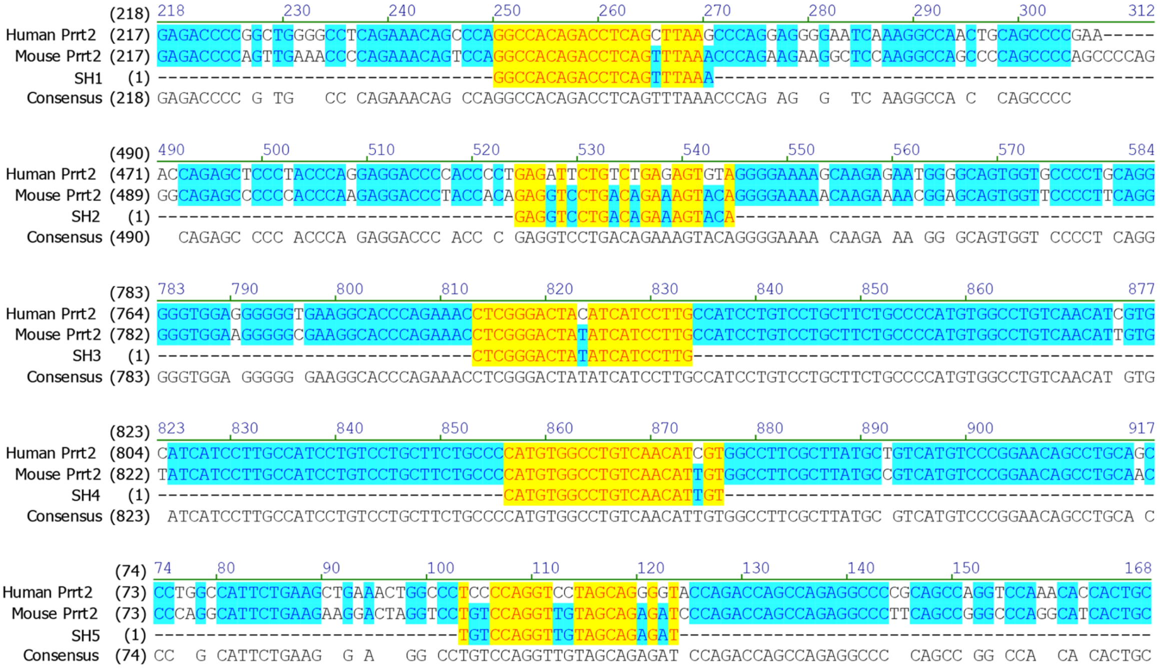

For rescue experiments, we generated PRRT2-Cherry Sh1-res and PRRT2-Cherry Sh4-res constructs carrying silent mutations that render them insensible to knocking-down.
SH1 resistant: GGCCACAGACCTGAGCCTGAA
SH4 resistant: CATGTGGCCAGTGAATATCGT

Synaptosome preparation. Synaptosomes were purified from the P2 fractions by centrifugation on discontinuous Percoll gradient from the pooled cortex of three adult mice (3-6 months old). The tissue was homogenized in 10 ml of ice-cold HB buffer (0.32 M sucrose, 1 mM EDTA, 10 mM Tris, pH 7.4) containing protease inhibitors, using a glass-Teflon homogenizer. The resultant homogenate was centrifuged at 1,000 x g for 5 min at 4°C in order to remove nuclei and debris and the supernatant was further centrifuged at 18,900 x g for 10 min at 4°C to obtain the P2 fraction. The pellet was resuspended in 2 ml of HB buffer and gently stratified on a discontinuous Percoll gradient (3%-10%-23%). After a 10 min centrifugation at 18,900 x g at 4°C, the synaptosomal fraction from the 10% and 23% Percoll interface was collected, washed in Krebs buffer (140 mM NaCl, 5 mM KCl, 5 mM $NaHCO_3$, 1.3 mM $MgSO_4$, 1 mM phosphate buffer pH 7.4, 10 mM Tris/Hepes pH 7.4) to eliminate Percoll and used as the starting material for subsequent ultrafractionation.

Ultrasynaptic fractionation. Ultrasynaptic fractionation was performed as previously described (Phillips et al., 2001; Feligioni et al., 2006). Briefly, synaptosomes were pelleted by centrifugation (16,000 x g, 5 min, 4°C) and resuspended in 300 µl of 0.32 M sucrose, 0.1 mM $CaCl_2$. An aliquot was removed and kept as total. Protease inhibitors were used in all purification steps. Synaptosomes were then diluted 1:10 in ice-cold 0.1 mM $CaCl_2$ and mixed with an equal volume of 2X solubilization buffer (2 % Triton X-100, 40 mM Tris, pH 6, 4°C). After

a 30 min incubation at 4°C, the insoluble material (synaptic junction) was pelleted by centrifugation (40,000 x g, 30 min, 4°C). The supernatant (non-synaptic synaptosomal protein; NSSP) was decanted and the proteins precipitated with 6 volumes of acetone at -20°C overnight and then centrifuged (18,000 x g, 30 min, -15°C). The synaptic junction pellet (containing the insoluble postsynaptic density and the presynaptic active zone) was resuspended in 10 volumes of 1X solubilization buffer (1% Triton X-100, 20 mM Tris, pH 8, 4°C), incubated for 30 min at 4°C and then centrifuged (40,000 x g, 30 min, 4°C). The proteins in the supernatant (active zone, AZ) were precipitated in 6 volumes of acetone at -20°C overnight and then centrifuged (18,000 x g, 30 min, -15°C), while the pellet was kept as postsynaptic density fraction (PSD). All pellets (PSD, AZ, NSSP) were resuspended in 5% SDS, quantified by BCA assay, and loaded on SDS-PAGE gels for electrophoresis and consecutive Western Blotting.

Subcellular fractionation for SV purification. Subcellular fractions were prepared from rat forebrain, and SVs were purified through the step of controlled-pore glass chromatography as described (Huttner et al., 1983). Aliquots of purified SVs were exposed to 200 mM NaCl treatment, which results in quantitative removal of extrinsic SV proteins such as synapsin I. Both untreated and salt-treated SVs (USV and SSV, respectively) were resuspended in 0.3 M glycine, 5 mM Hepes/NaOH, pH 7.4, at a protein concentration of 1.5–2 mg/ml.

Western blotting and antibodies. Protein concentration of the samples was determined using the BCA or Bradford assay and equivalent amounts of protein were subjected to SDS-PAGE on 10-12% polyacrylamide gels and blotted onto nitrocellulose membranes (Whatman). Blotted membranes were blocked for 1 h in 5% milk in Tris-buffered saline (10 mM Tris, 150 mM NaCl, pH 8.0) plus 0.1% Triton X-100 and incubated overnight at 4°C with the following primary antibodies: rabbit anti-PRRT2 (1:500, Sigma-Aldrich), rabbit anti-synaptophysin-1 (1:5000, Synaptic Systems), rabbit anti-SNAP-25 (1:1000, Cell Signaling), mouse anti-PSD95 (1:3000, Synaptic Systems), rabbit anti-Synaptotagmin-1 (1:1000, Synaptic Systems), rabbit anti-Synaptotagmin-2 (1:1000, Synaptic Systems), rabbit anti-Syntaxin 1A (1:1000, Synaptic Systems), rabbit anti-Vamp2 (1:1000, Synaptic Systems), rabbit anti-Munc18-1 (1:2000, Synaptic Systems), rabbit anti-Complexin 1/2 (1:2000, Synaptic Systems), rabbit anti-RIM1 (1:1000, Synaptic Systems), rabbit anti-RIM binding protein-1 (1:1000, Synaptic Systems), rabbit anti-GAPDH (1:10000, Cell Signaling), rabbit anti-calnexin (1:15000, Sigma-Aldrich). Membranes were washed and incubated for 1 h at room temperature with peroxidase-conjugated goat anti-rabbit (1:5000; Bio-Rad) or with peroxidase-conjugated goat anti-mouse (1:5000; Bio-Rad) antibodies. Bands were revealed with the ECL chemiluminescence detection system (Thermo Scientific, Waltham, Ma, USA).

Pull down and co-immunoprecipitation assays. Nterm-3XFlag-PRRT2 and Flag-bacterial alkaline phosphatase (3XFlag-BAP) as a control (Sigma-Aldrich, cat. E7658) were overexpressed in HEK293T cells and purified by anti-Flag M2 Affinity gel (Sigma-Aldrich). The immunocomplex was incubated for 4 hrs with crude synaptosome extracts (P2) prepared from mouse brain in lysis buffer (1% Triton X-100, 150 mM NaCl, 50 mM TrisHCl pH 7.4, 1 mM EDTA pH 8.0, with protease inhibitor cocktail) to allow the binding of potential interacting proteins. Pulled down proteins together with aliquots of the input material and the supernatants were analyzed by Western blotting with antibodies directed against presynaptic proteins. For immunoprecipitation (IP), 10 μg of either anti-Syt1 antibodies (mouse clone 41.1, Synaptic Systems) or anti-Syt2 antibodies (mouse ZNP-1; Abcam) or mouse/rabbit control IgGs (Sigma-Aldrich) were pre-coated with Protein G Sepharose resin (GE Healthcare) overnight and incubated with total mouse brain lysate in IP buffer (150 mM NaCl, 50 mM Tris-HCl pH 7.4, 2 mM EDTA, 1% Triton X-100). After extensive washes in IP buffer and detergent-free IP buffer, samples were resolved by SDS-PAGE and subjected to western blotting with anti-PRRT2 antibodies.

RNA extraction and real time PCR. Total RNA was extracted by using the RNeasy Mini or Micro Kit (Qiagen) and then treated with DNase with the DNA-free DNA Removal kit (Ambion), following manufacturer's instructions. RNA concentration was quantified by using the Nanodrop-1000 spectrophotometer (Thermo Scientific). Retrotranscription was performed on equal amounts of RNA by using the M-MLV Reverse Transcriptase and random primers (Invitrogen), following manufacturer's instructions and including RT-negative controls. Real time PCR analyses were performed using the SYBR Green I Master mix (Roche), on a Lightcycler 480 (Roche), with the following protocol: 95°C for 5 min; 10 s at 95°C / 20 s at the specific annealing temperature (T_a) / 10 s at 72°C for 45 cycles; melting curve (heating ramp from 55°C to 95°C) in order to check for amplification specificity. The following primers (final concentration 0.25 μM) and annealing temperatures were used: *PRRT2* For-1 AGGTAGCCTAAGCCGTCATCC, *PRRT2* Rev-1 CAGGCTGTTCCGGGACATGAC, T_a=61°C; *PRRT2* For-2 CATCGCCTCCTGCGTCATCAAC, *PRRT2* Rev-2: CTCAGGCTCCCTTGGTCCCTAG, T_a=63°C; *H3f3a* For GTGAAGAAACCTCATCGTTACAGGCCTGGT, *H3f3a* Rev CTGCAAAGCACCAATAGCTGCACTCTGGAA, T_a=64°C; *Tbp* For GAGCTCTGGAATTGTACCGCAG, *Tbp* Rev CATGATGACTGCAGCAAATCGC, T_a=62°C; *Tubb3* For GCGTATACTACAATGAGGCCTCC,

Tubb3 Rev GTTGCCAGCACCACTCTGACC, T_a=63°C; *Hprt* For CAGACTGAAGAGCTACTGTAATG, *Hprt* Rev GGGCTGTACTGCTTAACCAGG, T_a=62°C. Relative quantification of *PRRT2* expression was made using the $2^{-\Delta\Delta Ct}$ method (Pfaffl, 2001), normalizing data to the geometric mean of three housekeeping transcripts (*H3f3a*, *Tbp*, *Hprt* for primary neurons and *H3f3a*, *Tbp*, *Tubb3* for tissue samples) (Vandesompele et al., 2002) and to a calibrator (developmental stage at which the peak of expression was observed: 10 DIV for primary hippocampal neurons, 14 DIV for primary cortical neurons and P60 for tissue samples).

Cell culture procedures. Low-density and autaptic hippocampal neurons were prepared from C57BL6/J mice as previously described (Baldelli et al., 2007). Animals were sacrificed by CO_2 inhalation, and 17/18-day embryos (E17-18) were removed immediately by cesarean section. In brief, hippocampi or cerebral cortices were dissociated by enzymatic digestion in 0.125% Trypsin for 20 min at 37°C and then triturated with a fire-polished Pasteur pipette. No antimitotic drugs were added to prevent glia proliferation. Autaptic neurons were prepared as described previously (Bekkers and Stevens, 1991; Chiappalone et al., 2009) with slight modifications. Dissociated neurons were plated at very low density (20 cells/mm^2) on microdots (40-300 μm in diameter) obtained by spraying a mixture of poly-D-lysine (0.1 mg/ml) and collagen (0.25 mg/ml) on petri dishes, previously pretreated with 0.15% agarose. Both glial cells and single autaptic neurons were present under this culture condition.

Immunocytochemistry and analysis of synapse density. Neurons were fixed at 14 DIV (7 days post-infection) in 4% formaldehyde, freshly prepared from paraformaldehyde, in 0.1 M phosphate buffer, pH 7.4 (PB) for 20 minutes at room temperature (RT) and immunostained for SNAP-25, synaptophysin, vGLUT1 and tGFP in various combinations. Briefly, after several washes in phosphate-buffered saline (PBS), cells were permeabilized and blocked for 30 minutes in 5% Normal Goat Serum (NGS), 0.1 % saponin in PBS and then incubated with primary antibodies diluted in 5% NGS and 0.1% saponin in PBS up to two hrs. Antibodies were used as follows: anti-SNAP-25 (1:200; Synaptic Systems), anti-synaptophysin-1 (1:200; Synaptic Systems), anti-VGLUT1 (1:1000; Synaptic Systems #135304), anti-tGFP (1:200; Origene #TA150041). Neurons were then washed twice in 0.1 % saponin or Triton X-100 in PBS and blocked for 10 min in 5 % NGS and 0.1 % saponin or Triton X-100 in PBS before being incubated in the same buffer with Alexa conjugated secondary antibodies (1:500, Invitrogen). After several washes in PBS coverslips were mounted using Prolong Gold anti-fade reagent with DAPI staining (Invitrogen). As a control for the staining and the acquisition procedure the primary antibodies were omitted. Images were acquired using a 63X objective in a Leica SP5 confocal (Leica Microsystem, Vienna).
For the analysis of synapse density, low density hippocampal neurons were infected at 7 DIV and processed for immunofluorescence at 14 DIV. Cells were rinsed once with Krebs–Ringer's solution (KRH)–EGTA (130 mM NaCl, 5 mM KCl, 1.2 mM KH_2PO_4, 1.2 mM $MgSO_4$, 2 mM $MgCl_2$, 2 mM EGTA, 25 mM Hepes, 6 mM glucose, pH 7.4), fixed for 15 minutes with 4% paraformaldehyde, 4% sucrose in 120 mM sodium phosphate buffer, pH 7.4, supplemented with 2 mM EGTA. Coverslips were rinsed twice with phosphate-buffered saline (PBS) and then incubated overnight at 4°C into a humidified chamber with the primary antibodies (mouse anti-Bassoon (1:200, Stressgen) and rabbit anti-Homer 1 (1:150, Synaptic Systems) diluted in goat serum dilution buffer (GSDB; 15% goat serum, 450 mM NaCl, 0.3% Triton X-100, 20 mM sodium phosphate buffer, pH 7.4). Cells were washed three times with PBS and then incubated with the appropriate secondary antibodies for 1 hour at room temperature. After three washes with PBS for 10 min each, coverslips were mounted with Dako fluorescence mounting medium. Specimens were acquired with an Axio Imager.A2 microscope (Zeiss, Oberkochen, Germany) and images were processed using the colocalization plugin of ImageJ: five dendrites per neuron were selected, and the colocalization analysis was performed between images after subtracting an appropriate threshold to evaluate the simultaneous presence of a pre- and a post-synaptic protein (Bsn and Homer 1, respectively). We considered the colocalization puncta with an area in the range of 0.1–1 μm^2, corresponding to an overlapping area of pre-synaptic and post-synaptic proteins to identify *bona fide* synaptic boutons. We counted the puncta present within 30 μm dendrite tract starting from the cell body. Data are referred to three independent experiments carried out in duplicate. Ten neurons for each sample were analyzed. Statistical analysis was performed using the Student's t-tests, comparing the Sh-RNA treated sample to the corresponding scramble control.

Live imaging of exo-endocytosis. Hippocampal cultures, grown onto poly-L-lysine-treated glass coverslips, were put in the stimulation chamber (Warner Instruments, Hamden, CT), immersed in Tyrode Solution (140 mM NaCl, 3 mM KCl, 2 mM $CaCl_2$, 1 mM $MgCl_2$, 10 mM HEPES-buffered to pH 7.4, 10 mM glucose) and located on the stage of an IX-81 motorized inverted epifluorescence microscope (Olympus, Hamburg, Germany). The imaging buffer was supplemented with glutamate receptor inhibitors (CNQX 10 μM; APV 50 μM; TOCRIS) to reduce spontaneous activity and prevent recurrent excitation during stimulation. An MT20 Hg-Xe lamp (Olympus) was used as light source with 480±20 nm excitation, 495 nm dichroic and 525±50 nm emission filters to detect the GFP (SypHy or tGFP) signal and 560±40 nm excitation, 585 nm dichroic and 630±75 nm

emission filters to detect the SypHy m-Orange2 or the m-Cherry signal. Time-lapses were acquired at 1 Hz for 100 s with an Orca-ER IEEE1394 CCD camera (Hamamatsu Photonics, Hamamatsu City, Japan) using a UplanSapo 60X1.35 NA oil-immersion objective (Olympus). Cells were maintained in a saline solution at room temperature through a laminar-flow perfusion and the selected field was stimulated after 10 seconds of baseline acquisition. Action potentials (AP) were evoked by passing 1-ms current pulses through platinum-iridium electrodes using a AM 2100 stimulator (AM-Systems, Carlsborg, WA). One image on GFP/mCherry channel was acquired at the beginning of each experiment to verify the PRRT2 silencing on the processes to be analyzed by pHluorin assay. Circular ROIs of 1.7 mm diameter were positioned manually at the center of each responsive synapse.

To evaluate the effects of PRRT2 silencing on SV trafficking, stimulation protocols were applied to estimate the release from either the readily releasable pool (40 action potentials @20Hz) or the recycling pool (400 action potentials@20Hz) (Fassio et al., 2011). After 10 s of baseline acquisition (F_0), neurons were stimulated in the presence or absence of 1 μM bafilomycin and finally subjected to alkalinization by perfusion with 50 mM NH_4Cl (F_{max}). Images were analyzed using the Xcellence RT software (Olympus). The total increase in the fluorescence signal (ΔF) was calculated by subtracting F_0 and the ΔF was normalized to the fluorescence value obtained by alkalization of the entire vesicle pool using NH_4Cl (ΔF_{max}). The time constant of endocytosis (τ decay phase) was calculated by the fitting the post-stimulus decay with a single-exponential function. To get release rates, the traces recorded during stimulation were fitted as single exponential functions and the relative kinetics were plotted as time constants of release (τ rising phase). N refers to the number of coverslips analyzed from 3-4 different preparations. Data were collected from 20-40 boutons per coverslip. Exocytosis studied by grossly corresponds to the time integral of release in response to a long-lasting (20 sec) high-frequency (20 Hz) stimulation that includes the synchronous release, its dynamics of depression and the asynchronous release. Therefore, it cannot be compared with the amplitude of EPSCs evoked by single Aps, but rather with the integral of the charge released during sustained high-frequency stimulations.

Transmission Electron Microscopy. Autaptic hippocampal neurons and low-density cultures of hippocampal neurons were infected at 7–11 DIV with either scramble shRNA or PRRT2 shRNAs and processed for transmission electron microscopy (TEM). To unambiguously identify transduced neurons, we correlated the tGFP fluorescence signal with EM analysis. To do so, we identified tGFP-positive neurons at the epifluorescence microscope and marked their position by scratching the coverslip around the neuron with a pencil point diamond-tip glass-cutter. Neurons were fixed at 14–18 DIV with 1.2% glutaraldehyde in 66 mM sodium cacodylate buffer, post-fixed in 1% OsO_4, 1.5% $K_4Fe(CN)_6$, 0.1 M sodium cacodylate, *en bloc* stained with 1% uranyl acetate, dehydrated, and flat embedded in epoxy resin (Epon 812, TAAB). After baking for 48 hrs, the position of the scratches on coverslips was detected under a stereomicroscope and marked on the resin block with a marker pen. The glass coverslip was removed from the Epon block by thermal shock and isolated autaptic neurons were identified by means of a stereomicroscope with the help of the pen marker positional clue. Embedded neurons were the excised from the block, and mounted on a cured Epon block for sectioning using an EM UC6 ultramicrotome (Leica Microsystems). Ultrathin sections (60–70 nm thick) were collected on 200-mesh copper grids (EMS) and observed with a JEM-1011 electron microscope (Jeol, Tokyo, Japan) operating at 100 kV using an ORIUS SC1000 CCD camera (*Gatan,* Pleasanton, CA). Low-density cultured neurons were studied using the same procedure. For each experimental condition, at least 30 images of synapses were acquired at 10,000x magnification (sampled area per experimental condition: 36 μm^2). Synaptic profile area, SV number, and distribution relative to the active zone (AZ) were determined using ImageJ software. For the 3D reconstruction, the standard TEM sample preparation protocol was followed, and samples were embedded in Epon resin. Serial 60-nm-thick sections were collected on carbon-coated copper slot formvar and carbon-coated grids, and serial synaptic profiles acquired. Serial sections were aligned using the Midas of IMOD. Synapses with one single AZ, at least one docked SV and >100 but <800 total SVs were reconstructed with the software IMOD. The quantitative analysis of synaptic density was performed using NIH ImageJ software. A counting frame of known area (8.67 x 5.78 μm) was created using Adobe Illustrator CS5 software and was superimposed to the hippocampal image; only the asymmetric and symmetric synapses present in each image and intersecting the right and superior edges of the frame were counted to obtain the synapse density. Volume density of the synapses was calculated from the two-dimensional count of synaptic profile, using the stereological formula $N_{vs}= N_a/(H+t)$, where N_a is the number of synapses per unit area, t is the section thickness and H is the mean tangent diameter ($H = (\pi D)/4$, where D is the mean diameter of the synaptic contact), as previously described (Kurt et al., 2004). For the analysis of synaptic ultrastructure under stimulation, primary hippocampal neurons were infected as described above. Infected neurons were field stimulated at 14-18 DIV (7 days post-infection) with a train stimulation protocol of 30 s @ 10Hz with an insulated pulse stimulator (A-M Systems). Experiments were performed in Tyrode's buffer containing 50 μM APV and 10 μM CNQX. Immediately after the end of the train (0 s) and after 20 min recovery, neurons were fixed for electron microscopy in 1.25% glutaraldehyde in 66 mM sodium cacodylate buffer at 37 °C and then processed as described for the

conventional transmission electron microscopy. Under these conditions, we estimated a complete fixation of synapses within 1-3 s from fixative addition (Leung, 1994).

Patch-clamp recordings. Patch pipettes, prepared from thin-borosilicate glass (Hilgenberg, Mansfield, Germany), were pulled and fire-polished to a final resistance of 2-4 MΩ when filled with standard internal solution. Evoked postsynaptic currents (ePSCs) were recorded using a double EPC-10 amplifier (HEKA Electronic, Lambrecht, Germany). For whole-cells recordings, cells were maintained in a standard external solution containing (in mM): 140 NaCl, 2 $CaCl_2$, 1 $MgCl_2$, 4 KCl, 10 glucose, 10 HEPES (pH 7.3 with NaOH). When using external solutions with different ionic composition, salts were replaced equimolarly. To record eEPSCs, where otherwise not indicated, D-(-)-2-amino-5-phosphonopentanoic acid (D-AP5; 50 μM; Tocris, Bristol, UK) and bicuculline methiodide (30 μM, Tocris) were added to the Tyrode solution to block N-methyl-D-aspartate (NMDA) and $GABA_A$Rs, respectively. To record eIPSCs, 6-cyano-7-nitroquinoxaline-2,3-dione (CNQX; 10 μM) and CGP 58845 (10 μM) were added to the external solution to block non-NMDA and $GABA_B$Rs, respectively. The standard internal solution was (in mM): 126 KGluconate, 4 NaCl, 1 $MgSO_4$, 0.02 $CaCl_2$, 0.1 BAPTA, 15 Glucose, 5 HEPES, 3 ATP, 0.1 GTP (pH 7.2 with KOH). For experiments in which eIPSCs were studied, following internal solution was used (in mM): 120 KGluconate, 4 NaCl, 20 KCl, 1 $MgSO_4$, 0.1 EGTA, 15 Glucose, 5 HEPES, 3 ATP, 0.1 GTP (pH 7.2 with KOH). All experiments were performed at room temperature (22-24 °C). Neurons were voltage-clamped at -70 mV. Unclamped action potentials (APs) were evoked by a brief depolarization of the cell body to +40 mV for 0.5 ms @ 0.1 Hz. eE/IPSCs were acquired at 10-20 kHz sample frequency and filtered at half the acquisition rate with an 8-pole low-pass Bessel filter. Recordings with leak currents >100 pA or series resistance >20 MΩ were discarded. Data acquisition was performed using PatchMaster programs (HEKA Elektronic, Lambrecht, Germany). eE/IPSCs were inspected visually, and only those that were not contaminated by spontaneous activity were considered. To calculate the peak current during an isolated stimulus or a train of stimuli, we first subtracted an averaged trace containing the stimulus artifact and the AP current, but lacking any discernable synaptic current (i.e. synaptic failures). Such traces were easily identified toward the end of a train of stimuli, when synaptic depression was maximal. These traces were averaged and scaled to the peak Na^+ current contaminating the eE/IPSCs.

Short-term plasticity paradigms. To analyze paired-pulse ratio (PPR), two brief depolarizing pulses were applied to autaptic neurons at 50 ms time intervals. For each couple of eE/IPSCs, PPR was calculated as the ratio I_2/I_1, where I_1 and I_2 are the amplitudes of the eEPSCs/eIPSCs evoked by the conditioning (1) and test (2) stimuli, respectively. To correctly estimate the amplitude of I_2, the baseline of I_2 was defined as the final value of the decay phase of I_1 and the amplitude of I_2 was calculated by subtracting the residual amplitude of I_1 from the peak value of I_2. For the evaluation of the synaptic responses during tetanic stimulation, the time interval was shorter than the time needed for an eEPSC to return to baseline, so eEPSCs overlapped partially. Then, to correctly estimate the EPSC amplitude, the baseline of each event was defined as the final value of the decay phase of the preceding eEPSC and the amplitude of eEPSC number n was calculated by subtracting the residual amplitude of the previous eEPSCs from its peak value. For the evaluation of post-tetanic potentiation (PTP), autaptic neurons were depolarized with short-trains of stimuli (2 s @ 40 Hz). The maximal PTP induced by the tetanic stimulation was determined by measuring the maximal amplitude of the eEPSCs, usually obtained 10 or 20 s after the end of the train. The time-course of the PTP was followed with a stimulation frequency to 0.1 Hz.

Cumulative eEPSC amplitude analysis. The size of the readily releasable pool of synchronous release (RRP_{syn}) and the probability that any given SV in the RRP will be released (Pr) were calculated using the cumulative amplitude analysis (Schneggenburger et al.,1999 and 2002; Baldelli et al., 2007). The cumulative amplitude plot was determined by summing up peak EPSC amplitudes during 40 repetitive stimuli applied at 40 Hz. This analysis assumes that depression during the steady-state phase is limited by a constant recycling of SVs and an equilibrium occurs between released and recycled SVs and that Pr_{syn} during the train approaches the 1 value (Schneggenburger et al., 1999). Assuming that the slow linear rise is due to the equilibrium between the release-induced depletion and the constant replenishment of the RRP, back-extrapolation of the linear portion to time zero yields a rough estimation of the size of the RRP current of synchronous release (RRP_{syn}), while the release probability Pr is calculated as the ratio between the first eEPSC (I1) and the RRP_{syn}. The number of data points for the linear fitting of the steady-state phase was evaluated by calculating the best fit including the maximal number of data points starting from the last data point (generally the last 15-20 data points). To estimate the number of SVs (N_{syn}) forming the RRP_{syn}, RRP_{syn} was divided by the unitary current (Q_{av}, corresponding to the average amplitude of autaptic mEPSCs).

Estimation of RRP_{total} by the hypertonic sucrose method. The RRP size of the total release (RRP_{tot}) was estimated using a hypertonic stimulation (Rosenmund and Stevens, 1996; Baldelli et al., 2007). Autaptic neurons were voltage clamped at –70 mV and stimulated by a 1 ms voltage step to 20/100 mV applied at a frequency of 0.05 Hz, evoking an isolated EPSC. One minute later, a hypertonic extracellular solution (supplemented with 500 mM sucrose; Yamaguchi et al., 2002) was focally micro-applied to the same neuron for 6 s. The charge transfer in the transient part of the synaptic current induced by the sucrose application was

measured as RRP_{tot}. The number of RRP_{tot} quanta was then calculated by dividing the RRP_{tot} by the unitary mEPSC charge. The experiments were performed by constantly superfusing the autaptic neurons with a Tyrode external solution. The perfusion solution (flow rate ≈ 200 µl/min) could be rapidly changed (50/60 ms) and puffs of hypertonic solution were applied over controlled periods of time (6 sec). The tip of the perfusion pipette (50/100 µm) was placed close to the soma (80/150 µm).

Asynchronous and spontaneous release analysis. The delayed asynchronous release of the excitatory autapses was evoked by a tetanic stimulation lasting 2 s @ 40 Hz. Asynchronous release was estimated by measuring the area (pA*ms) of the spontaneous EPSCs that follow the stimulation train, in 9 consecutive time-windows lasting 1 s each, for a total time-period of 9 seconds. Miniature EPSCs (mEPSC) were collected from low-density and autaptic neurons incubated in the presence of tetrodotoxin (TTX, 1 µM; Tocris) to block spontaneous action potentials propagation. mEPSC analysis was performed by using the Minianalysis program (Synaptosoft, Leonia, NJ). The amplitude and frequency of mPSCs were calculated using a peak detector function using appropriate threshold amplitude and threshold area.

SUPPLEMENTAL DISCUSSION

The paroxysmal nature of the attacks and the variable phenotypes in patients bearing PRRT2 loss-of-function mutations, together with the association with epilepsy and migraine, suggest the presence of an acute defect in the excitation/inhibition balance. In PRRT2-KD, we observed an impairment in synaptic transmission in both glutamatergic and GABAergic synapses, although the effect seemed more severe for inhibitory transmission. To account for an excitation/inhibition imbalance that explains the human phenotypes, two possibilities might be considered. It is possible that the regional distribution of PRRT2 among excitatory and inhibitory neurons varies in the various brain areas, bringing about shifts in the excitation/inhibition balance associated with PRRT2 haploinsufficiency. An alternative explanation is that the more pronounced effects of PRRT2 KD in inhibitory neurons may engender an anomalous excitation/inhibition imbalance under rare conditions of heightened network activity, due to positive feedback cycles. Such positive feedback cycles lead to activity-dependent reductions in the efficacy of neuronal inhibition through the phenomenon of activity-dependent disinhibition, occasionally crossing the threshold needed to generate an ictal state (Staley, 2015).

SUPPLEMENTAL REFERENCES

Baldelli P, Fassio A, Valtorta F, Benfenati F (2007) Lack of synapsin I reduces the readily releasable pool of synaptic vesicles at central inhibitory synapses. J Neurosci 27, 13520-13531

Bekkers JM, Stevens CF (1991) Excitatory and inhibitory autaptic currents in isolated hippocampal neurons maintained in cell culture. Proc Natl Acad Sci USA 88: 7834-7838

Chiappalone M, Casagrande S, Tedesco M, Valtorta F, Baldelli P, Martinoia S, Benfenati F (2009) Opposite changes in glutamatergic and GABAergic transmission underlie the diffuse hyperexcitability of synapsin I-deficient cortical networks. Cereb Cortex 19, 1422-1439

Fassio A, Patry L, Congia S, Onofri F, Piton A, Gauthier J, Pozzi D, Messa M, Defranchi E, Fadda M, Corradi A, Baldelli P, Lapointe L, St-Onge J, Meloche C, Mottron L, Valtorta F, Khoa Nguyen D, Rouleau GA, Benfenati F, Cossette P (2011) SYN1 loss-of-function mutations in autism and partial epilepsy cause impaired synaptic function. Hum Mol Genet 20, 2297-2307

Feligioni M, Holman D, Haglerod C, Davanger S, Henley JM (2006) Ultrastructural localisation and differential agonist-induced regulation of AMPA and kainate receptors present at the presynaptic active zone and postsynaptic density. J Neurochem 99, 549-560

Huttner WB, Schiebler W, Greengard P, De Camilli P (1983) Synapsin I (protein I), a nerve terminal-specific phosphoprotein. III. Its association with synaptic vesicles studied in a highly purified synaptic vesicle preparation. J Cell Biol 96, 1374-1388

Kurt MA, Kafa MI, Dierssen M, Davies DC (2004) Deficits of neuronal density in CA1 and synaptic density in the dentate gyrus, CA3 and CA1, in a mouse model of Down syndrome. Brain Res 1022, 101-109

Leung A (1994) Fixation. In "Laboratory histopathology: a complete reference" (AE Woods and RC Ellis eds.) Churchill Livingstone, New York.

Pfaffl MW (2001) A new mathematical model for relative quantification in real-time RT-PCR. Nucleic Acids Res 29: e45

Phillips GR, Huang JK, Wang Y, Tanaka H, Shapiro L, Zhang W, Shan WS, Arndt K, Frank M, Gordon RE, Gawinowicz MA, Zhao Y, Colman DR (2001) The presynaptic particle web: ultrastructure, composition, dissolution, and reconstitution. Neuron 32, 63-77

Rosenmund C, Stevens CF (1996) Definition of the readily releasable pool of vesicles at hippocampal synapses. Neuron 16, 1197-1207

Schneggenburger R, Meyer AC, Neher E (1999) Released fraction and total size of a pool of immediately available transmitter quanta at a calyx synapse. Neuron 23, 399-409

Schneggenburger R, Sakaba T, Neher E (2002) Vesicle pools and short-term synaptic depression: lessons from a large synapse. Trends Neurosci 25, 206-212

Staley K (2015) Molecular mechanisms of epilepsy. Nature Neuroscience 18, 367-372

Vandesompele J, De Preter K, Pattyn F, Poppe B, Van Roy N, De Paepe A, Speleman F (2002) Accurate normalization of real-time quantitative RT-PCR data by geometric averaging of multiple internal control genes. Genome Biol 3: research0034.1–research0034.11

Yamaguchi K, Tanaka M, Mizoguchi A, Hirata Y, Ishizaki H, Kaneko K, Miyoshi J, Takai Y (2002) A GDP/GTP exchange protein for the Rab3 small G protein family up-regulates a postdocking step of synaptic exocytosis in central synapses. Proc Natl Acad Sci USA 99, 14536-14541
